# A new ALK inhibitor overcomes resistance to first‐ and second‐generation inhibitors in NSCLC

**DOI:** 10.15252/emmm.202114296

**Published:** 2021-11-30

**Authors:** Yue Lu, Zhenzhen Fan, Su‐Jie Zhu, Xiaoxing Huang, Zhongji Zhuang, Yunzhan Li, Zhou Deng, Lei Gao, Xuehui Hong, Ting Zhang, Li Li, Xihuan Sun, Wei Huang, Jingfang Zhang, Yan Liu, Baoding Zhang, Jie Jiang, Fu Gui, Zheng Wang, Qiyuan Li, Siyang Song, Xin Huang, Qiao Wu, Lanfen Chen, Dawang Zhou, Jianming Zhang, Cai‐Hong Yun, Liang Chen, Xianming Deng

**Affiliations:** ^1^ State Key Laboratory of Cellular Stress Biology Innovation Center for Cell Signaling Network School of Life Sciences Xiamen University Xiamen China; ^2^ Institute of Life and Health Engineering Jinan University Guangzhou China; ^3^ Department of Biochemistry and Biophysics Institute of Systems Biomedicine Peking University Health Science Center Beijing China; ^4^ Beijing Key Laboratory of Tumor Systems Biology School of Basic Medical Sciences Peking University Health Science Center Beijing China; ^5^ Institute for Translational Medicine College of Medicine Qingdao University Qingdao China; ^6^ Department of Gastrointestinal Surgery Affiliated Zhongshan Hospital of Xiamen University Xiamen China; ^7^ National Institute for Data Science in Health and Medicine School of Medicine Xiamen University Xiamen China; ^8^ Division of Drug Discovery Hongyun Biotech Co., Ltd. Nanjing China; ^9^ National Research Center for Translational Medicine Ruijin Hospital Shanghai Jiaotong University School of Medicine Shanghai China; ^10^ Present address: Key Laboratory of Gastrointestinal Cancer (Fujian Medical University) Ministry of Education Fuzhou China

**Keywords:** acquired resistance mutations, ALK inhibitor, crizotinib, EML4‐ALK, nonsmall cell lung cancer, Cancer, Pharmacology & Drug Discovery, Respiratory System

## Abstract

More than 60% of nonsmall cell lung cancer (NSCLC) patients show a positive response to the first ALK inhibitor, crizotinib, which has been used as the standard treatment for newly diagnosed patients with ALK rearrangement. However, most patients inevitably develop crizotinib resistance due to acquired secondary mutations in the ALK kinase domain, such as the gatekeeper mutation L1196M and the most refractory mutation, G1202R. Here, we develop XMU‐MP‐5 as a new‐generation ALK inhibitor to overcome crizotinib resistance mutations, including L1196M and G1202R. XMU‐MP‐5 blocks ALK signaling pathways and inhibits the proliferation of cells harboring either wild‐type or mutant EML4‐ALK *in vitro* and suppresses tumor growth in xenograft mouse models *in vivo*. Structural analysis provides insights into the mode of action of XMU‐MP‐5. In addition, XMU‐MP‐5 induces significant regression of lung tumors in two genetically engineered mouse (GEM) models, further demonstrating its pharmacological efficacy and potential for clinical application. These preclinical data support XMU‐MP‐5 as a novel selective ALK inhibitor with high potency and selectivity. XMU‐MP‐5 holds great promise as a new therapeutic against clinically relevant secondary ALK mutations.

## Graphical abstract

Despite the clinical success of ALK inhibitors in NSCLC, multiple drug‐resistant mutations in ALK are inevitably reported. XMU‐MP‐5 overcomes resistance to first and second generation ALK inhibitors *in vitro* and *in vivo*, thus holds great promise for the therapeutic use against ALK‐positive NSCLC.

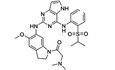

**The paper explained**

**Problem**

Despite the clinical success of first‐ and second‐generation ALK inhibitors in NSCLC, multiple drug‐resistant mutations in ALK, such as the gatekeeper mutation L1196M and the most refractory mutation, G1202R, are inevitably reported.

**Results**

We report the discovery of a new class of pharmacological agents (represented by XMU‐MP‐5) as potent and selective ALK inhibitors *in vitro* and *in vivo*. XMU‐MP‐5 effectively overcomes secondary ALK mutations conferring resistance to first‐ and second‐generation ALK inhibitors, including L1196M and G1202R. Using X‐ray crystallography, we revealed the unique binding mode of XMU‐MP‐5 and ALK, which contributes to the high affinity and selectivity of XMU‐MP‐5 for ALK and its mutants. We further demonstrated that XMU‐MP‐5 substantially repressed tumor growth in mouse xenograft models driven by ALK and its mutants. Most importantly, XMU‐MP‐5 also induced striking tumor shrinkage in a GEM model with lung‐specific doxycycline‐inducible EML4‐ALK and EML4‐ALK(L1196M) expression.

**Impact**

With high selectivity and safety, XMU‐MP‐5 represents a novel scaffold that could be optimized as therapeutic agents for NSCLC with acquired resistance to first and second generation ALK inhibitors such as those harboring the ALK L1196M or G1202R mutations.

## Introduction

Lung cancer is the leading cause of cancer‐related death worldwide (Torre *et al*, 2015), and nonsmall cell lung cancer (NSCLC) accounts for approximately 85% of lung cancer cases (Inamura, 2017). Lung adenocarcinoma is the most common subtype of lung cancer. Many oncogenic drivers of adenocarcinoma have been found in the clinic, such as the epidermal growth factor receptor (EGFR) and anaplastic lymphoma kinase (ALK) (Bayliss *et al*, 2016). In 1994, ALK was originally discovered in an anaplastic large cell lymphoma (ALCL) cell line, in which the ALK gene was fused with the nucleophosmin 1 (NPM1) gene, resulting in a new fusion protein, NPM‐ALK (Morris *et al*, 1994). Since then, more than 20 different ALK fusion proteins have been found in different cancers, for example, EML4‐ALK in NSCLC. EML4‐ALK was discovered in 2007 and is produced via fusion of the ALK gene to the echinoderm microtubule‐associated protein‐like 4 (EML4) gene (Holla *et al*, 2017), resulting in constitutive activation of ALK and, in turn, to dysregulation of signaling pathways in the host cell, leading to tumorigenesis (Golding *et al*, 2018). Crizotinib was approved by the FDA in 2011 as the first ALK inhibitor. Clinical data have demonstrated that crizotinib is superior to cytotoxic chemotherapy in patients with ALK‐positive NSCLC (Shaw *et al*, 2013). Although crizotinib initially results in significant clinical benefits to patients, disease inevitably progresses after treatment because of acquired resistance mutations (Tucker *et al*, 2015). Mechanisms of acquired resistance are classified into two types: ALK‐dependent resistance mechanisms, such as ALK amplification and ALK secondary resistance mutations, and ALK‐independent resistance mechanisms, including activation of bypass signaling pathways and lineage changes (Romanidou *et al*, 2016).

In a previous study, secondary ALK resistance mutations were identified in 20–30% of post‐crizotinib treatment samples (Awad & Shaw, 2014). Among these mutations, L1196M and G1269A are the most commonly detected in the clinic (Lin *et al*, 2017). To overcome crizotinib resistance mutations, second‐generation ALK inhibitors such as ceritinib, alectinib, and brigatinib were developed and approved successively; all of these tyrosine kinase inhibitors (TKIs) show potency against L1196M and G1269A (Sakamoto *et al*, 2011; Friboulet *et al*, 2014; Kodama *et al*, 2014; Zhang *et al*, 2016a, b). Unsurprisingly, patients inevitably relapse on these second‐generation inhibitors, and approximately 50–70% acquire secondary ALK resistance mutations. G1202R is a solvent front mutation in the kinase domain and impairs the binding of all first‐ and second‐generation ALK inhibitors by introducing steric hindrance; thus, it has become the ALK mutation conferring the strongest clinical resistance (Gainor & Shaw, 2013; Katayama *et al*, 2015). Although G1202R is less frequently detected in post‐crizotinib treatment samples (2%), it is the predominant resistance mutation in patients treated with second‐generation ALK inhibitors (40–65%) (Katayama *et al*, 2012; Ou *et al*, 2014; Gainor *et al*, 2016). The third‐generation ALK inhibitor lorlatinib was approved by the FDA in 2018 for the treatment of ALK‐positive NSCLC (Syed, 2019); it showed promising activity against all TKI resistance mutations in ALK in both cellular assays and *in vivo* mouse models, and most importantly, it could overcome the G1202R mutation (Gainor *et al*, 2016). As lorlatinib is the only ALK inhibitor on the market that may overcome the abovementioned high‐frequency ALK resistance mutations—especially G1202R—in the clinic, more effective new‐generation ALK inhibitors for clinical use are needed.

In this study, we aimed to develop a new chemical entity to overcome drug‐resistant NSCLC with secondary mutations in ALK. Herein, we demonstrated that XMU‐MP‐5 exhibited high kinase selectivity superior to that of current clinical ALK TKIs. It effectively overcame secondary ALK mutations, including L1196M and G1202R, *in vitro* and suppressed tumor growth in xenograft mouse models *in vivo*. Moreover, XMU‐MP‐5 effectively suppressed autochthonous lung cancer in two genetically engineered mouse (GEM) models.

## Results

### Discovery of XMU‐MP‐5 as a novel selective ALK inhibitor

**Figure 1:**
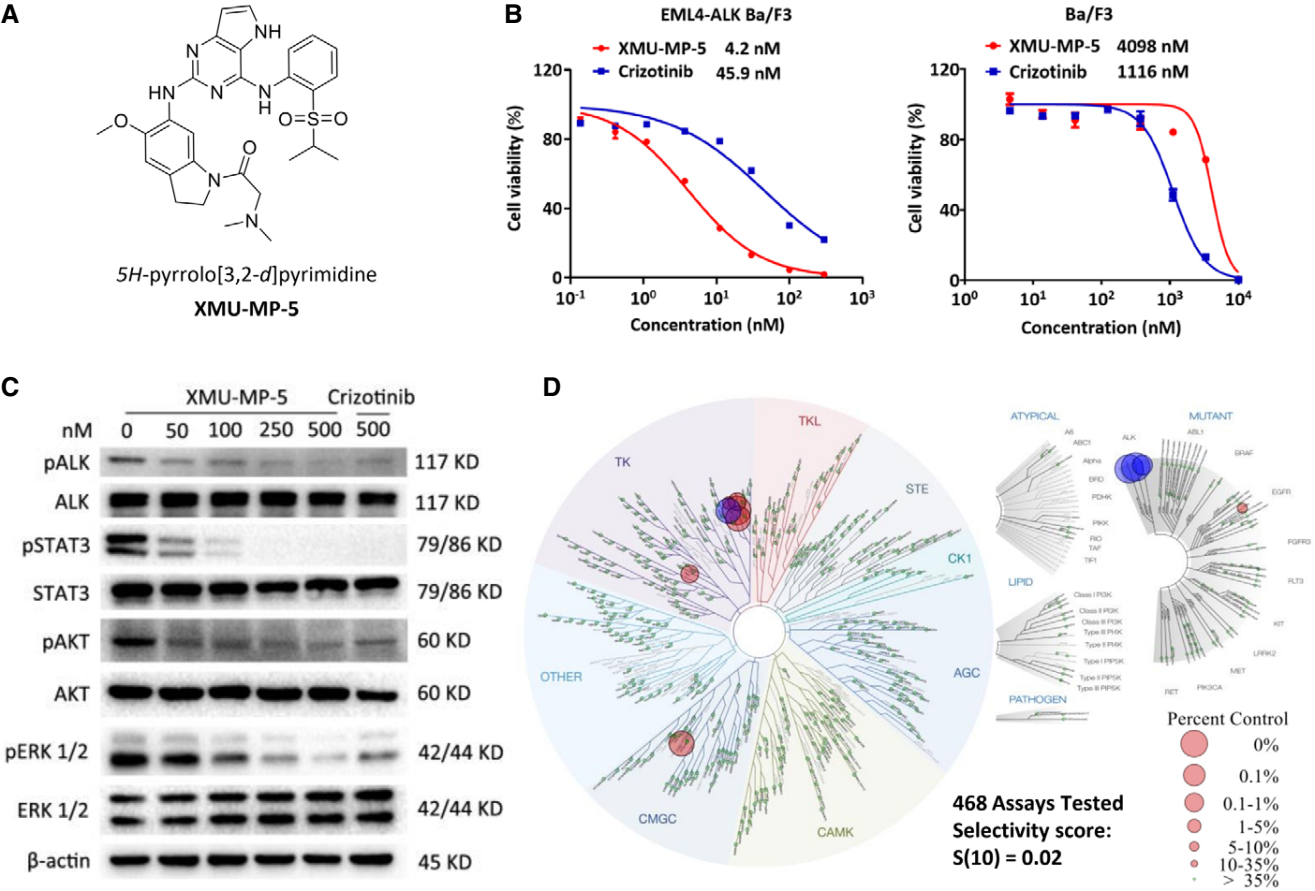
XMU‐MP‐5 is a potent and selective ALK inhibitor Chemical structure of XMU‐MP‐5.Antiproliferative activities of XMU‐MP‐5 and crizotinib against EML4‐ALK Ba/F3 and parental Ba/F3 cells, respectively. Cells were treated with increasing concentration of each drug for 48 h and then analyzed by MTS assay. Each concentration was performed in triplicate. Data were presented as Mean ± SEM from three independent experiments (*n* = 3).XMU‐MP‐5 downregulates ALK signaling pathways in EML4‐ALK Ba/F3 cells. Cells were treated with XMU‐MP‐5 or crizotinib for 4 h, and then analyzed by immunoblotting.The KINOMEscan result of XMU‐MP‐5 against a panel of 468 kinases. Selectivity score S(10) and kinase hits of XMU‐MP‐5 with ctrl% ≤ 10% were shown. ALK and its mutants were highlighted in blue (see Appendix Table S1 for the full list and see Experimental Section for the detailed kinase group names). Source data are available online for this figure.

To identify a novel small molecular ALK inhibitor with high potency, we performed differential cytotoxicity screening against EML4‐ALK‐transformed Ba/F3 cells with an in‐house compound library designed to target the ATP‐binding pocket of the ALK kinase domain. The *5H*‐pyrrolo[3,2‐*d*]pyrimidine compound XMU‐MP‐5, which represents a distinct chemical scaffold different from those of the other ALK inhibitors, was developed through iterative medicinal chemistry optimization (Fig 1A and Appendix Fig S1). XMU‐MP‐5 exhibited impressive potency against EML4‐ALK Ba/F3 cells, with an IC_50_ value of 4.153 nM, while it exhibited negligible antiproliferative activity against parental wild‐type Ba/F3 cells. The differential cytotoxicity of XMU‐MP‐5, exemplified by the IC_50_ (WT‐Ba/F3)/IC_50_ (EML4‐ALK‐Ba/F3) ratio, was nearly 40‐fold greater than that of the well‐established ALK inhibitor crizotinib (Fig 1B). In addition, treatment with XMU‐MP‐5 significantly inhibited the phosphorylation of ALK in EML4‐ALK Ba/F3 cells in a dose‐dependent manner (Fig 1C), accompanied by decreases in the levels of ALK‐mediated downstream signaling effectors, including p‐STAT3, p‐ERK, and p‐AKT (Roskoski, 2013; Zhang *et al*, 2016a, b). In addition, XMU‐MP‐5 significantly induced apoptosis in EML4‐ALK Ba/F3 cells did not affect apoptosis in wild‐type Ba/F3 cells (Appendix Fig S2). Collectively, these results suggest that XMU‐MP‐5 is a potent ALK inhibitor.

The kinase selectivity of XMU‐MP‐5 was further investigated by a KINOMEscan assay in a panel of 468 kinases and mutants (Eurofins DiscoverX Corporation, San Diego, CA) (Fig 1D and Appendix Table S1) (Karaman *et al*, 2008). The KINOMEscan results are reported as percentages of the DMSO negative control signal (ctrl%), which is set to 100%, and a lower number indicates a higher binding affinity. At 1 μM, only 10 kinase hits with ctrl%≤10% for XMU‐MP‐5 were detected. Additionally, the dissociation constant (*K*_d_) of XMU‐MP‐5 for these top hits was determined (Table 1). Among the hits, XMU‐MP‐5 exhibited the highest binding affinities for ALK and its mutants ALK(C1156Y) and ALK(L1196M), with *K*_d_ values of 0.57, 0.4, and 0.77 nM, respectively. The selectivity score S(10) for XMU‐MP‐5 was 0.02, calculated by dividing the number of kinases with ctrl% ≤ 10% (*n* = 10) by the total number of kinases tested (i.e., 468). Considering the reported S scores of other ALK TKIs (Sakamoto *et al*, 2011; Huber *et al*, 2014; Zhang *et al*, 2016a, b; Jang *et al*, 2017), these data suggest that XMU‐MP‐5 is a highly selective ALK inhibitor.

**Table 1:** *K*_d_’s of kinase hits for XMU‐MP‐5 with ctrl% ≤ 10%.

DiscoveRx gene symbol	Percent control (%)	*K*_d_ (nM)
ALK	0.45	0.57
ALK(C1156Y)	0	0.4
ALK(L1196M)	0	0.77
CDC2L5	0.9	> 30,000
EGFR(T790M)	9.1	610
FER	4.9	61
IGF1R	1.1	10
INSR	0.1	3.8
INSRR	0.8	20
LTK	5.1	1.1

### *In* *vitro* and *in vivo* efficacy of XMU‐MP‐5 in ALK‐positive cells

**Figure 2:**
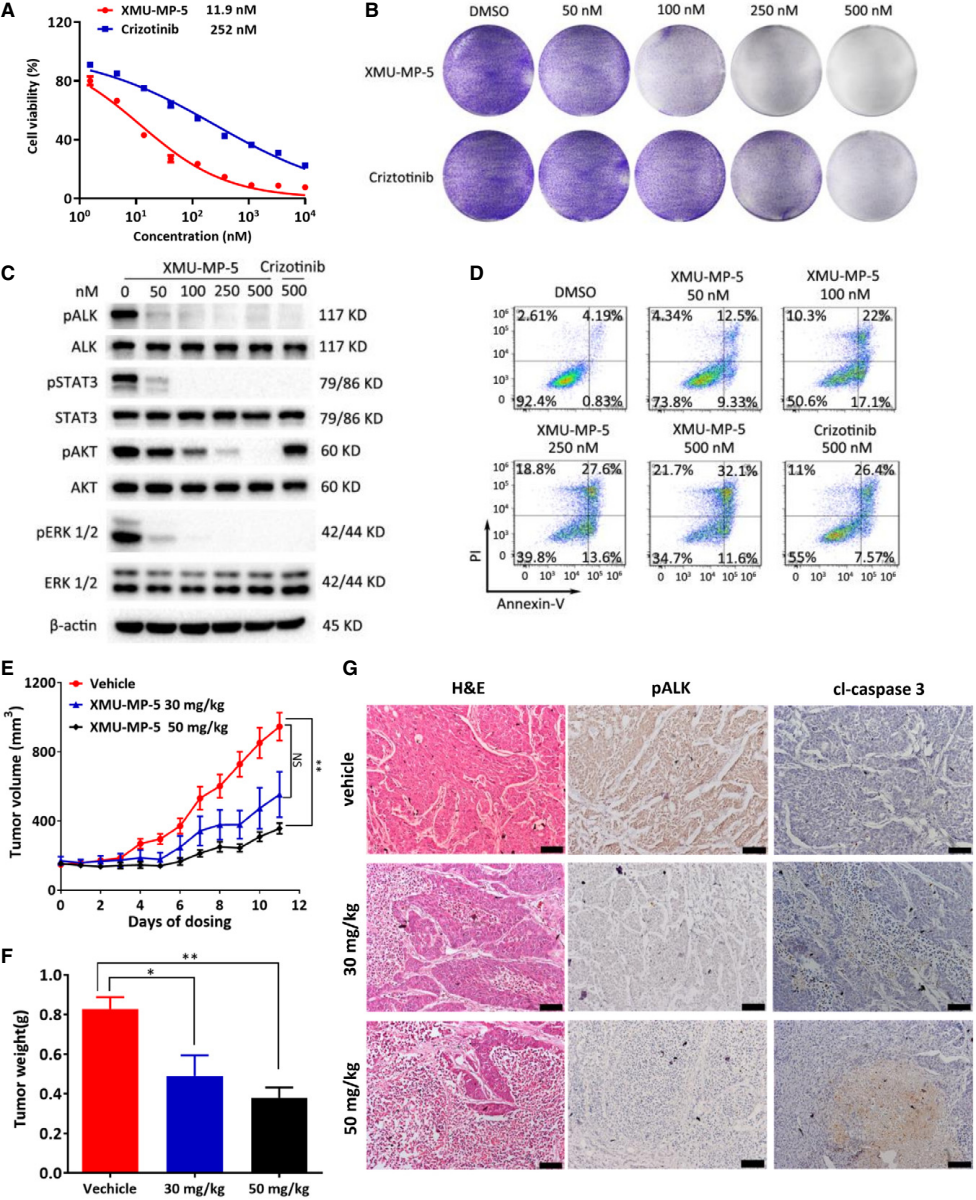
The antiproliferative activity of XMU‐MP‐5 against H3122 cells both *in vitro* and *in vivo* H3122 cells were treated with XMU‐MP‐5 or crizotinib for 72 h. Cell viability was measured by MTS assay. Each concentration was performed in triplicate. Data were presented as Mean ± SEM from three independent experiments (*n* = 3).XMU‐MP‐5 inhibited colony formation of H3122 cells. H3122 cells were treated with XMU‐MP‐5 or crizotinib for 18 days and then stained with crystal violet.XMU‐MP‐5 suppressed ALK signaling pathway in H3122 cells.XMU‐MP‐5 induced cell apoptosis in H3122 cells. Cells were treated as indicated for 72 h and then assayed by Annexin‐V‐FLUOS/PI staining.Comparison of tumor volume of different treatment groups in 11 days. BALB/c nude mice bearing H3122 xenograft tumors were treated with vehicle, XMU‐MP‐5 and crizotinib by tail vein injection once daily. Data were shown as mean ± SEM (*n* = 6). Statistical comparisons were performed using a two‐tailed, unpaired Student’s *t* test. N.S., *P* = 0.1063; **P = 0.0097.Comparison of tumor weight of each group at end point. Data were shown as mean ± SEM (*n* = 6). Statistical comparisons were performed using a one‐way ANOVA with Dunnett’s test. *P = 0.0177, **P = 0.002.Representative images of tumors analyzed by H&E and IHC staining. Scale bars, 100 μm. Source data are available online for this figure.

We next evaluated the antitumor activity of XMU‐MP‐5 in EML4‐ALK‐positive H3122 lung cancer cells. XMU‐MP‐5 potently inhibited H3122 cell proliferation, with an IC_50_ value of 11.85 nM (Fig 2A). This antiproliferative activity of XMU‐MP‐5 was further confirmed by a colony formation assay (Fig 2B). The *in vitro* antitumor activity of XMU‐MP‐5 might be attributed to its high potency in suppressing ALK signaling pathways and its ability to induce apoptosis (Fig 2C and D, and Appendix Fig S3A). These data indicate that XMU‐MP‐5 is more effective than crizotinib against ALK‐positive NSCLC cells. Although XMU‐MP‐5 was effective against H3122 cells, it showed negligible activity in ALK‐negative NSCLC cell lines, including A549 (KRAS mutant), H1299 (EGFR wild‐type), and PC9 (EGFR exon 19 deletion) (Appendix Fig S3B and C). More importantly, neither did it affect the nontumorigenic lung cell line MRC‐5, human peripheral blood mononuclear cells (PBMCs) from two healthy donors, or human T cells (Appendix Fig S4).

The pharmacokinetic properties of XMU‐MP‐5 were evaluated in ICR mice (Appendix Table S3). For 5 mg/kg XMU‐MP‐5 administered via intravenous (i.v.) injection, the area under the curve was 3,311.99 (ng/ml*h), the half‐life (T_1/2_) was 3.99 h, and the clearance (Cl) was 1.51 (l/h/kg), indicating a favorable pharmacokinetic profile.

A further toxicity evaluation of XMU‐MP‐5 was conducted in ICR mice. In this evaluation, i.v. administration of 60 mg/kg XMU‐MP‐5 q.d. did not lead to an increase in ALT or AST (Appendix Fig S5A and B). In addition, no body weight loss was observed (Appendix Fig S5C), and organs appeared normal on hematoxylin and eosin (H&E) staining (Appendix Fig S5D). These data indicate that XMU‐MP‐5 at a dosage of 60 mg/kg q.d. was well tolerated and possibly did not lead to significant toxicity in our study.

We next evaluated the *in vivo* antitumor efficacy of XMU‐MP‐5 in an H3122 xenograft mouse model. Tumor‐bearing mice were treated with 30 and 50 mg/kg XMU‐MP‐5 or 50 mg/kg crizotinib once daily for 11 days, and all treatments led to dose‐dependent tumor growth inhibition (Fig 2E and F). In the XMU‐MP‐5 treatment groups, the tumor growth inhibition rate ranged from 50 to 80% (Fig 2E). Both dosages were well tolerated, with no significant changes in body weight during the experiment (Appendix Fig S3D). After the last treatment, mice were sacrificed and dissected, and xenograft tumors were harvested for further analysis. Data obtained from H&E and immunohistochemical (IHC) staining indicated that XMU‐MP‐5 dose‐dependently inhibited ALK activity and induced tumor cell apoptosis *in vivo*, as indicated by the decreased ALK phosphorylation level and the induction of caspase‐3 cleavage, respectively (Fig 2G). These results demonstrate that XMU‐MP‐5 exhibits notable antitumor activity both *in vitro* and *in vivo*.

### Structural basis for the activity of XMU‐MP‐5 against ALK

**Figure 3:**
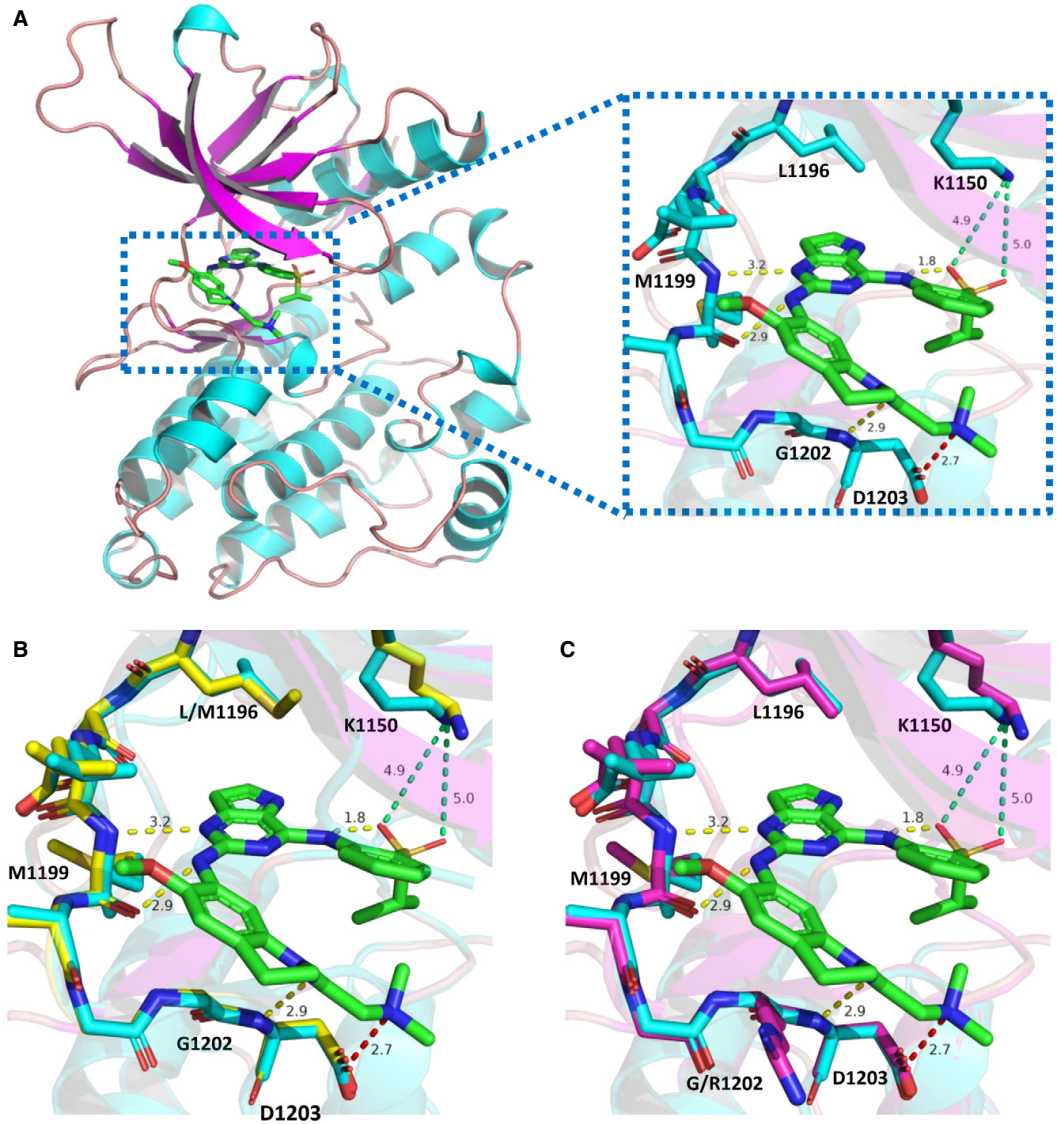
Structural basis for the inhibition of XMU‐MP‐5 against wild‐type and mutant ALK X‐ray cocrystal structure of XMU‐MP‐5 with wild‐type ALK (PDB ID: 7BTT). Specific interactions are depicted as dash lines, hydrogen bonds are shown in yellow and salt bridge is colored red.Structure analysis of XMU‐MP‐5 with ALK(L1196M) mutant (PDB ID: 2YFX) by molecular docking. Carbon chains of L1196M (yellow) model were overlaid on wild‐type ALK (blue).Comparison of XMU‐MP‐5 bound with wild‐type ALK (blue) and G1202R (purple). ALK(G1202R) homology model was developed based on the published X‐ray structure of ALK(L1196M) (PDB ID: 2YFX). Source data are available online for this figure.

To better understand the high potency of XMU‐MP‐5 against EML4‐ALK, we determined the X‐ray cocrystal structure of this compound bound to the ALK kinase domain (PDB ID: 7BTT) (Appendix Table S4). The structure of the complex revealed that XMU‐MP‐5 forms favorable interactions within the ATP‐binding pocket of the ALK kinase domain (Fig 3A). The pyrimidine and pyrimidin‐2‐amine moieties of XMU‐MP‐5 form hydrogen bonds with the main chain amide and carbonyl groups of M1199 in ALK, respectively, acting to tether the compound to the hinge region of the kinase. The pyrrolo‐pyrimidine core, along with the terminal isopropyl moiety of the compound, wraps the hydrophobic side chain of L1196 and forms favorable hydrophobic interactions with it, while the sulfuryl group fits well in the polar environment adjacent to the side chain of K1150 in ALK. At the other end of the compound, the carbonyl group of the drug also forms a hydrogen bond with the main chain amide group of D1203. Importantly, the hydrophilic tail of XMU‐MP‐5 is positively charged and forms a salt bridge with the negatively charged side chain carboxyl group of D1203, contributing to the high affinity and selectivity of XMU‐MP‐5 for ALK. This structure is not often seen in kinase inhibitors and thus represents a unique feature of this scaffold (Kong *et al*, 2019).

Secondary mutations in the ALK kinase domain may lead to resistance to ALK TKIs (Katayama *et al*, 2012; Ou *et al*, 2014; Gainor *et al*, 2016). For example, the gatekeeper mutation L1196M is often observed in crizotinib‐resistant cases (Toyokawa & Seto, 2014). We modeled the L1196M mutation in the wild‐type ALK kinase domain to discover the impact of this gatekeeper mutation on XMU‐MP‐5 binding. Our model revealed that this mutation had little effect on the main structure of the ALK kinase domain, and the methionine residue neither contacted the pyrrole moiety of XMU‐MP‐5 directly nor induced polar repulsion of the compound (Fig 3B and Appendix Fig S6A). We next modified the L1196M crystal structure *in silico* to simulate the G1202R mutation, which is the most common mutation acquired during treatment with second‐generation ALK TKIs (Katayama *et al*, 2012; Ou *et al*, 2014; Gainor *et al*, 2016). The replacement of glycine with arginine introduces a large, charged side chain to impair the binding of some drugs, such as crizotinib (Fig 3C and Appendix Fig S6B) (Sakamoto *et al*, 2011; Friboulet *et al*, 2014; Kodama *et al*, 2014; Zhang *et al*, 2016a, b). However, the indoline moiety of XMU‐MP‐5 was juxtaposed with the alkyl moiety of the R1202 side chain, thus not only avoiding steric hindrance but also producing favorable van der Waals interactions between these moieties. More importantly, none of these mutations disrupted the interactions between XMU‐MP‐5 and the ALK kinase domain. Therefore, XMU‐MP‐5 was predicted to be able to overcome the L1196M and G1202R drug resistance mutations in ALK, and this hypothesis was confirmed by the following data.

### XMU‐MP‐5 inhibits ALK secondary resistance mutations including G1202R

**Figure 4:**
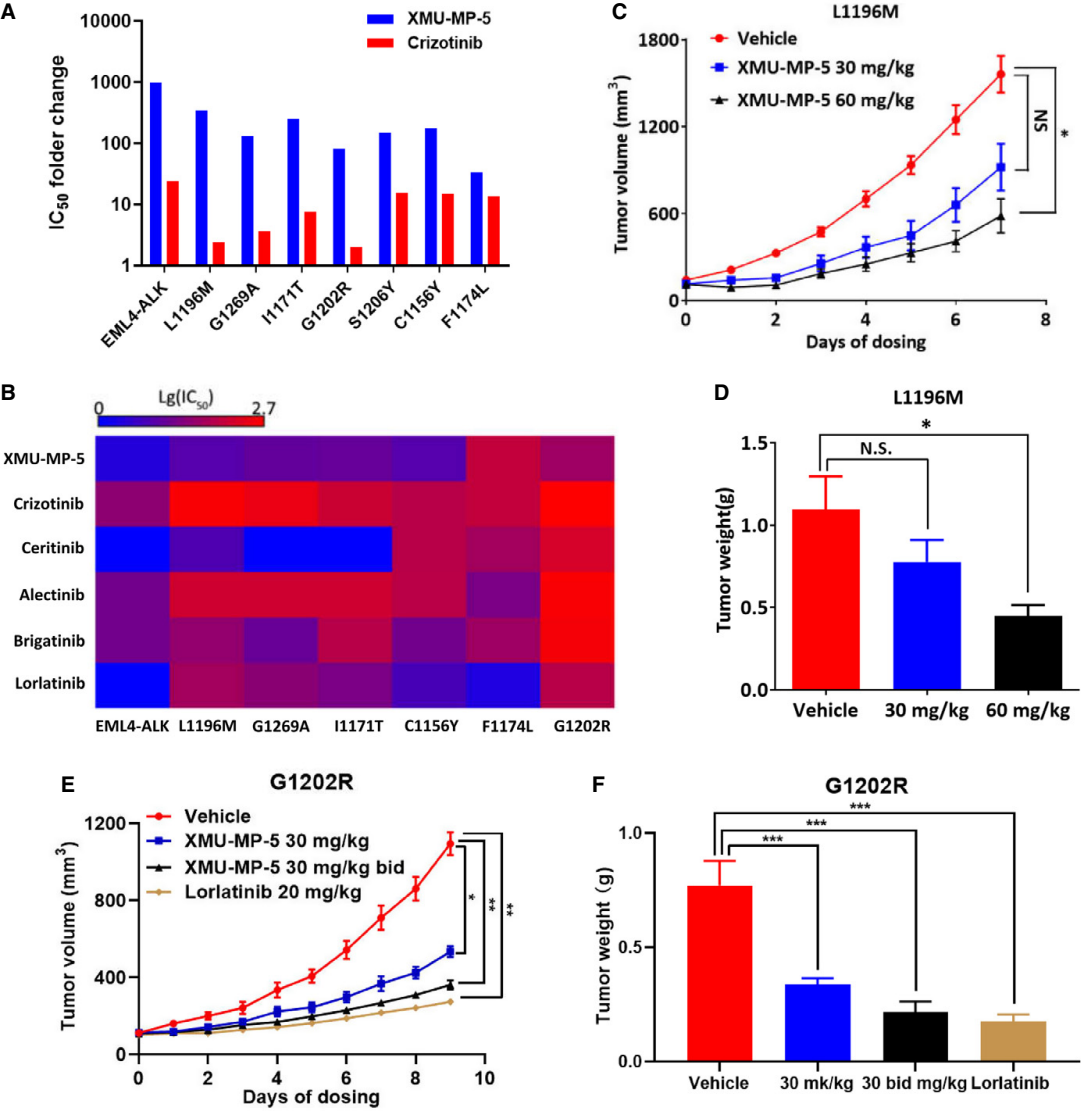
XMU‐MP‐5 overcomes secondary mutations on ALK ACell viability was measured by MTS assay. Each concentration was performed in triplicate. The exact IC_50_ value was presented as Mean ± SEM from three independent experiments (*n* = 3) and shown in Appendix Table S6. IC_50_ fold changes of wild type and mutant EML4‐ALK Ba/F3 cell lines to parental Ba/F3 cell line of XMU‐MP‐5 and crizotinib were determined.BHeatmap of the anti‐proliferative activity of ALK inhibitors across Ba/F3 cell lines with different ALK mutations.C, D
*In vivo* activity of XMU‐MP‐5 against L1196M xenograft mouse model. BALB/c nude mice bearing L1196M Ba/F3 xenograft tumors were treated with vehicle, 30 or 60 mg/kg XMU‐MP‐5 by tail vein injection once daily. Tumor volume (C) and tumor weight (D) were shown as mean ± SEM (*n* = 6). Statistical comparisons were performed using a two‐tailed, unpaired Student’s *t* test for (C), N.S., *P* = 0.1482; *P = 0.037. Statistical comparisons were performed using a one‐way ANOVA with Dunnett’s test for (D), N.S., *P* = 0.2162; *P = 0.0122.E, FEfficacy of XMU‐MP‐5 in G1202R Ba/F3 xenograft mouse model. BALB/c nude mice bearing G1202R Ba/F3 cells xenograft tumors were administrated with vehicle or XMU‐MP‐5 by tail vein injection twice daily. Lorlatinib was used as a positive control by oral administration. Tumor volume (E) and tumor weight (F) were shown as mean ± SEM (*n* = 6). Statistical comparisons were performed using a two‐tailed, unpaired Student’s *t* test for (E), Vehicle vs XMU‐MP‐5 30 mg/kg, *P = 0.0433; Vehicle vs XMU‐MP‐5 30 mg/kg BID, **P = 0.0093; Vehicle vs Lorlatinib, **P = 0.0029. Statistical comparisons were performed using a one‐way ANOVA with Dunnett’s test for (F). Vehicle vs XMU‐MP‐5 30 mg/kg, ***P = 0.0008; Vehicle vs XMU‐MP‐5 30 mg/kg BID, ***P = 0.0003; Vehicle vs Lorlatinib, ***P = 0.0002.

To investigate the activity of XMU‐MP‐5 against ALK resistance mutants, we generated Ba/F3 cell lines with ALK mutations that have been frequently found in patients with clinical secondary resistance to ALK TKIs; these mutations included L1196M (gatekeeper mutation), G1269A, I1171T, S1206Y, C1156Y, F1174L, and G1202R (solvent front mutation) (Kong *et al*, 2019). Cell viability data revealed that XMU‐MP‐5 was active against all of these crizotinib‐resistant mutations except F1174L (Fig 4A, and Appendix Tables S5 and S6). XMU‐MP‐5 displayed high potency against L1196M and G1269A, the most common mutations occurring in crizotinib‐refractory tumors, with IC_50_ values of 12 nM and 30.9 nM, respectively (Appendix Tables S5 and S6). XMU‐MP‐5 was also effective against the alectinib resistance mutation I1171T or the ceritinib resistance mutation C1156Y (Fig 4B and Appendix Table S5). Crizotinib, ceritinib, brigatinib, and alectinib exhibited high IC_50_ values ranging from 206 nM to 555 nM in ALK(G1202R) Ba/F3 cells (Appendix Table S5), consistent with the clinical observation that G1202R confers a high level of resistance to all first‐ and second‐generation ALK TKIs (Katayama *et al*, 2012; Ou *et al*, 2014; Gainor *et al*, 2016). Lorlatinib was reported to have activity against cells harboring the G1202R mutation (Zou *et al*, 2015), and its IC_50_ in ALK(G1202R) Ba/F3 cells was 90 nM in our study. We also found that XMU‐MP‐5 may have potency to overcome this mutation, as it had an IC_50_ value of 49 nM in this cell line (Appendix Tables S5 and S6). In addition, XMU‐MP‐5 dose‐dependently induced apoptosis and suppressed the ALK signaling pathway (Appendix Figs S7 and S8), abilities that correlated well with its antiproliferative activities.

We next evaluated the *in vivo* activity of XMU‐MP‐5 in ALK(L1196M) and ALK(G1202R) Ba/F3 xenograft mouse models. Administration of 60 mg/kg XMU‐MP‐5 for 7 days resulted in significant tumor growth inhibition in the L1196M xenograft mouse model, as exemplified by the tumor volumes and tumor weights (Fig 4C and D). Further H&E and IHC staining indicated that XMU‐MP‐5 induced apoptosis by inhibiting ALK phosphorylation *in vivo* (Appendix Fig S9A). As XMU‐MP‐5 showed activity to inhibit ALK(G1202R) Ba/F3 cell growth in a viability assay, we also established a xenograft mouse model to determine whether it had *in vivo* activity against the G1202R mutant. As lorlatinib showed promising activity against the G1202R mutant in a mouse model, one of its most important advantages compared to second‐generation inhibitors, we also compared XMU‐MP‐5 with lorlatinib in this study. Treatment with 30 mg/kg XMU‐MP‐5 twice daily by i.v. injection led to significant inhibition of tumor growth that was similar to the effect of 20 mg/kg lorlatinib administered per oral (p.o.) (Fig 4E and F). In both xenograft mouse models, XMU‐MP‐5 was well tolerated without body weight loss or gross signs of toxicity (Appendix Fig S9B and C). Together, these results indicate that XMU‐MP‐5 exhibits potent efficacy against clinically relevant secondary ALK mutations, including G1202R, both *in vitro* and *in vivo*.

### XMU‐MP‐5 induces tumor regression in genetically engineered mouse models

**Figure 5:**
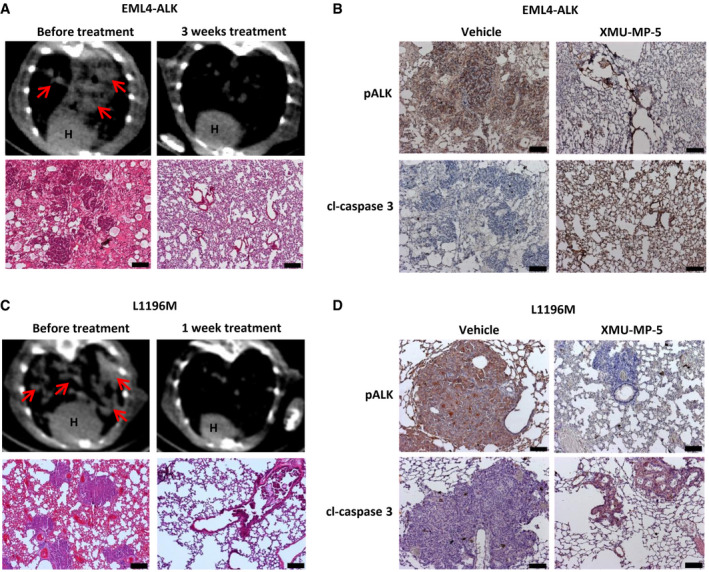
XMU‐MP‐5 treatment leads to tumor regression in GEM models Representative CT images (upper panel) and H&E staining (lower panel) of lungs from EML4‐ALK tumor‐bearing mice are shown. The complete set of CT images refers to Appendix Figure S10A. Mice were treated with 30 mg/kg XMU‐MP‐5 twice daily for 3 weeks. H: heart; arrow indicated tumor. Scale bars, 200 μm.IHC analyses of lungs from EML4‐ALK transgenic mice. pALK and Cl‐caspase 3 were probed. Scale bars, 100 μm.Representative CT images (upper panel) and H&E staining (lower panel) of lungs from EML4‐ALK(L1196M) tumor‐bearing mice are shown. The complete set of CT images refers to Appendix Figure S10B. Mice were treated with 40 mg/kg XMU‐MP‐5 twice daily for 1 week. H: heart; arrow indicated tumor. Scale bars, 200 μm.IHC analyses of lungs from EML4‐ALK(L1196M) transgenic mice. pALK and Cl‐caspase 3 were probed. Scale bars, 100 μm.

As xenograft mouse models cannot fully mimic the complex microenvironment of lung cancer initiation and development *in vivo*, we next established a GEM model with lung‐specific doxycycline‐inducible EML4‐ALK expression to further evaluate the activity of XMU‐MP‐5 *in vivo* (Soda *et al*, 2008; Chen *et al*, 2010, 2014). EML4‐ALK expression was induced in GEM mice (*n* = 3) by daily doxycycline feeding, tumors developed *in situ*, and lung cancer progression was monitored by computed tomography (CT). Tumor masses developed rapidly in this model, while administration of 30 mg/kg XMU‐MP‐5 twice daily for one week resulted in significant tumor regression; moreover, complete remission with no discernible tumor regrowth was achieved after three weeks of treatment (Fig 5A and Appendix Fig S10A). Consistent with these results, histological examination indicated significant resolution of tumor nodules. IHC staining of the lungs after treatment demonstrated that XMU‐MP‐5 markedly decreased the ALK phosphorylation level, which was associated with a significant increase in the activated caspase 3 level (Fig 5B). As expected, in another GEM model expressing the EML4‐ALK fusion oncogene with the L1196M mutation (*n* = 3) (Chen *et al*, 2014), substantial tumor regression was also observed within one week of treatment with 40 mg/kg XMU‐MP‐5 twice daily (Fig 5C and Appendix Fig S10B). Consistent with the CT data, H&E analysis of the lungs also indicated that one week of treatment with XMU‐MP‐5 led to complete resolution of the tumor nodules. IHC staining further showed that XMU‐MP‐5 induced tumor remission by inhibiting ALK activity and inducing tumor cell apoptosis (Fig 5D). In summary, the substantial efficacy of XMU‐MP‐5 in these GEM models suggests its potential clinical therapeutic efficacy.

## Discussion

Crizotinib has been successfully used in the clinic to treat ALK‐rearranged NSCLC, and patients always have a median progression‐free survival (PFS) time of 10.9 months after crizotinib treatment (Gadgeel, 2017). Unfortunately, patients inevitably experience relapse after crizotinib treatment. Many crizotinib resistance mechanisms have been discovered in preclinical and clinical studies; among them, secondary mutations account for 20–30% of cases of post‐crizotinib resistance (Lin *et al*, 2017). Second‐generation ALK inhibitors have been successively approved by the FDA with the goal of overcoming crizotinib resistance mutations, but some clinical limitations remain.

Our studies have identified a novel potent ALK inhibitor, XMU‐MP‐5, that was effective against both wild‐type and mutated ALK *in vitro* and *in vivo*. XMU‐MP‐5 was highly potent against Ba/F3 and H3122 cells expressing EML4‐ALK, with IC_50_ values of 4.2 and 5.7 nM, respectively. XMU‐MP‐5 was also potent against Ba/F3 cells transformed with many different ALK mutations except F1174L. L1196M, functionally similar to T790M in EGFR and T315I in BCR‐ABL, is the gatekeeper mutation located in the hinge region of the kinase ATP pocket and hinders TKI binding. G1269A is another mutation located in the ATP‐binding pocket. These two mutations are the most common mutations found in crizotinib‐resistant tumors (Lin *et al*, 2017). XMU‐MP‐5 inhibited the proliferation of cells harboring these two mutations *in vitro* and significantly suppressed tumor growth in the L1196M xenograft mouse model *in vivo*. XMU‐MP‐5 also displayed potency to overcome the alectinib resistance mutation I1171T and ceritinib resistance mutation C1156Y. G1202R is considered the most refractory ALK mutation that occurs in the clinic and commonly arises after treatment with second‐generation ALK inhibitors. G1202R, a solvent front mutation, confers resistance by impairing the binding of TKIs to ALK through steric hindrance (Lin *et al*, 2017). Consistent with previous reports, crizotinib, alectinib, brigatinib, and ceritinib were inactive against G1202R in our viability experiments. Lorlatinib was developed to overcome the G1202R mutation and was found to exhibit significant activity against G1202R both *in vitro* and *in vivo* (Zou *et al*, 2015); therefore, lorlatinib is considered a third‐generation ALK inhibitor. In addition, it was approved by the FDA in 2018. Although lorlatinib has shown promising activity in preclinical studies and clinical trials, there is still a need to develop more third‐generation ALK TKIs for clinical use, especially to target the G1202R mutation. XMU‐MP‐5 showed substantial activity against G1202R *in vitro*, with an IC_50_ value of 49 nM, and it significantly inhibited ALK(G1202R) Ba/F3 tumor growth in the xenograft mouse model. Structural analysis also provided a structural basis for the potency of XMU‐MP‐5 against EML4‐ALK and acquired resistance mutations in ALK. XMU‐MP‐5 has a unique structure that differs from those of other ALK inhibitors, and it binds with ALK stably by forming favorable interactions, such as salt bridges. More importantly, it can be juxtaposed with the large, charged side chain introduced by the G1202R mutation. These data suggest that XMU‐MP‐5 might be a novel therapeutic agent to overcome clinically relevant secondary ALK mutations, including the highly variable mutation G1202R.

To fully mimic the complex microenvironment of tumor initiation and development *in vivo*, GEM models have been developed and used to better evaluate the *in vivo* activity of TKIs (Politi *et al*, 2010; McFadden *et al*, 2016). In contrast to xenograft models, tumors in GEM models develop *in situ* and normally interact with all immune, angiogenesis, and inflammatory processes (Politi & Pao, 2011; Hayes *et al*, 2014). Although GEM models for EML4‐ALK lung cancer were first established in 2008 (Soda *et al*, 2008), the use of GEM models to assess the *in vivo* potency of ALK TKIs was limited due to technical challenges (Chen *et al*, 2010, 2014). Here, we developed a GEM model specifically for EML4‐ALK lung cancer and found that XMU‐MP‐5 led to strong tumor regression resulting from ALK activity inhibition and tumor cell apoptosis. We also developed another GEM model harboring EML4‐ALK(L1196M) lung tumors (Chen *et al*, 2014), and treatment with XMU‐MP‐5 led to obvious shrinkage of tumors in this model. These results further confirmed the potent *in vivo* anticancer activity of XMU‐MP‐5 against both the EML4‐ALK and L1196M mutations.

Poor kinase selectivity of a TKI might result in a narrow therapeutic window and limit its clinical usage (Sakamoto *et al*, 2011; Friboulet *et al*, 2014; Kodama *et al*, 2014; Zhang *et al*, 2016a, b). Existing ALK inhibitors exhibit potency against multiple kinases. For example, crizotinib was initially designed as a MET inhibitor, and crizotinib, ceritinib and lorlatinib have been shown to have binding affinity for ROS1 (Drilon *et al*, 2018). XMU‐MP‐5 showed minimal off‐target kinase activity in a KINOMEscan assay and was much more selective than the first‐ and second‐generation ALK TKIs (Huber *et al*, 2014; Sakamoto *et al*, 2011; Zhang *et al*, 2016a, b; Jang *et al*, 2017). XMU‐MP‐5 was inactive against MET and was less sensitive than other TKIs to ROS1. XMU‐MP‐5 showed superior activity against EML4‐ALK Ba/F3 cells compared with wild‐type Ba/F3 cells, with an IC_50_ ratio of 1,553. Furthermore, XMU‐MP‐5 showed an outstanding effect on the ALK‐positive lung cancer cell line H3122 but negligible activity against the normal lung cell line MRC5, with an IC_50_ ratio of 803. More importantly, XMU‐MP‐5 showed no effect on human PBMCs. These data indicated that XMU‐MP‐5 might have a wide therapeutic window. In addition, treatment with XMU‐MP‐5 did not lead to a significant change in body weight in our *in vivo* mouse models. With high selectivity and safety, XMU‐MP‐5 can be used as a good start point for the new‐generation ALK inhibitors to overcome acquired resistance during the clinical treatment of EML4‐ALK‐positive NSCLC.

## Materials and Methods

### Chemical synthesis of XMU‐MP‐5

XMU‐MP‐5 was synthesized using the reported procedure in patent application US 10,508,118 B2 (Appendix Fig S11). ^1^H NMR (600 MHz, DMSO‐*d*_6_) δ 11.39 (s, 1H), 9.00 (s, 1H), 8.87 (s, 1H), 8.09 (d, *J* = 8.2 Hz, 1H), 7.83 (d, *J* = 7.9 Hz, 1H), 7.64 (t, *J* = 7.8 Hz, 1H), 7.53 (s, 1H), 7.34 (t, *J* = 7.7 Hz, 1H), 7.17 (s, 1H), 6.89 (s, 1H), 6.23 (s, 1H), 4.15 (t, *J* = 8.3 Hz, 2H), 3.78 (s, 3H), 3.39–3.33 (m, 1H), 3.17 (s, 2H), 3.07 (t, *J* = 8.4 Hz, 2H), 2.26 (s, 6H), 1.13 (d, *J* = 6.8 Hz, 6H). ^13^C NMR (150 MHz, DMSO‐*d*_6_) δ 166.72, 153.83, 150.59, 146.37, 144.73, 138.57, 136.13, 134.46, 130.56, 129.99, 128.31, 126.33, 126.23, 123.64, 123.33, 110.71, 108.51, 107.17, 100.69, 62.79, 55.92, 54.54, 47.23, 45.25, 27.71, 14.83. HR‐MS (ESI): C_28_H_34_N_7_O_4_S^+^, calculated 564.2387, found 564.2378.

### Cell lines and culture

The human NSCLC cell lines NCI‐H3122 and PC9 were obtained from American Type Culture Collection (Manassas, VA, USA). A549 and NCI‐H1299 were provided by Cell Bank, Chinese Academy of Sciences (Shanghai, China). MRC5 was purchased from Cellcook inc. (Guangzhou, China). The murine Pro‐B cell line Ba/F3 cells were provided by Jianming Zhang from Shanghai Jiaotong University. All cells were authenticated by short tandem repeat testing and tested negative for mycoplasma contamination using Luminescent Mycoplasma Detection Kit (Beyotime, Shanghai, China). H3122 was grown in RPMI 1640 medium containing 10% fetal bovine serum, other NSCLC cell lines including A549, H1299, and PC9 were cultured in DMEM medium containing 10% fetal bovine serum. Human PBMC cells were cultured in RPMI 1640 medium containing 10% fetal bovine serum, and normal lung cell line MRC5 was grown in MEM medium containing 10% fetal bovine serum. The murine Pro‐B cells Ba/F3 were grown in RPMI 1640 medium containing 10% FBS and 10% conditioned medium of IL‐3. To generate Ba/F3 cell lines expressing EML4‐ALK and ALK mutants of L1196M, C1156Y, G1269A, I1171T, G1202R, F1174L, and S1206Y, plasmids and vector pBABE‐puro were co‐transfected with 293T cells for 2 days, then the supernatants were used to transduce parental Ba/F3 cells (Zhang *et al*, 2010). These engineered Ba/F3 cells were cultured in RPMI 1640 medium containing 10% FBS but without IL‐3. All of these cell lines were maintained at 37°C and 5% CO_2_.

### Cell survival assay

Cells were seeded into 96‐well plates at a density of 5,000 cells/well (NSCLC cell lines), 20,000 cells/well (Ba/F3 cell lines and human PBMC) or 3,000 cells/well (MRC5) then the serial dilutions of XMU‐MP‐5 and other ALK TKIs were added. NSCLC cell lines were cultured in the presence of drugs for 72 h while Ba/F3 cell lines were treated for 48 h. Cell viability was determined by MTS assay (Promega).

### Colony formation assay

H3122 cells were seeded into 6‐well plates at a density of 10,000 cells/well, then treated with XMU‐MP‐5 or crizotinib at indicated concentrations for 18 days. The fresh medium with indicated drugs was added every 3 days. After experiment, cells were fixed by 4% paraformaldehyde and stained by crystal violet (Cat# C0121, Beyotime, Shanghai, China) for 1 min and then washed with PBS twice.

### Western blot

Cells were treated with XMU‐MP‐5 and crizotinib at indicated doses for 4 h, then washed on ice with cold PBS before the addition of lysis buffer (3 M NaCl, 1 M Tris‐HCl, pH 7.5, 1% Triton X‐100, 5% glycerol) supplemented with protease inhibitor 50 mM NaF, 1 mM Na_3_VO_4_, 5 μg/ml Leupeptin, and 1mM PMSF. After scraping, cell lysates were sonicated then centrifuged 12,000 *g* for 30 min at 4°C. The protein concentrations were measured by BCA protein assay kit (Cat# P0012S, Beyotime, Shanghai, China). Antibodies of phospho‐ALK (Y1604) (Cat# 3341,1:1,000 dilution), ALK (Cat# 3633P, 1:1,000 dilution), phospho‐STAT3 (Y705) (Cat #9131, 1:1,000 dilution), STAT3 (Cat# 9139, 1:1,000 dilution), phospho‐AKT (S473) (Cat# 4060S, 1:1,000 dilution), AKT (Cat# 4691S, 1:1,000 dilution), phospho‐ERK1/2 (T202/Y204) (Cat# 9101L, 1:1,000 dilution), and ERK1/2 (Cat# 9102, 1:1,000 dilution) were purchased from Cell Signaling Technology. β‐actin (Cat# A5316, 1:5,000 dilution) was purchased from Sigma Aldrich.

### KINOMEscan profiling of XMU‐MP‐5

XMU‐MP‐5 was profiled using KINOMEscan technology, an active‐site‐dependent competition‐binding assay (Eurofins DiscoverX Corporation, San Diego, CA). The KINOMEscan selectivity score (S) is a quantitative measure of a compounds’ selectivity (Karaman *et al*, 2008; Anastassiadis *et al*, 2011). It is calculated by dividing the number of kinases that bind to the compound by the total number of kinases tested. The results are reported as “control%” (ctrl%) in which lower numbers a represent higher affinity binding; ctrl% = (test compound signal − positive control signal)/(negative control signal − positive control signal) × 100, where the negative control = DMSO (ctrl% = 100%), and the positive control = control compound (ctrl% = 0%); selectivity score S(10) = (number of kinases with ctrl% ≤ 10%)/(number of kinases tested). The ctrl% of < 10% means very strong inhibition and the ctrl% of > 70% means very weak inhibition. Kinome illustration reproduced courtesy of Cell Signaling Technology (www.cellsignal.com). The kinase group or individual kinase names in current study includes TK (tyrosine kinase); TKL (TK‐like); STE (homologs of yeast sterile 7, sterile 11, sterile 20 kinases); AGC (kinases PKA, PKG, PKC families); CAMK (calcium/calmodulin‐dependent protein kinase); CK1 (casein kinase 1); CMGC (kinases CDK, MAPK, GSK3, CLK families).

### Protein expression, crystallization, and structure determination

Construct spanning kinase catalytic domain residues 1,093–1,411 of the human ALK with a S1281G mutant was expressed and purified using a Baculovirus/insect cell system as described (Lee *et al*, 2010). Crystals used in this study were prepared by hanging drop vapor diffusion. The ALK/XMU‐MP‐5 complex crystals were prepared by co‐crystallization. The compound was added to 14.0 mg/ml ALK proteins to a final concentration of 2 mM two hours before setting up the crystallization tray. The reservoir solution for growing ALK/XMU‐MP‐5 crystals was 0.2 M Sodium chloride, 25% w/v Polyethylene glycol 3,350, 0.1 M Tris pH 8.5. Diffraction data was collected on beamline BL19U1 at Shanghai Synchrotron Radiation Facility (SSRF). The diffraction data were processed using HKL3000 (Minor *et al*, 2006). The structure was solved by molecular replacement with Phaser using the previously determined ALK S1281G structure (PDB ID 3L9P) as the search model (McCoy *et al*, 2007; Lee *et al*, 2010). Repeated rounds of manual refitting and crystallographic refinement were then performed using COOT and Phenix (Emsley & Cowtan, 2004; Adams *et al*, 2010). The inhibitor was modeled into the closely fitting positive Fo‐Fc electron density and included in following refinement cycles. Topology and parameter files for the inhibitor were generated using eLBOW (Moriarty *et al*, 2009). The crystal diffraction data and refinement statistics were summarized in Table S3. The crystal structure has been deposited in Protein Data Bank (PDB) with the accession ID 7BTT.

### Autodock and molecular docking

The molecular docking procedure was according to the protocol within Autodock. The crystal structure of protein‐ligand complex of L1196M (PDB ID: 2YFX) was selected as the template and water molecules were deleted. XMU‐MP‐5 was docked into the crystal structure of L1196M by Autodock vina (Morris *et al*, 2009; Trott & Olson, 2009). The initial 3D conformation of compound was optimized in Avogadro 1.1.1 (Hanwell *et al*, 2012). XMU‐MP‐5 was docked into the binding site. The G1202R model was prepared by SWISS‐MODEL Server (Bienert *et al*, 2017; Liao *et al*, 2017; Waterhouse *et al*, 2018). Molecular graphics was prepared by PyMOL.

### Xenograft mouse models

BALB/c nu/nu mice (half male and half female) were purchased from Charles River (Beijing, China). All animal care and experimental procedures complied with the guidelines from the Institutional Animal Care and Use Committee at Experimental Animal Centre at Xiamen University. In brief, animals were kept in Individual Ventilation Cages at constant temperature of 26°C and humidity of 40–70% with maximum 5 animals in each cage. During the entire study period, the mice had free access to irradiation‐sterilized dry granule food and sterile drinking water. H3122, L1196M‐Ba/F3, and G1202R‐Ba/F3 cell lines were used to evaluate the *in vivo* efficacy of XMU‐MP‐5. BALB/c nu/nu mice at the age of 4–6 weeks were subcutaneously injected with 1 × 10^7^ cells. Once the tumors reach 100 to 200 mm^3^, mice were treated with XMU‐MP‐5 or vehicle at indicated dosage by tail vein injection. Tumor volume and body weight were measured every day. Tumor volume of each mouse was calculated as V = length × width^2^/2. Tumor growth inhibition (TGI) of each treatment group was calculated as TGI = [1 − (T−T_0_)/(V−V_0_)]^3^ × 100%. T (treatment group) and V (vehicle group) are the mean tumor volumes on the final dosing day, respectively, and T_0_ and V_0_ are the mean tumor volumes on the first dosing day.

### Genetically engineered mouse models

5’mROSA‐CAG‐lox‐stop‐lox‐EML4‐ALK L1196M‐3′mROSA (designated RC L1196M for Rosa‐Conditional‐EML4‐ALK L1196M) gene fragment was constructed by PCR assembling. sgRNA‐targeting ROSA26 site (sgROSA) was purified from *in vitro* transcribing the PCR product amplified from plasmid pX330‐sgRosa26‐1‐T2A‐BFP (Addgene #64216) with the forward primer T7‐sgRosa26‐for (5′‐TTAATACGACTCACTATAGGACTCCAGTCTTTCTAGAAGAGT‐3′) and the reverse primer T7‐sgRNA‐rev (5′‐AAAAGCACCGACTCGGTGCC‐3′). RC L1196M gene fragment was injected in mouse embryos with mRNA encoding CAS9 enzyme and sgROSA. Transgenic mouse harboring Cre activatable EML4‐ALK L1196M gene in ROSA26 site was identified by PCR (Forward primer, 5′‐GGACAGATAGCTGGCGTGGATA‐3′; Reverse primer, 5′‐TCCGGACACCTGGCCTTCAT‐3′). Expression of EML4‐ALK L1196M gene in the lung epithelial compartment was achieved through nasal instillation of recombinant lentivirus expressing Cre. TetO‐EML4‐ALK/CC10rtTA bitransgenic was kindly gifted by Dr. Kwok‐Kin Wong in New York University Langone Medical Center. EML4‐ALK expression in lung epithelial compartment was achieved through feeding mice with Doxycycline‐containing diet (Research Diets, C11300‐2000). All mice were housed in a pathogen‐free environment in Jinan University. All experimental protocols were approved by the Institutional Committee for Animal Care and Use at Jinan University. All animal work was performed in strict accordance with the approved protocol.

### Immunohistochemistry

The xenograft tumors were fixed in formalin and paraffin‐embedded, then sectioned into 5 μm. Deparaffinization, rehydration, and antigen retrieval of these sections were then conducted in a row. After incubation of 3% H_2_O_2_ for 10 min, the sections were blocked by 5% BSA in 37°C for 20 min, and then incubated by indicated primary antibody (1:100 for the p‐ALK antibody and 1:500 for the cl‐Caspase3 antibody) in 4°C overnight. Then the sections were incubated by secondary antibody in 37°C for 20 min, followed by color reaction of DAB and counterstained with hematoxylin. At last, these sections were dehydrated and cleared, then mounted with neutral plastic.

### Statistical analysis

All data were analyzed using a two‐tailed *t* test or ANOVA, *P*‐values of < 0.05 were considered significant. Cell viability assays were repeated independently in triplicate, a two‐tailed Student’s *t* test was used to determine the statistical significance of difference between XMU‐MP‐5 and crizotinib in different cell lines. For *in vivo* analysis, *P*‐values of tumor volume between vehicle group and treatment groups were determined by ANOVA. *P*‐values of tumor weights between vehicle group and each treatment group were determined by a two‐tailed Student’s *t* test.

## Author contributions

YL, ZF, SJZ, XXH, and ZZ contributed equally to this work. JZ, CHY, LC, and XD conceived and designed the project. YL, ZF, SJZ, XXH, ZZ, YZL, ZD, LG, TZ, XS, WH, JZ, YL, BZ, JJ, FG, and ZW performed the experiments. YL, X XH, XHH, LL, QL, XH, SS, QW, LFC, DW, JZ, CHY, LC, and XD analyzed the data. YL, LL, JZ, CHY, LC, and XD wrote the manuscript. All authors read, edited, and approved the final manuscript.

## Conflict of interest

X. Huang is an employee of Hongyun Biotech Co., Ltd. The other authors declare that they have no conflict of interest.

## Supporting information



Appendix

Source Data for Appendix

Source Data for Figure 1

Source Data for Figure 2

Source Data for Figure 3

## Data Availability

The crystal structure has been deposited in Protein Data Bank (PDB) with the accession ID 7BTT (https://www.rcsb.org/structure/7BTT).
