# Supplementary material for: A new ALK inhibitor overcomes resistance to first‐ and second‐generation inhibitors in NSCLC
Source: EMBO Mol Med. 2021 Nov 30;14(1):e14296. doi: 10.15252/emmm.202114296 (PMC8749467; doi:10.15252/emmm.202114296)
Supplement: Supplementary file 1 — Appendix [file EMMM-14-e14296-s001.pdf]

Appendix

A new ALK inhibitor overcomes resistance to first- and second-generation inhibitors in NSCLC

Yue Lu[1†], Zhenzhen Fan[2†], Su-Jie Zhu[3,4†], Xiaoxing Huang[1†], Zhongji Zhuang[1†], Yunzhan Li[1], Zhou Deng[1], Lei Gao[2], Xuehui Hong[5], Ting Zhang[1], Li Li[1], Xihuan Sun[1], Wei Huang[1], Jingfang Zhang[1], Yan Liu[1], Baoding Zhang[1], Jie Jiang[1], Fu Gui[1], Zheng Wang[1], Qiyuan Li[6], Siyang Song[1], Xin Huang[7], Qiao Wu[1], Lanfen Chen[1], Dawang Zhou[1], Jianming Zhang[8*], Cai-Hong Yun[3**], Liang Chen[2***], Xianming Deng[1****]

Contents

Appendix Figure S1. Chemical structures of FDA-approved ALK inhibitors with different scaffolds.

Appendix Figure S2. XMU-MP-5 selectively induced cell apoptosis.

Appendix Figure S3. Antiproliferative activity of XMU-MP-5 against non-small cell lung cancer cell lines.

Appendix Figure S4. XMU-MP-5 has high selectivity over nontumorigenic cell lines.

Appendix Figure S5. Toxicity evaluation of XMU-MP-5 by ICR mice.

Appendix Figure S6. Molecular docking of XMU-MP-5 with ALK mutant kinases.

Appendix Figure S7. Cellular apoptosis analysis of XMU-MP-5 against ALK mutants transformed Ba/F3 cell lines.

Appendix Figure S8. Effect of XMU-MP-5 on ALK signaling pathways in Ba/F3 cell lines harboring ALK mutations.

Appendix Figure S9. *In vivo* efficacy of XMU-MP-5 in xenograft mouse models with ALK mutations.

Appendix Figure S10. *In vivo* efficacy of XMU-MP-5 in different GEM models.

Appendix Figure S11. Synthesis of XMU-MP-5.

Appendix Table S1 Full list of KINOMEscan profiling data of XMU-MP-5.

Appendix Table S2 S score of XMU-MP-5 and approved ALK TKIs.

Appendix Table S3 Pharmacokinetic properties of XMU-MP-5 in mice.

Appendix Table S4 X-ray data collection and refinement statistics.

Appendix Table S5 IC_{50} values of XMU-MP-5 against WT and ALK mutant Ba/F3 cell lines.

Appendix Table S6 Fold change IC_{50}s of ALK mutant Ba/F3 cell lines to WT Ba/F3 cell lines.

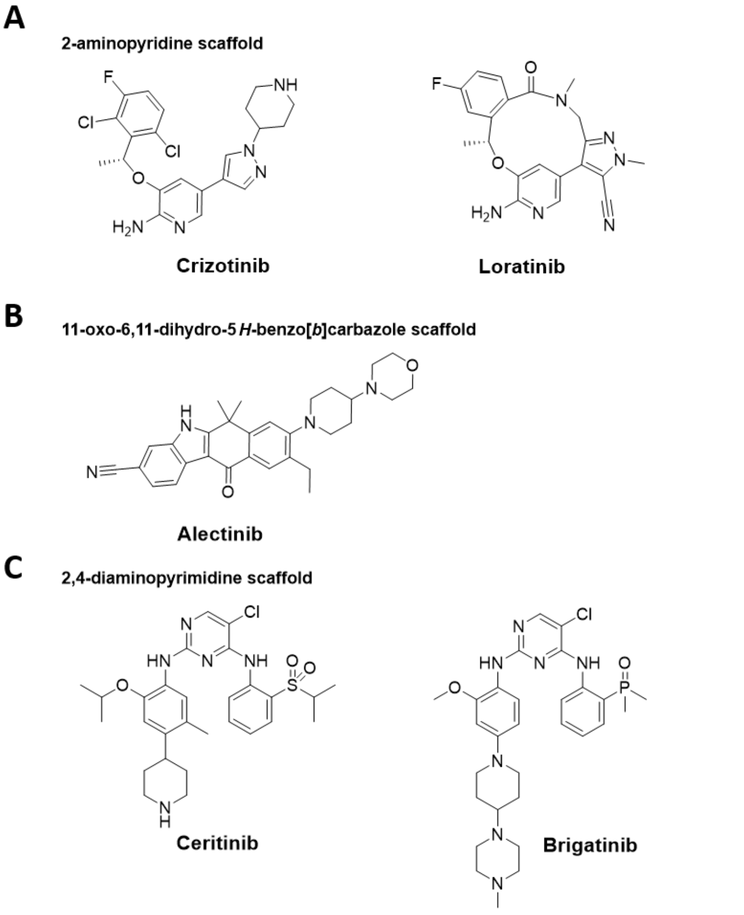

Appendix Figure S1. Chemical structures of FDA-approved ALK inhibitors with different scaffolds. The core structures of FDA-approved ALK inhibitors were classified into 3 different scaffolds.

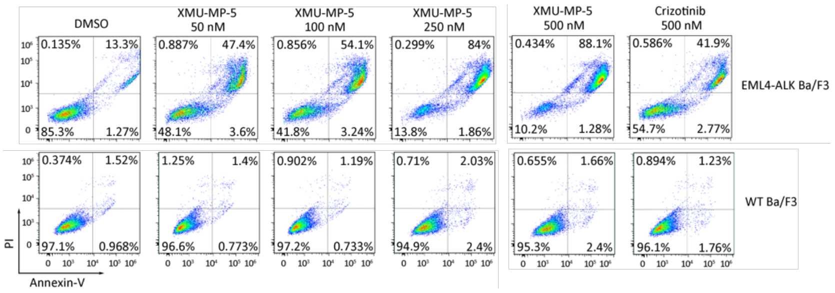

Appendix Figure S2 XMU-MP-5 selectively induced cell apoptosis.
EML4-ALK Ba/F3 and wild-type Ba/F3 cells were treated with XMU-MP-5 or crizotinib with the indicated concentrations for 24 hours. Apoptosis was detected by AnnexinV/PI staining.

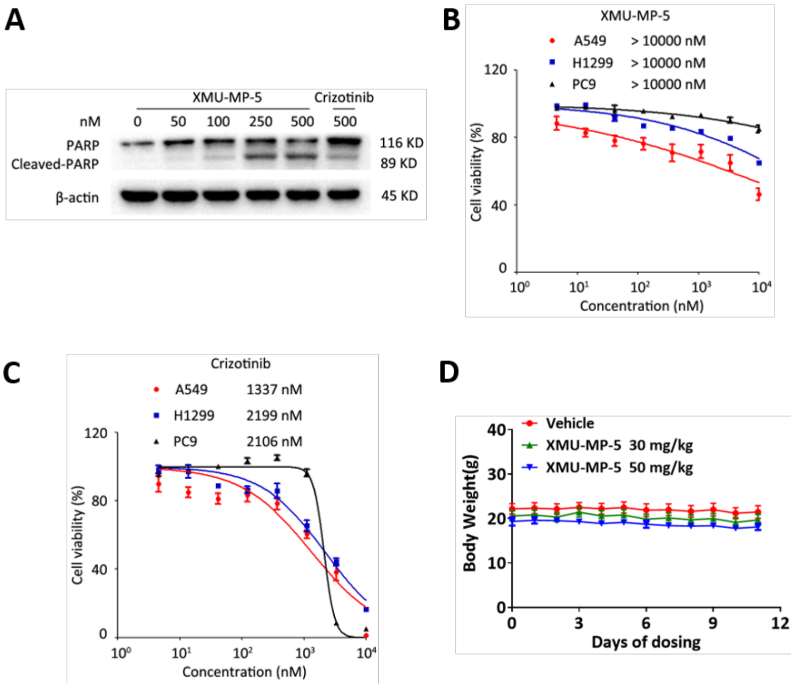

Appendix Figure S3 Antiproliferative activity of XMU-MP-5 against non-small cell lung cancer cell lines in vitro and in vivo.

A XMU-MP-5 induced cell apoptosis indicated by cleavage of PARP. H3122 cells were treated as indicated for 24 hours and then analyzed by immunobloting.

B, C XMU-MP-5 showed negligible antiproliferative activity against ALK negative lung cancer cell lines. Cells were treated with series concentrations of XMU-MP-5 for 48 h. Cell viability was detected by MTS assay. Crizotinib was used as reference.

D No Body weight change was observed after XMU-MP-5 treatment for 11 days. Data were shown as mean ± SD (n = 6).

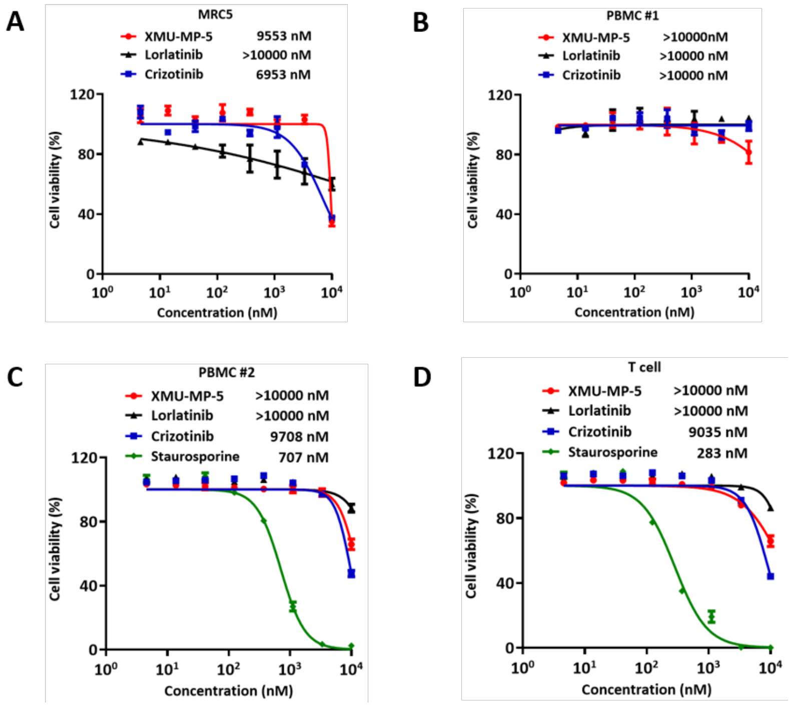

Appendix Figure S4 XMU-MP-5 has high selectivity over nontumorigenic cell lines.

A Anti-proliferative activities of XMU-MP-5 and crizotinib against human lung fibroblast cell line MRC-5

B, C Effect of XMU-MP-5 and crizotinib on human peripheral blood mononuclear cells (PBMC) that freshly isolated from 2 healthy donors.

D Activity of XMU-MP-5 and crizotinib on human T cells.

All of the cells were treated with increasing concentration of each drug for 48 hours and then analyzed by CellTiter-Glo. Each concentration point was performed in triplicate. Data were presented as Mean ± SEM from three indepedent experiments (n = 3) .

Formatted: English (US)

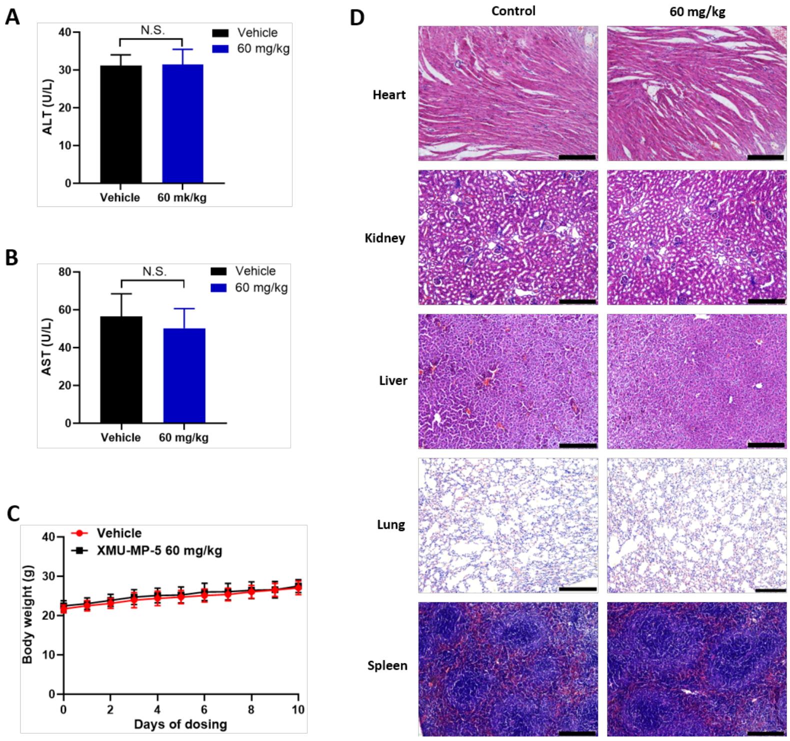

Appendix Figure S5 Toxicity evaluation of XMU-MP-5 by ICR mice.

A, B The activity of ALT and AST after 10 days' dosing were measured. Mice were administrated with vehicle and 60 mg/kg XMU-MP-5 by tail vein injection. ALT and AST data were shown as Mean ± SD (n=7). Statistical comparasions were performed using an unpaired Student's *t* test for (A) and (B). (A) N.S., P = 0.9091; (B) N.S., P = 0.36.

C Body weights of each group were measured every day. Data were shown as mean ± SD (n = 7).

D Representative images of heart, kidney, liver, lung and spleen analyzed by H&E. Scale bars, 250 μm.

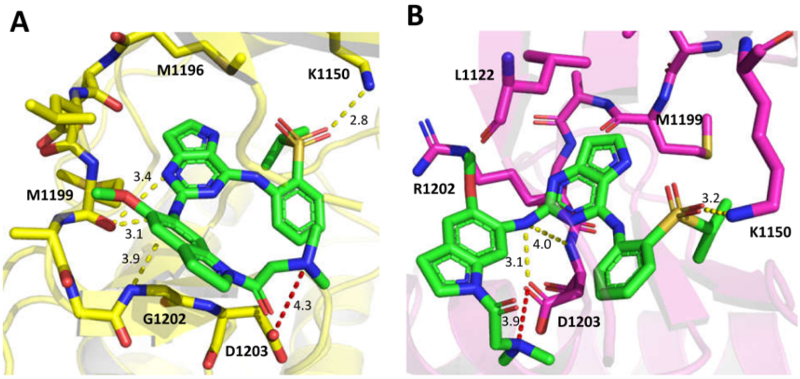

Appendix Figure S6 Molecular docking of XMU-MP-5 with ALK mutant kinases. A, B ALK (L1196M) mutant (PDB ID: 2YFX) was used in (A). Carbon chains of L1196M model were colored in yellow. ALK (G1202R) homology model (B, colored in purple) was developed based on the published X-ray structure of ALK(L1196M) (PDB ID: 2YFX).

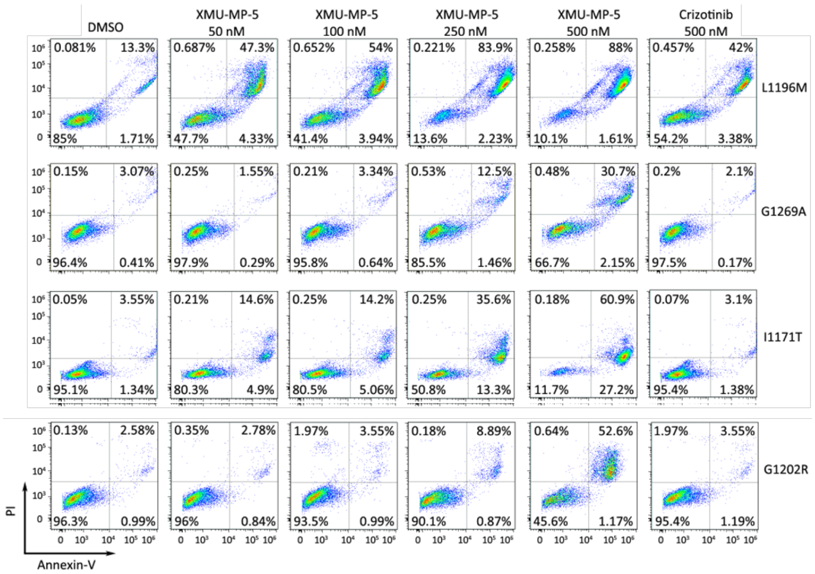

Appendix Figure S7 Cellular apoptosis analysis of XMU-MP-5 against ALK mutant transformed Ba/F3 cell lines.

Induction of cell apoptosis by XMU-MP-5 in ALK mutation Ba/F3 cell lines was assayed by Annexin-V-FLUOS/PI staining using flow cytometry. L1196M cells were treated for 24 hours, G1269A, I1171T and G1202R cell lines were treated for 48 hours.

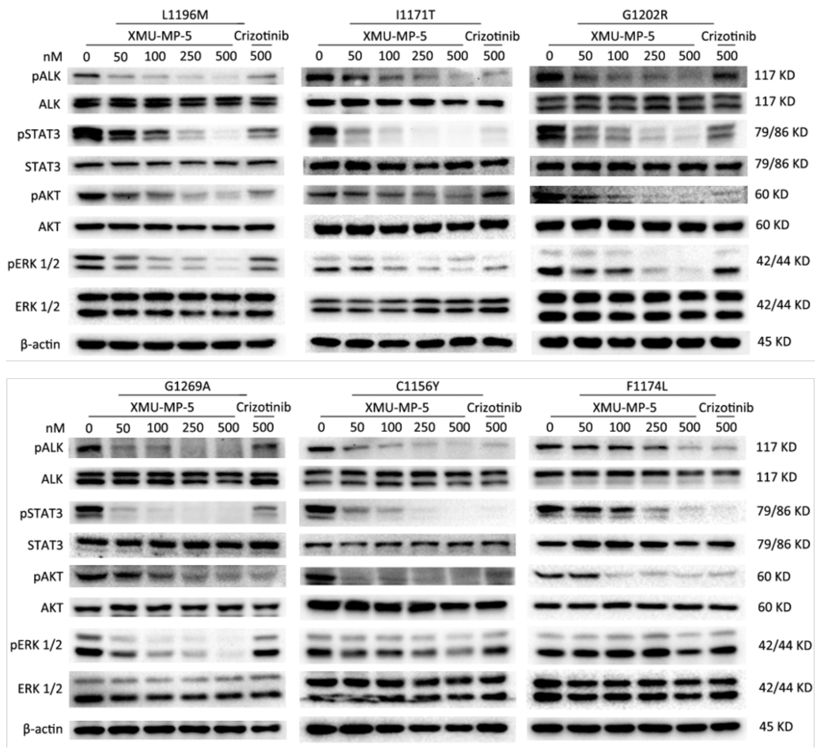

Appendix Figure S8 Effect of XMU-MP-5 on ALK signaling pathways in Ba/F3 cell lines harboring ALK mutations. Cells were all treated with XMU-MP-5 or crizotinib for 4 h then analyzed by immunobloting.

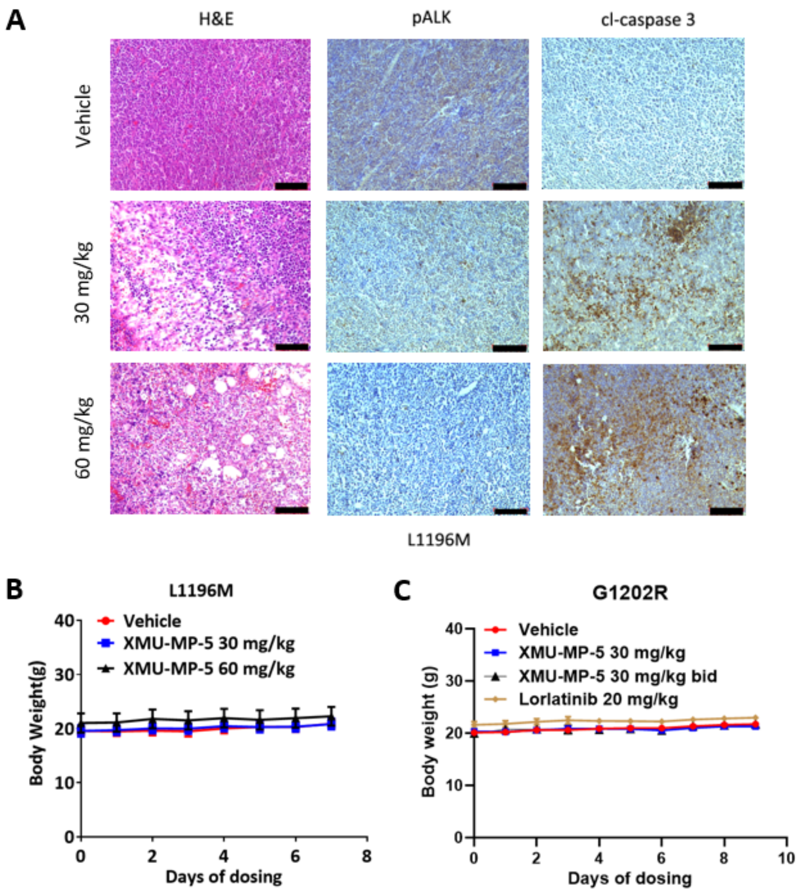

Appendix Figure S9 In vivo efficacy of XMU-MP-5 in xenograft mouse models with ALK mutations.

A H&E and IHC analysis of xenograft tumors from L1196M mice. Scale bar: 100 µm.
B Body weight change of mice bearing L1196M xenograft tumors during XMU-MP-5 treatment. Data were shown as mean ± SD (n = 6).
C Body weights of each group bearing G1202R xenograft tumors were measured every day. Data were shown as mean ± SD (n = 6).

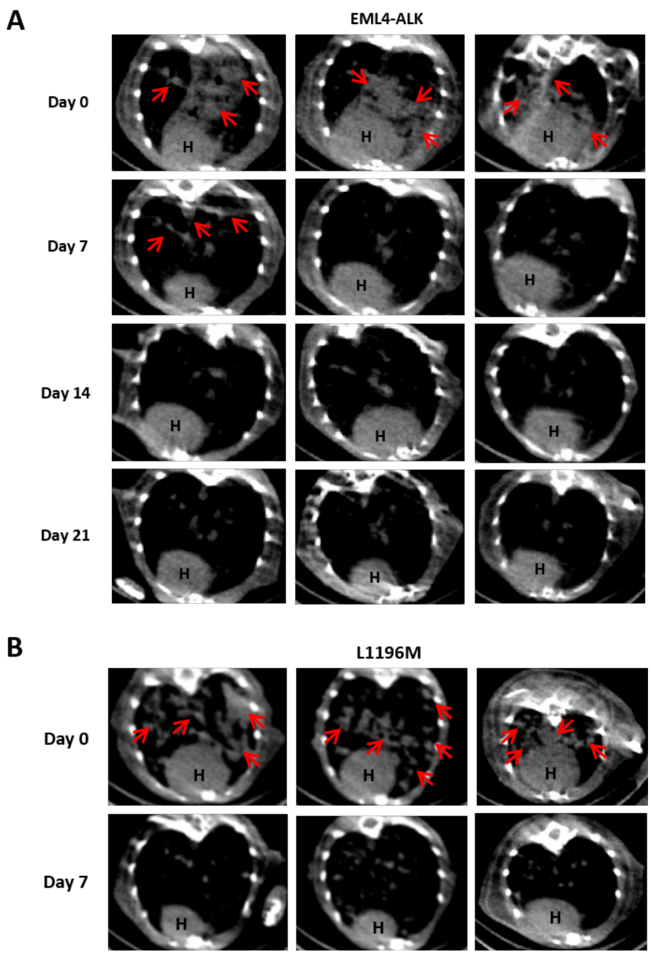

Appendix Figure S10 In vivo efficacy of XMU-MP-5 in different GEM models.
A EML4-ALK transgenic mice were treated with 30 mg/kg XMU-MP-5 twice daily for 3 weeks. Tumor burdens were recorded by CTscan. H: heart; arrow indicated tumor. (n = 3).
B L1196M transgenic mice were treated with 40 mg/kg XMUMP-5 twice daily for 1 week. H: heart; arrow indicated tumor (n = 3).

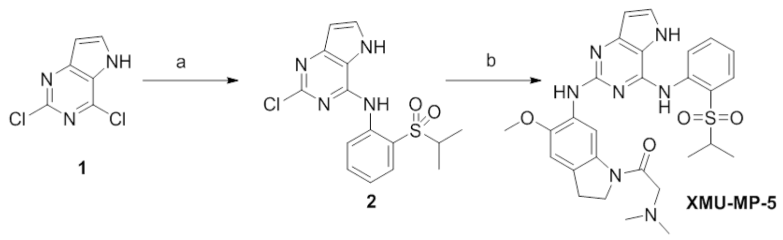

Appendix Figure S11 Synthesis of XMU-MP-5.
Reagents and conditions: a) 2-(isopropylsulfonyl)aniline, TFA, 2-BuOH, 110 ℃; b) 1-(6-amino-5-methoxyindolin-1-yl)-2-(dimethylamino)ethan-1-one, TFA, 2-BuOH, 130 ℃.

Appendix Table S1 The full list of KINOMEscan profiling data of XMU-MP-5.

XMU-MP-5 was profiled against a panel of 468 kinases using KINOMEscan technology, an active site–dependent competition binding assay at 1000 nM. The results were reported as percent of control DMSO (ctrl%), in which lower numbers represent higher affinity binding; ctrl% = (test compound signal − positive control signal)/(negative control signal − positive control signal) × 100; where the negative control was set at 100%, and the positive control compound was set at 0%. The kinase hits with ctrl% less than 1 were highlighted, the lower numbers were marked with the more intense color.

Cmpd ID		XMU-MP-5
Concentration (μM)		1
**DiscoveRx Gene Symbol**	**Entrez Gene Symbol**	
AAK1	AAK1	99
ABL1(E255K)-phosphorylated	ABL1	71
ABL1(F317I)-nonphosphorylated	ABL1	77
ABL1(F317I)-phosphorylated	ABL1	69
ABL1(F317L)-nonphosphorylated	ABL1	61
ABL1(F317L)-phosphorylated	ABL1	88
ABL1(H396P)-nonphosphorylated	ABL1	66
ABL1(H396P)-phosphorylated	ABL1	77
ABL1(M351T)-phosphorylated	ABL1	89
ABL1(Q252H)-nonphosphorylated	ABL1	56
ABL1(Q252H)-phosphorylated	ABL1	91
ABL1(T315I)-nonphosphorylated	ABL1	63
ABL1(T315I)-phosphorylated	ABL1	84
ABL1(Y253F)-phosphorylated	ABL1	81
ABL1-nonphosphorylated	ABL1	53
ABL1-phosphorylated	ABL1	70
ABL2	ABL2	86
ACVR1	ACVR1	98
ACVR1B	ACVR1B	99
ACVR2A	ACVR2A	100
ACVR2B	ACVR2B	98
ACVRL1	ACVRL1	100
ADCK3	CABC1	100

ADCK4	ADCK4	95
AKT1	AKT1	100
AKT2	AKT2	100
AKT3	AKT3	100
ALK	ALK	0.45
ALK(C1156Y)	ALK	0
ALK(L1196M)	ALK	0
AMPK-alpha1	PRKAA1	81
AMPK-alpha2	PRKAA2	88
ANKK1	ANKK1	58
ARK5	NUAK1	100
ASK1	MAP3K5	100
ASK2	MAP3K6	100
AURKA	AURKA	98
AURKB	AURKB	73
AURKC	AURKC	95
AXL	AXL	88
BIKE	BMP2K	98
BLK	BLK	94
BMPR1A	BMPR1A	96
BMPR1B	BMPR1B	77
BMPR2	BMPR2	72
BMX	BMX	100
BRAF	BRAF	100
BRAF(V600E)	BRAF	68
BRK	PTK6	95
BRSK1	BRSK1	100
BRSK2	BRSK2	97
BTK	BTK	83
BUB1	BUB1	71
CAMK1	CAMK1	54
CAMK1B	PNCK	75
CAMK1D	CAMK1D	69
CAMK1G	CAMK1G	92
CAMK2A	CAMK2A	75
CAMK2B	CAMK2B	75
CAMK2D	CAMK2D	87
CAMK2G	CAMK2G	90
CAMK4	CAMK4	100
CAMKK1	CAMKK1	93
CAMKK2	CAMKK2	83
CASK	CASK	100
CDC2L1	CDK11B	94
CDC2L2	CDC2L2	100
CDC2L5	CDK13	0.9
CDK11	CDK19	100
CDK2	CDK2	90
CDK3	CDK3	99

CDK4	CDK4	100
CDK4-cyclinD1	CDK4	77
CDK4-cyclinD3	CDK4	88
CDK5	CDK5	94
CDK7	CDK7	75
CDK8	CDK8	91
CDK9	CDK9	82
CDKL1	CDKL1	64
CDKL2	CDKL2	94
CDKL3	CDKL3	100
CDKL5	CDKL5	67
CHEK1	CHEK1	90
CHEK2	CHEK2	100
CIT	CIT	89
CLK1	CLK1	80
CLK2	CLK2	69
CLK3	CLK3	89
CLK4	CLK4	81
CSF1R	CSF1R	98
CSF1R-autoinhibited	CSF1R	98
CSK	CSK	79
CSNK1A1	CSNK1A1	75
CSNK1A1L	CSNK1A1L	91
CSNK1D	CSNK1D	89
CSNK1E	CSNK1E	94
CSNK1G1	CSNK1G1	98
CSNK1G2	CSNK1G2	85
CSNK1G3	CSNK1G3	76
CSNK2A1	CSNK2A1	72
CSNK2A2	CSNK2A2	91
CTK	MATK	94
DAPK1	DAPK1	100
DAPK2	DAPK2	72
DAPK3	DAPK3	72
DCAMKL1	DCLK1	51
DCAMKL2	DCLK2	100
DCAMKL3	DCLK3	99
DDR1	DDR1	93
DDR2	DDR2	82
DLK	MAP3K12	100
DMPK	DMPK	100
DMPK2	CDC42BPG	82
DRAK1	STK17A	95
DRAK2	STK17B	89
DYRK1A	DYRK1A	72
DYRK1B	DYRK1B	100
DYRK2	DYRK2	50
EGFR	EGFR	98

EGFR(E746-A750del)	EGFR	53
EGFR(G719C)	EGFR	96
EGFR(G719S)	EGFR	86
EGFR(L747-E749del, A750P)	EGFR	59
EGFR(L747-S752del, P753S)	EGFR	68
EGFR(L747-T751del,Sins)	EGFR	84
EGFR(L858R)	EGFR	78
EGFR(L858R,T790M)	EGFR	13
EGFR(L861Q)	EGFR	63
EGFR(S752-I759del)	EGFR	33
EGFR(T790M)	EGFR	9.1
EIF2AK1	EIF2AK1	77
EPHA1	EPHA1	96
EPHA2	EPHA2	91
EPHA3	EPHA3	95
EPHA4	EPHA4	100
EPHA5	EPHA5	89
EPHA6	EPHA6	100
EPHA7	EPHA7	93
EPHA8	EPHA8	91
EPHB1	EPHB1	82
EPHB2	EPHB2	88
EPHB3	EPHB3	99
EPHB4	EPHB4	87
EPHB6	EPHB6	95
ERBB2	ERBB2	73
ERBB3	ERBB3	94
ERBB4	ERBB4	20
ERK1	MAPK3	84
ERK2	MAPK1	78
ERK3	MAPK6	85
ERK4	MAPK4	75
ERK5	MAPK7	82
ERK8	MAPK15	76
ERN1	ERN1	96
FAK	PTK2	15
FER	FER	4.9
FES	FES	12
FGFR1	FGFR1	94
FGFR2	FGFR2	75
FGFR3	FGFR3	96
FGFR3(G697C)	FGFR3	94
FGFR4	FGFR4	94
FGR	FGR	97
FLT1	FLT1	97
FLT3	FLT3	100
FLT3(D835H)	FLT3	88
FLT3(D835V)	FLT3	88

FLT3(D835Y)	FLT3	89
FLT3(ITD)	FLT3	79
FLT3(ITD,D835V)	FLT3	75
FLT3(ITD,F691L)	FLT3	92
FLT3(K663Q)	FLT3	96
FLT3(N841I)	FLT3	97
FLT3(R834Q)	FLT3	100
FLT3-autoinhibited	FLT3	88
FLT4	FLT4	100
FRK	FRK	77
FYN	FYN	94
GAK	GAK	83
GCN2(Kin.Dom.2,S808G)	EIF2AK4	100
GRK1	GRK1	78
GRK2	ADRBK1	100
GRK3	ADRBK2	100
GRK4	GRK4	97
GRK7	GRK7	77
GSK3A	GSK3A	100
GSK3B	GSK3B	90
HASPIN	GSG2	69
HCK	HCK	69
HIPK1	HIPK1	67
HIPK2	HIPK2	78
HIPK3	HIPK3	85
HIPK4	HIPK4	85
HPK1	MAP4K1	100
HUNK	HUNK	97
ICK	ICK	89
IGF1R	IGF1R	1.1
IKK-alpha	CHUK	63
IKK-beta	IKBKB	74
IKK-epsilon	IKBKE	100
INSR	INSR	0.1
INSRR	INSRR	0.8
IRAK1	IRAK1	63
IRAK3	IRAK3	92
IRAK4	IRAK4	68
ITK	ITK	88
JAK1(JH1domain-catalytic)	JAK1	100
JAK1(JH2domain-pseudokinase)	JAK1	97
JAK2(JH1domain-catalytic)	JAK2	71
JAK3(JH1domain-catalytic)	JAK3	77
JNK1	MAPK8	66
JNK2	MAPK9	88
JNK3	MAPK10	81
KIT	KIT	100
KIT(A829P)	KIT	74

KIT(D816H)	KIT	70
KIT(D816V)	KIT	84
KIT(L576P)	KIT	90
KIT(V559D)	KIT	96
KIT(V559D,T670I)	KIT	100
KIT(V559D,V654A)	KIT	100
KIT-autoinhibited	KIT	74
LATS1	LATS1	100
LATS2	LATS2	100
LCK	LCK	90
LIMK1	LIMK1	100
LIMK2	LIMK2	88
LKB1	STK11	100
LOK	STK10	80
LRRK2	LRRK2	92
LRRK2(G2019S)	LRRK2	78
LTK	LTK	5.1
LYN	LYN	91
LZK	MAP3K13	86
MAK	MAK	96
MAP3K1	MAP3K1	87
MAP3K15	MAP3K15	76
MAP3K2	MAP3K2	97
MAP3K3	MAP3K3	68
MAP3K4	MAP3K4	89
MAP4K2	MAP4K2	86
MAP4K3	MAP4K3	100
MAP4K4	MAP4K4	100
MAP4K5	MAP4K5	100
MAPKAPK2	MAPKAPK2	100
MAPKAPK5	MAPKAPK5	78
MARK1	MARK1	100
MARK2	MARK2	93
MARK3	MARK3	91
MARK4	MARK4	100
MAST1	MAST1	83
MEK1	MAP2K1	68
MEK2	MAP2K2	60
MEK3	MAP2K3	69
MEK4	MAP2K4	85
MEK5	MAP2K5	74
MEK6	MAP2K6	92
MELK	MELK	100
MERTK	MERTK	60
MET	MET	84
MET(M1250T)	MET	82
MET(Y1235D)	MET	77
MINK	MINK1	64

MKK7	MAP2K7	42
MKNK1	MKNK1	81
MKNK2	MKNK2	82
MLCK	MYLK3	100
MLK1	MAP3K9	90
MLK2	MAP3K10	69
MLK3	MAP3K11	100
MRCKA	CDC42BPA	97
MRCKB	CDC42BPB	100
MST1	STK4	93
MST1R	MST1R	99
MST2	STK3	86
MST3	STK24	100
MST4	MST4	98
MTOR	MTOR	81
MUSK	MUSK	100
MYLK	MYLK	75
MYLK2	MYLK2	67
MYLK4	MYLK4	91
MYO3A	MYO3A	93
MYO3B	MYO3B	100
NDR1	STK38	73
NDR2	STK38L	100
NEK1	NEK1	89
NEK10	NEK10	83
NEK11	NEK11	97
NEK2	NEK2	99
NEK3	NEK3	77
NEK4	NEK4	93
NEK5	NEK5	91
NEK6	NEK6	96
NEK7	NEK7	100
NEK9	NEK9	100
NIK	MAP3K14	93
NIM1	MGC42105	72
NLK	NLK	97
OSR1	OXSR1	74
p38-alpha	MAPK14	96
p38-beta	MAPK11	92
p38-delta	MAPK13	75
p38-gamma	MAPK12	88
PAK1	PAK1	77
PAK2	PAK2	79
PAK3	PAK3	87
PAK4	PAK4	76
PAK6	PAK6	79
PAK7	PAK7	90
PCTK1	CDK16	74

PCTK2	CDK17	84
PCTK3	CDK18	100
PDGFRA	PDGFRA	74
PDGFRB	PDGFRB	100
PDPK1	PDPK1	97
PFCDPK1(P.falciparum)	CDPK1	85
PFPK5(P.falciparum)	MAL13P1.279	98
PFTAIRE2	CDK15	98
PFTK1	CDK14	97
PHKG1	PHKG1	98
PHKG2	PHKG2	60
PIK3C2B	PIK3C2B	87
PIK3C2G	PIK3C2G	85
PIK3CA	PIK3CA	100
PIK3CA(C420R)	PIK3CA	72
PIK3CA(E542K)	PIK3CA	69
PIK3CA(E545A)	PIK3CA	68
PIK3CA(E545K)	PIK3CA	62
PIK3CA(H1047L)	PIK3CA	89
PIK3CA(H1047Y)	PIK3CA	86
PIK3CA(I800L)	PIK3CA	89
PIK3CA(M1043I)	PIK3CA	84
PIK3CA(Q546K)	PIK3CA	82
PIK3CB	PIK3CB	72
PIK3CD	PIK3CD	94
PIK3CG	PIK3CG	100
PIK4CB	PI4KB	45
PIKFYVE	PIKFYVE	92
PIM1	PIM1	75
PIM2	PIM2	98
PIM3	PIM3	84
PIP5K1A	PIP5K1A	92
PIP5K1C	PIP5K1C	100
PIP5K2B	PIP4K2B	100
PIP5K2C	PIP4K2C	76
PKAC-alpha	PRKACA	95
PKAC-beta	PRKACB	100
PKMYT1	PKMYT1	100
PKN1	PKN1	76
PKN2	PKN2	99
PKNB(M.tuberculosis)	pknB	100
PLK1	PLK1	82
PLK2	PLK2	68
PLK3	PLK3	65
PLK4	PLK4	93
PRKCD	PRKCD	93
PRKCE	PRKCE	83
PRKCH	PRKCH	87

PRKCI	PRKCI	77
PRKCQ	PRKCQ	100
PRKD1	PRKD1	85
PRKD2	PRKD2	100
PRKD3	PRKD3	84
PRKG1	PRKG1	90
PRKG2	PRKG2	72
PRKR	EIF2AK2	85
PRKX	PRKX	99
PRP4	PRPF4B	100
PYK2	PTK2B	74
QSK	KIAA0999	100
RAF1	RAF1	100
RET	RET	91
RET(M918T)	RET	92
RET(V804L)	RET	100
RET(V804M)	RET	100
RIOK1	RIOK1	85
RIOK2	RIOK2	84
RIOK3	RIOK3	95
RIPK1	RIPK1	91
RIPK2	RIPK2	100
RIPK4	RIPK4	81
RIPK5	DSTYK	65
ROCK1	ROCK1	92
ROCK2	ROCK2	73
ROS1	ROS1	18
RPS6KA4(Kin.Dom.1-N-terminal)	RPS6KA4	100
RPS6KA4(Kin.Dom.2-C-terminal)	RPS6KA4	85
RPS6KA5(Kin.Dom.1-N-terminal)	RPS6KA5	96
RPS6KA5(Kin.Dom.2-C-terminal)	RPS6KA5	100
RSK1(Kin.Dom.1-N-terminal)	RPS6KA1	95
RSK1(Kin.Dom.2-C-terminal)	RPS6KA1	100
RSK2(Kin.Dom.1-N-terminal)	RPS6KA3	86
RSK2(Kin.Dom.2-C-terminal)	RPS6KA3	81
RSK3(Kin.Dom.1-N-terminal)	RPS6KA2	91
RSK3(Kin.Dom.2-C-terminal)	RPS6KA2	100
RSK4(Kin.Dom.1-N-terminal)	RPS6KA6	85
RSK4(Kin.Dom.2-C-terminal)	RPS6KA6	91
S6K1	RPS6KB1	87
SBK1	SBK1	73
SGK	SGK1	72
SgK110	SgK110	96
SGK2	SGK2	72
SGK3	SGK3	63
SIK	SIK1	95
SIK2	SIK2	100
SLK	SLK	95

SNARK	NUAK2	57
SNRK	SNRK	88
SRC	SRC	94
SRMS	SRMS	76
SRPK1	SRPK1	100
SRPK2	SRPK2	100
SRPK3	SRPK3	84
STK16	STK16	82
STK33	STK33	81
STK35	STK35	100
STK36	STK36	90
STK39	STK39	72
SYK	SYK	90
TAK1	MAP3K7	74
TAOK1	TAOK1	97
TAOK2	TAOK2	80
TAOK3	TAOK3	86
TBK1	TBK1	88
TEC	TEC	94
TESK1	TESK1	100
TGFBR1	TGFBR1	98
TGFBR2	TGFBR2	100
TIE1	TIE1	91
TIE2	TEK	92
TLK1	TLK1	100
TLK2	TLK2	89
TNIK	TNIK	90
TNK1	TNK1	100
TNK2	TNK2	55
TNNI3K	TNNI3K	100
TRKA	NTRK1	94
TRKB	NTRK2	63
TRKC	NTRK3	67
TRPM6	TRPM6	85
TSSK1B	TSSK1B	100
TSSK3	TSSK3	92
TTK	TTK	71
TXK	TXK	100
TYK2(JH1domain-catalytic)	TYK2	64
TYK2(JH2domain-pseudokinase)	TYK2	59
TYRO3	TYRO3	88
ULK1	ULK1	79
ULK2	ULK2	71
ULK3	ULK3	72
VEGFR2	KDR	74
VPS34	PIK3C3	57
VRK2	VRK2	84
WEE1	WEE1	98

WEE2	WEE2	93
WNK1	WNK1	64
WNK2	WNK2	65
WNK3	WNK3	72
WNK4	WNK4	92
YANK1	STK32A	84
YANK2	STK32B	87
YANK3	STK32C	100
YES	YES1	89
YSK1	STK25	86
YSK4	MAP3K19	63
ZAK	ZAK	100
ZAP70	ZAP70	49

Appendix Table S2 S score of XMU-MP-5 and approved ALK TKIs.

The S score of XMU-MP-5 was determined according to KINOMEscan profile data, data for crizotinib, ceritinib, alectinib and brigatinib were acquired from published reference. ND = Not Determined.

TKI	S(10)	S(35)
**XMU-MP-5**	0.02	0.03
**Crizotinib**	ND	0.21
**Ceritinib**	0.19	ND
**Alectinib**	0.07	ND
**Brigatinib**	0.34	ND

Appendix Table S3 Pharmacokinetic properties of XMU-MP-5 in mice.

The pharmacokinetics of XMU-MP-5 were determined following single intravenous and oral administration in ICR mice (N = 3/each point) at the dose indicated. Blood samples were collected at 0.08, 0.25, 0.5, 1, 2, 4, 8 & 24 hr (IV) & 0.08, 0.25, 0.5, 1, 2, 4, 6, 8 hr (PO) post dose. Then the samples were quantified by LC-MS/MS, and data analysis was conducted using WinNonlin V6.3. intravenous injection, PO = oral delivery, T_{max} = time of maximum plasma concentration, C_{max} = maximum plasma concentration, = area under the curve (measure of exposure), $T_{1/2}$ = half life, CL = plasma clearance, Vz = volume of distribution, F = oral bioavailal

**Route**	**Dose**	**$t_{1/2}$**	**T_{max}**	**C_{max}**	**C_0**	**$AUC_{(0-t)}$**	**$AUC_{(0-\infty)}$**	**Vz**	**Cl**	**$MRT_{(0-\infty)}$**	**F**
	**(mg/kg)**	**hr**	**hr**	**ng/mL**	**ng/mL**	**ng/mL*hr**	**ng/mL*hr**	**L/kg**	**L/hr/kg**	**hr**	**%**
IV	5	3.99	0.08	4303.44	5718.82	3299.95	3311.99	8.70	1.51	1.74	-
IV	30	4.93	-	-	10200	13100	13200	6.70	2.27	2.95	-
IV	60	4.01	-	-	16300	26200	26400	7.71	2.27	3.39	-
PO	10	1.53	0.50	474.50	-	1205.37	1274.20	-	-	3.08	18.26

Appendix Table S4 X-ray data collection and refinement statistics.

	ALK S1281G/XMU-MP-5
**PDB ID**	7BTT
**Data collection**	
Space group	$P2_12_12_1$
Cell dimensions	
a, *b*, *c* (Å)	51.5, 57.4, 105.4
[illegible]°	90.0, 90.0, 90.0
Resolution (Å)	50.0-1.86 (1.90-1.86) *
R_{pim}	0.066 (0.426)
[illegible]	10.8 (1.9)
Completeness (%)	99.4 (99.8)
Redundancy	4.1 (4.3)
**Refinement**	
Resolution (Å)	46.3-1.86
No. reflections	26858
R_{work} / R_{free}	0.187/0.209
No. atoms	
Protein	2391
Ligand/ion	40
Water	157
B-factors	
Protein	36.7
Ligand/ion	34.5
Water	39.6
R.m.s. deviations	
Bond lengths (Å)	0.007
Bond angles (°)	0.938
Ramachandran Plot	
Favored regions (%)	98.33
Allowed regions (%)	1.67
Outliers (%)	0.00

*Values in parentheses are for highest-resolution shell.

Appendix Table S5 IC_{50} values of XMU-MP-5 against wild type and ALK mutant Ba/F3 cell lines[a].

Compound (IC_{50}, nM)	WT	EML4-ALK	L1196M	G1269A	I1171T	G1202R	S1206Y	C1156Y	F1174L
XMU-MP-5	4098	4.15	12	30.9	16.3	49.4	27.4	22.9	123.2
Crizotinib	1116	45.86	467.3	305.1	145.4	556.3	71.4	73.2	81
Alectinib	ND	16.9	167.3	165.1	158	478.9	ND	103.6	47.5
Ceritinib	ND	1.2	6.84	1.8	1.5	206.1	ND	6.1	53.6
Brigatinib	ND	17.3	38.5	14	86.7	432.8	ND	17.2	47.5
Lorlatinib	ND	1.5	56.9	31.5	20.8	89.5	ND	5.9	2.4

[a] Antiproliferative activity was determined by the MTS assay. ND means not determined.

Appendix Table S6 Fold change IC_{50}s of ALK mutant Ba/F3 cell lines to WT Ba/F3 cell lines.

Fold change IC_{50}	EML4-ALK	L1196M	G1269A	I1171T	G1202R	S1206Y	C1156Y	F1174L
XMU-MP-5	987	341.5	132.6	251.4	83	149.6	179	33.3
Crizotinib	24.3	2.4	3.7	7.7	2	15.6	13.2	13.8
