# Supplementary material for: A new ALK inhibitor overcomes resistance to first‐ and second‐generation inhibitors in NSCLC
Source: EMBO Mol Med. 2021 Nov 30;14(1):e14296. doi: 10.15252/emmm.202114296 (PMC8749467; doi:10.15252/emmm.202114296)
Supplement: Supplementary file 2 — Source Data for Appendix [file EMMM-14-e14296-s005.zip › EMM-2021-14296-V3-FigureS5_Source_Data-sd.pdf]

HE: heart of vehicle group

HE: heart of 60 mg/kg group

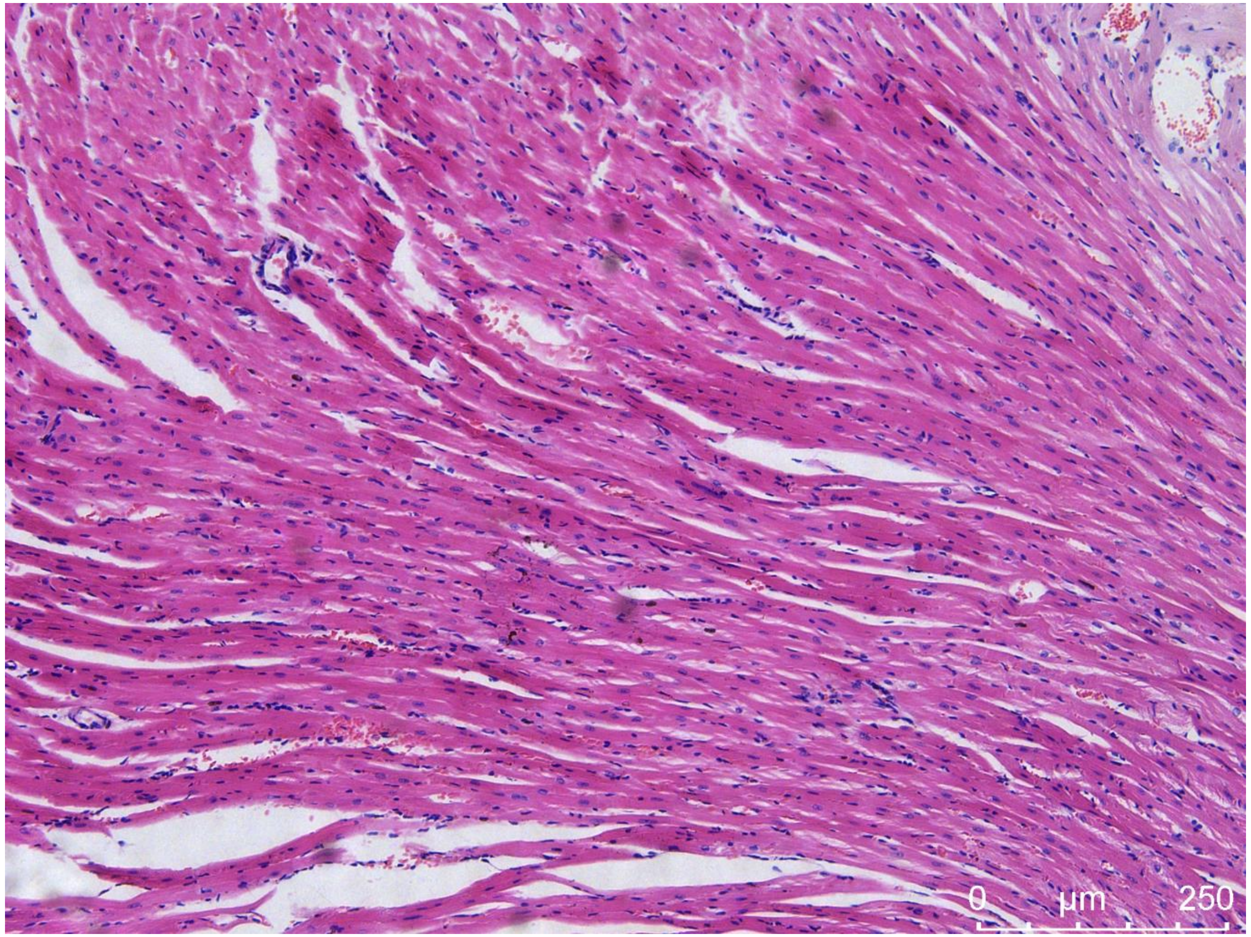

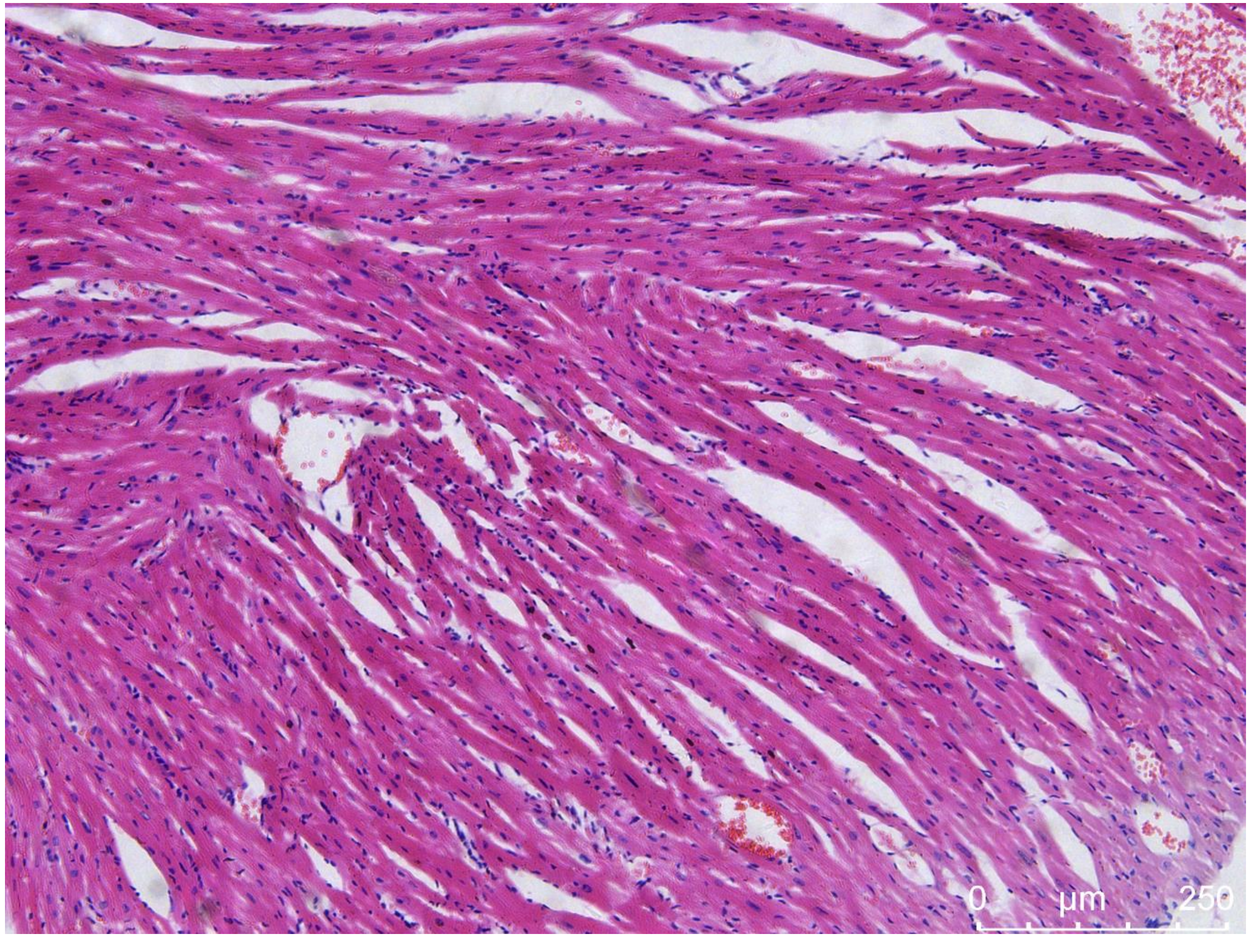

Appendix Figure S5 D

HE: kidney of vehicle group

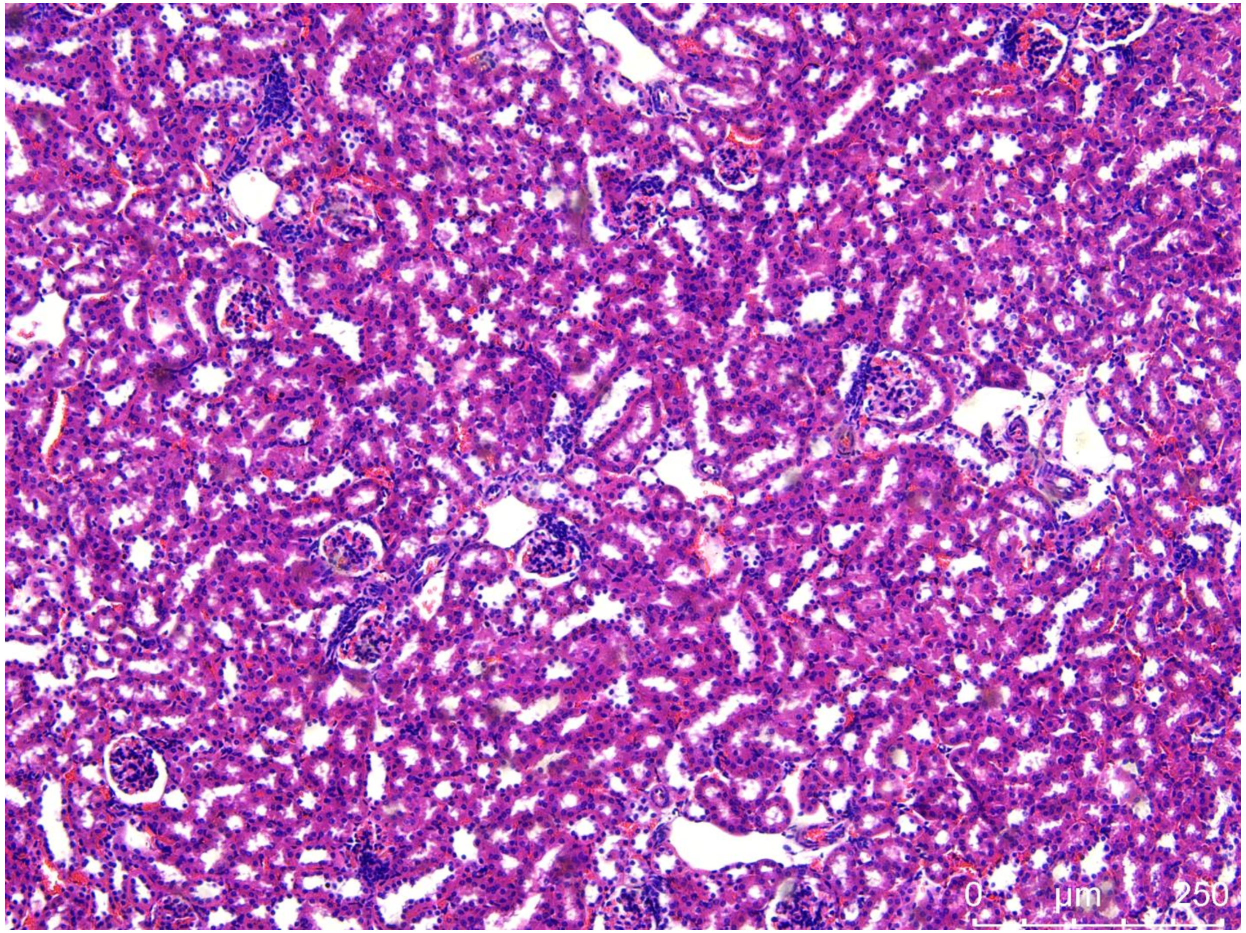

HE: kidney of 60 mg/kg group

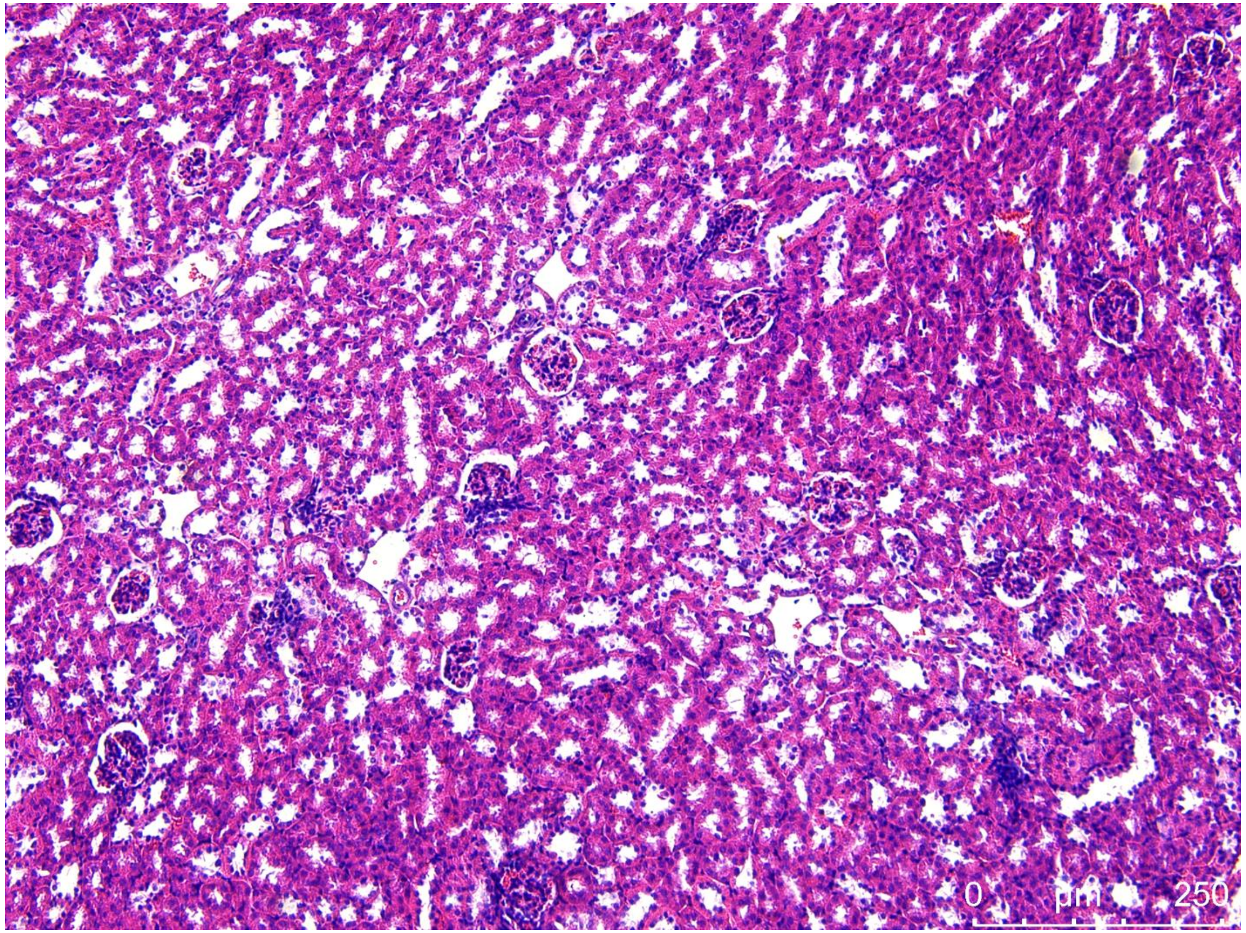

Appendix Figure S5 D

HE: liver of vehicle group

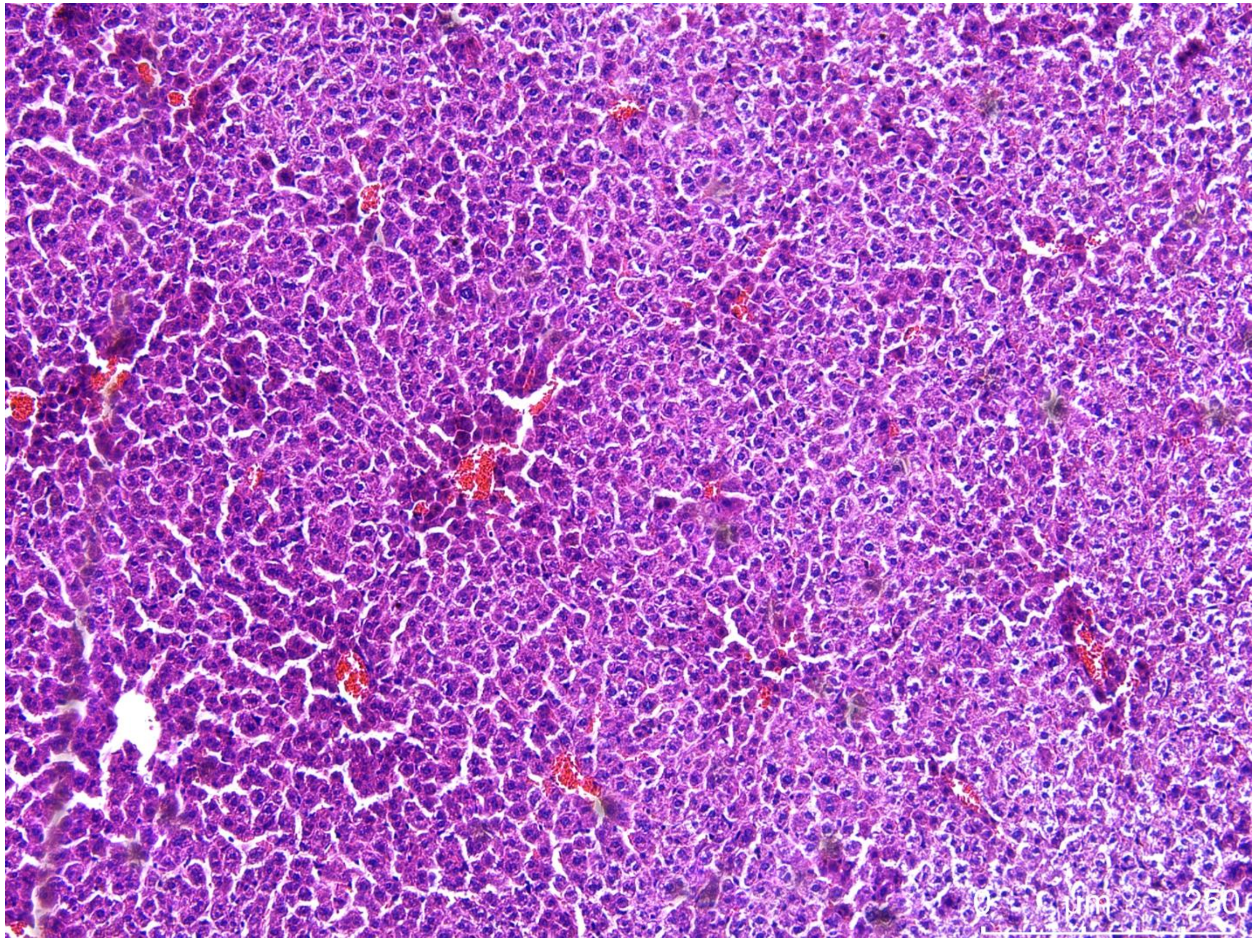

HE: liver of 60 mg/kg group

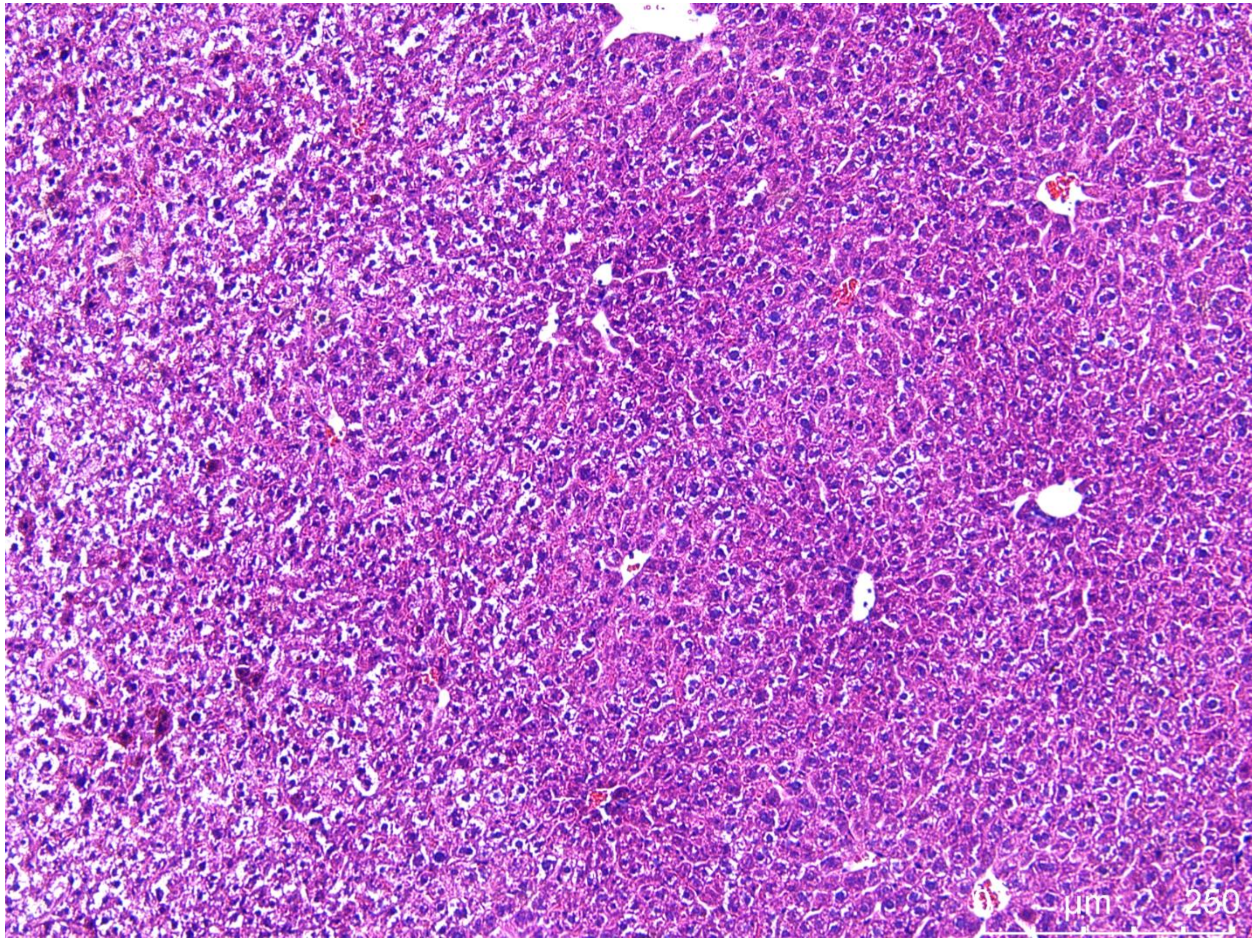

Appendix Figure S5 D

HE: lung of vehicle group

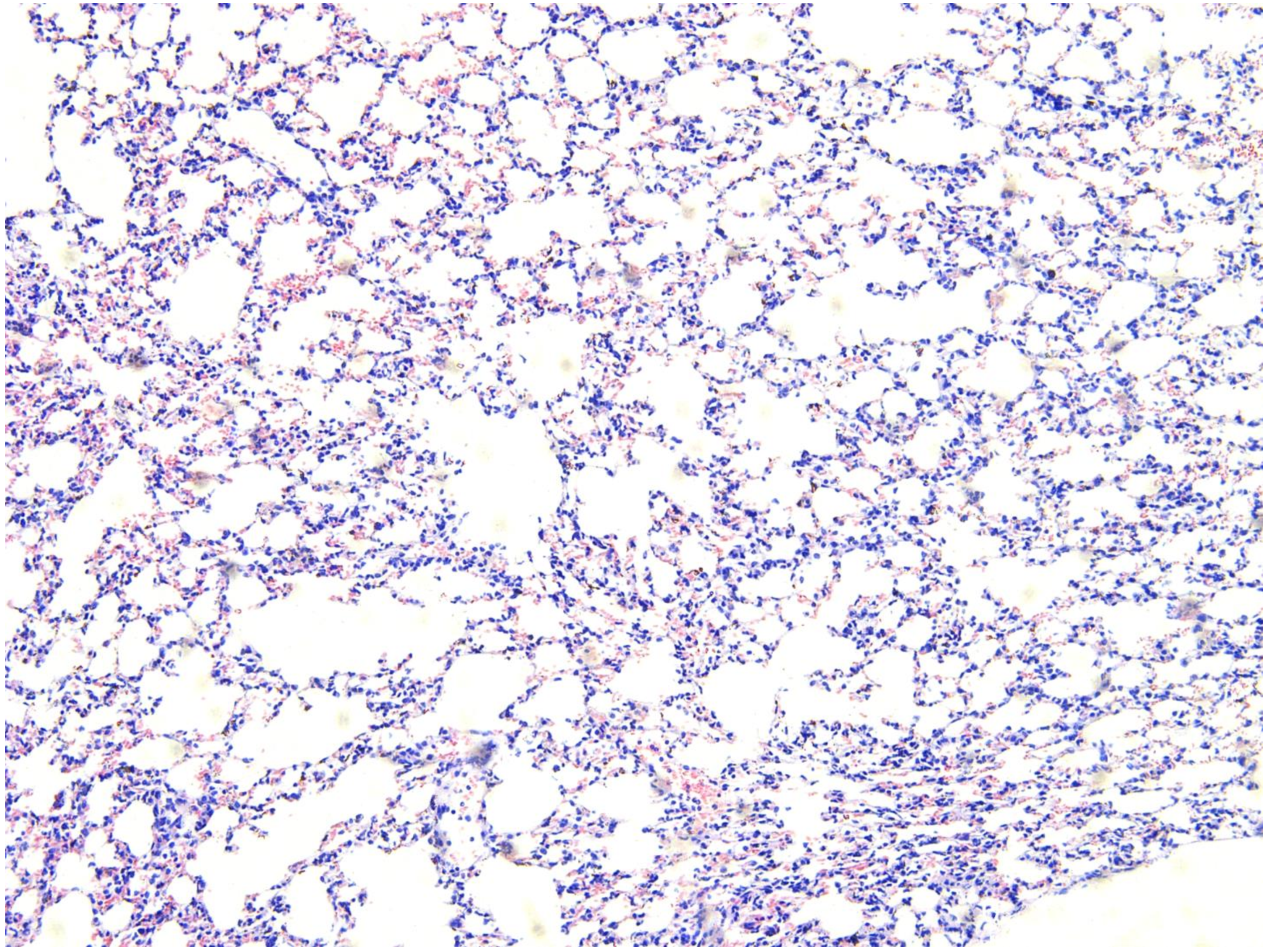

HE: lung of 60 mg/kg group

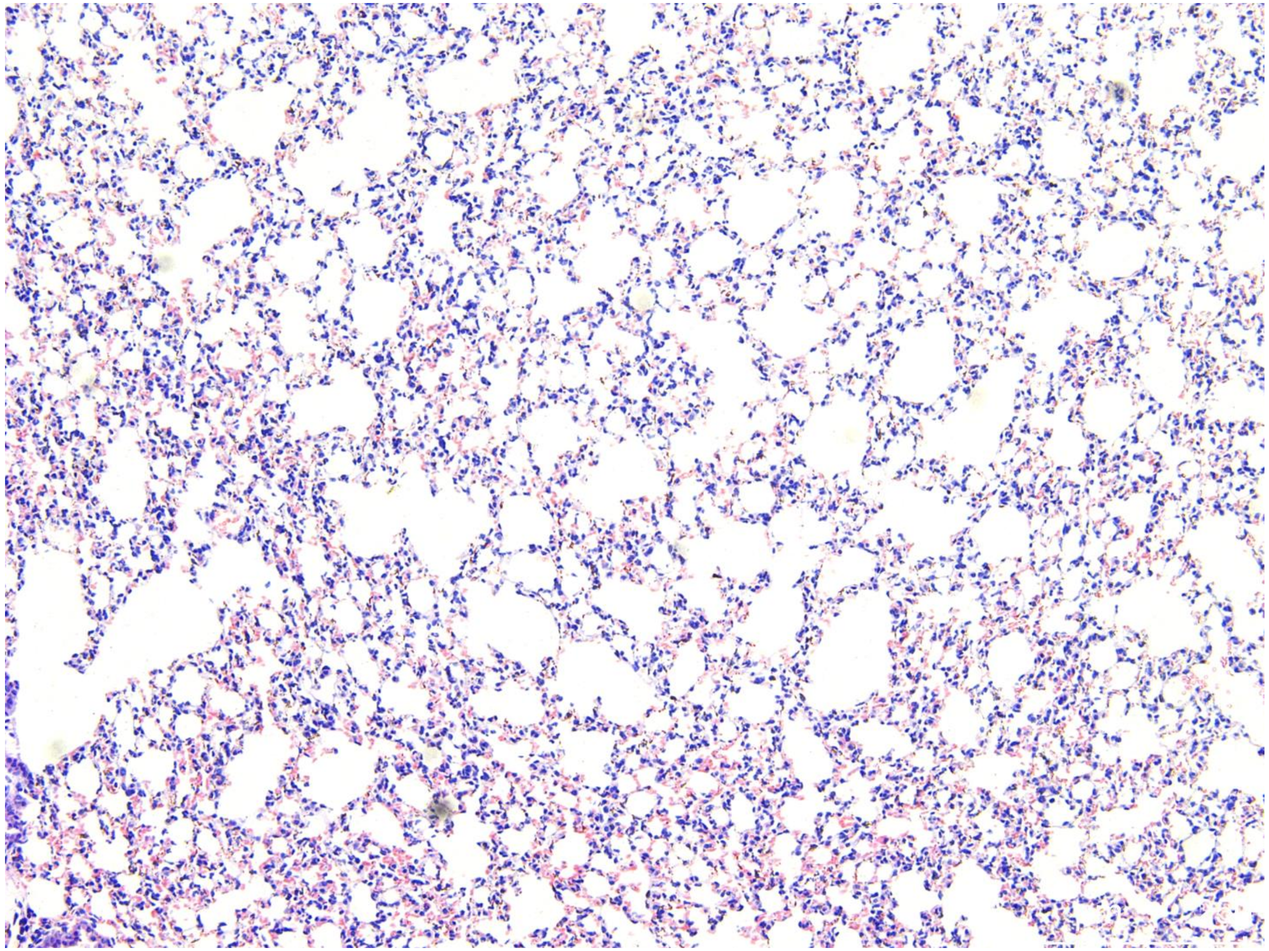

Appendix Figure S5 D

HE: spleen of vehicle group

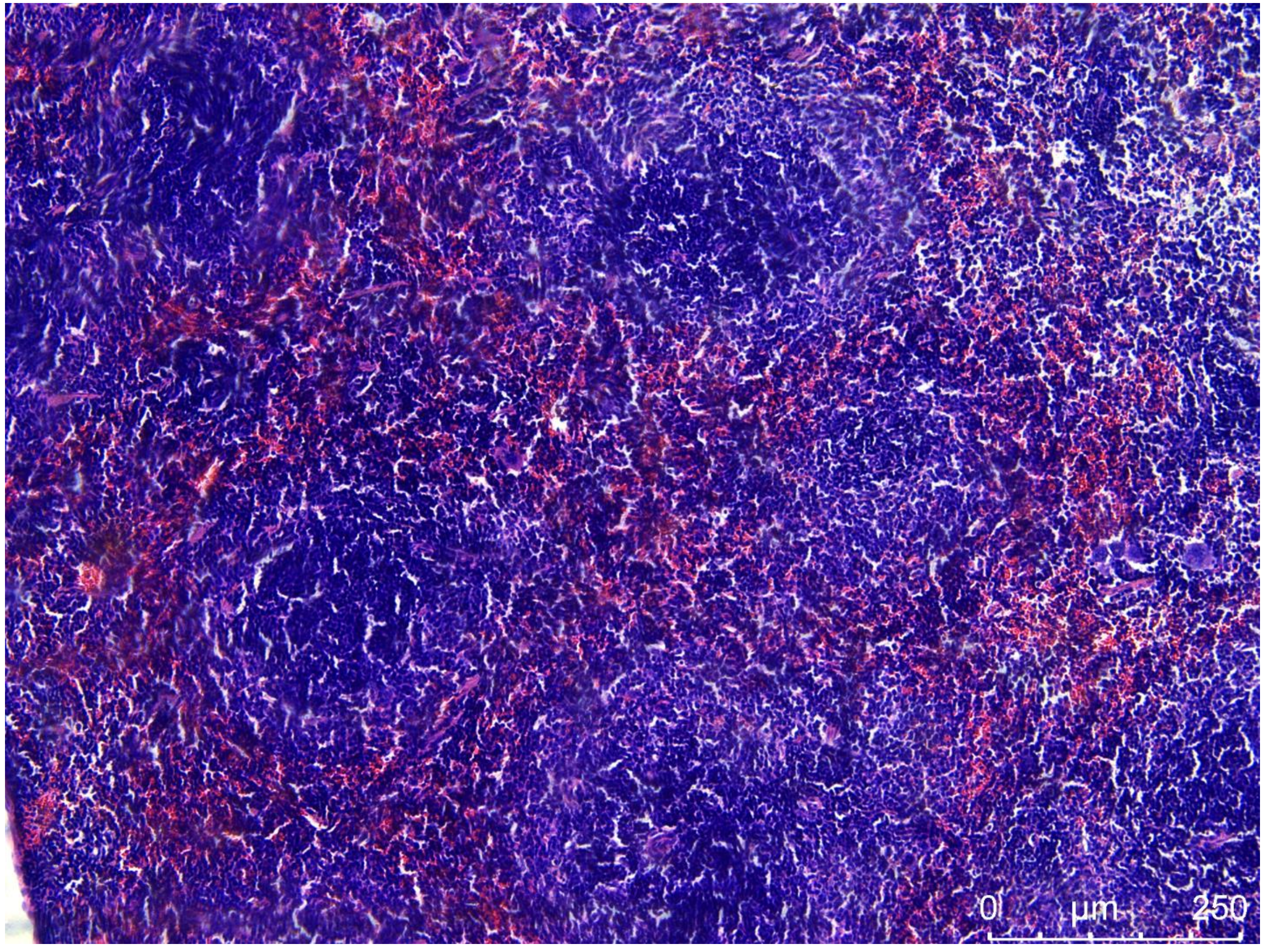

HE: spleen of 60 mg/kg group

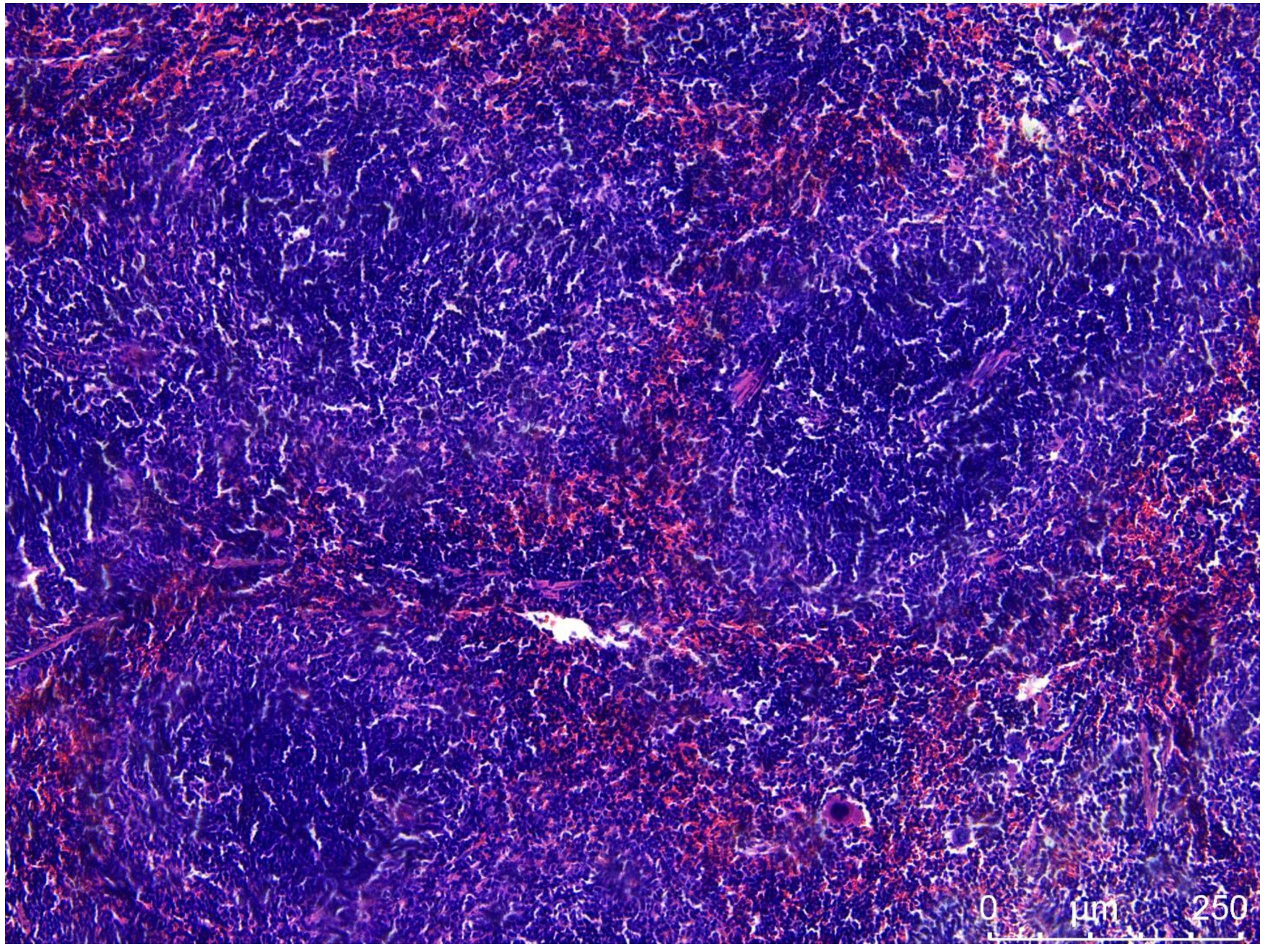

Appendix Figure S5 D
