# Supplementary material for: A new ALK inhibitor overcomes resistance to first‐ and second‐generation inhibitors in NSCLC
Source: EMBO Mol Med. 2021 Nov 30;14(1):e14296. doi: 10.15252/emmm.202114296 (PMC8749467; doi:10.15252/emmm.202114296)
Supplement: Supplementary file 2 — Source Data for Appendix [file EMMM-14-e14296-s005.zip › EMM-2021-14296-V3-FigureS8_Source_Data-sd.pdf]

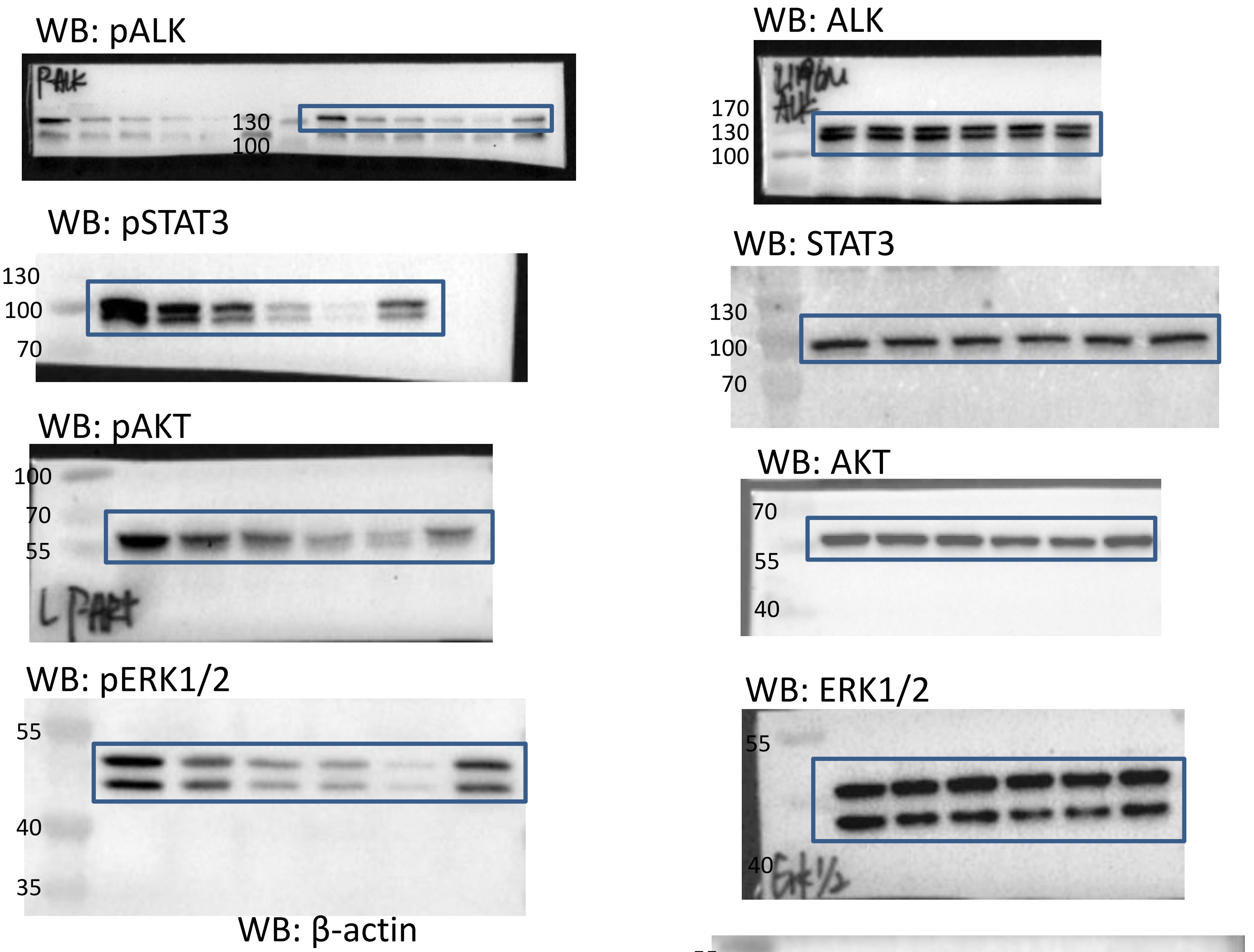

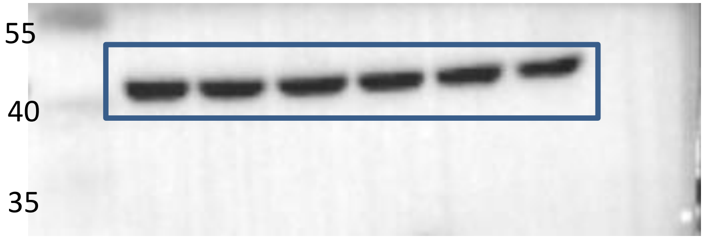

Appendix FigureS8 L1196M

All the different proteins were detected on different blots without stripping.

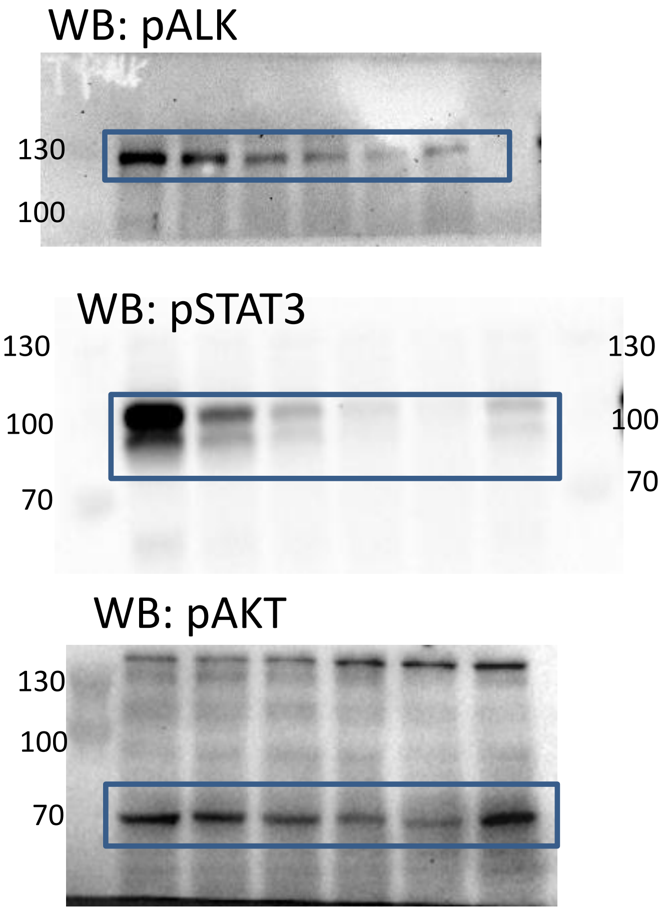

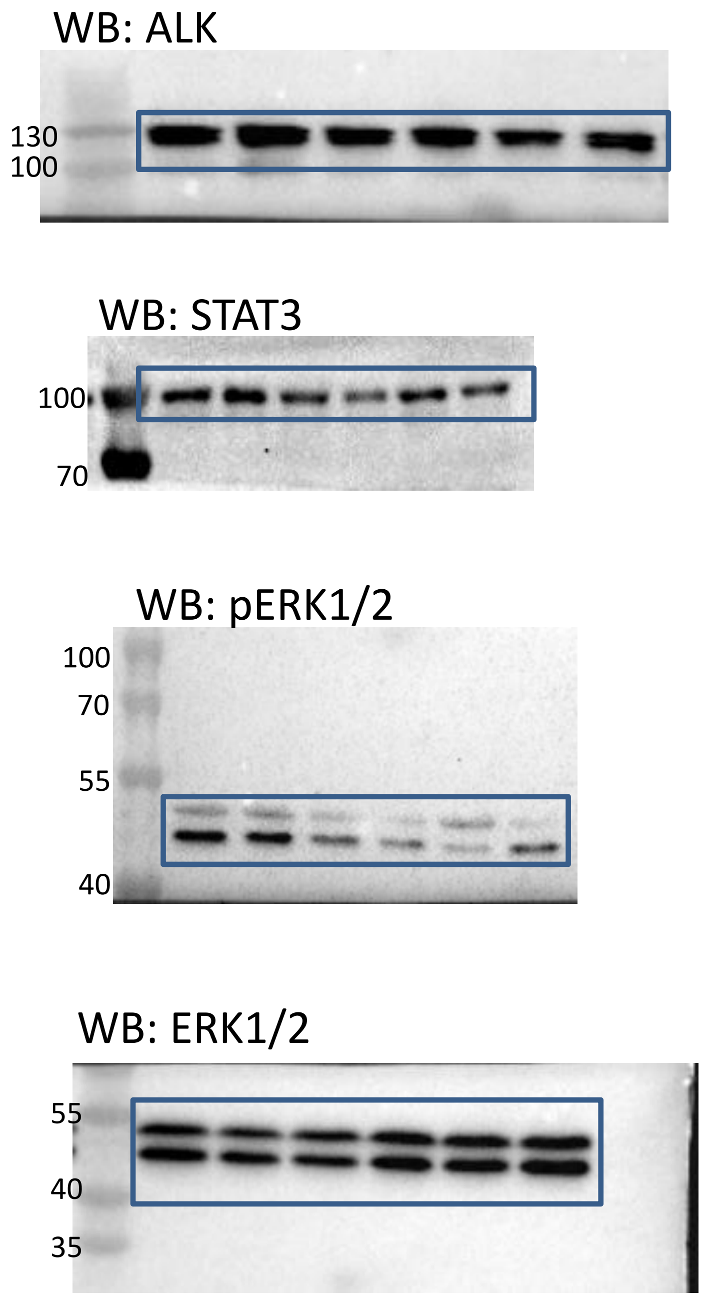

Appendix FigureS8 I1171T

All the different proteins were detected on different blots without stripping.

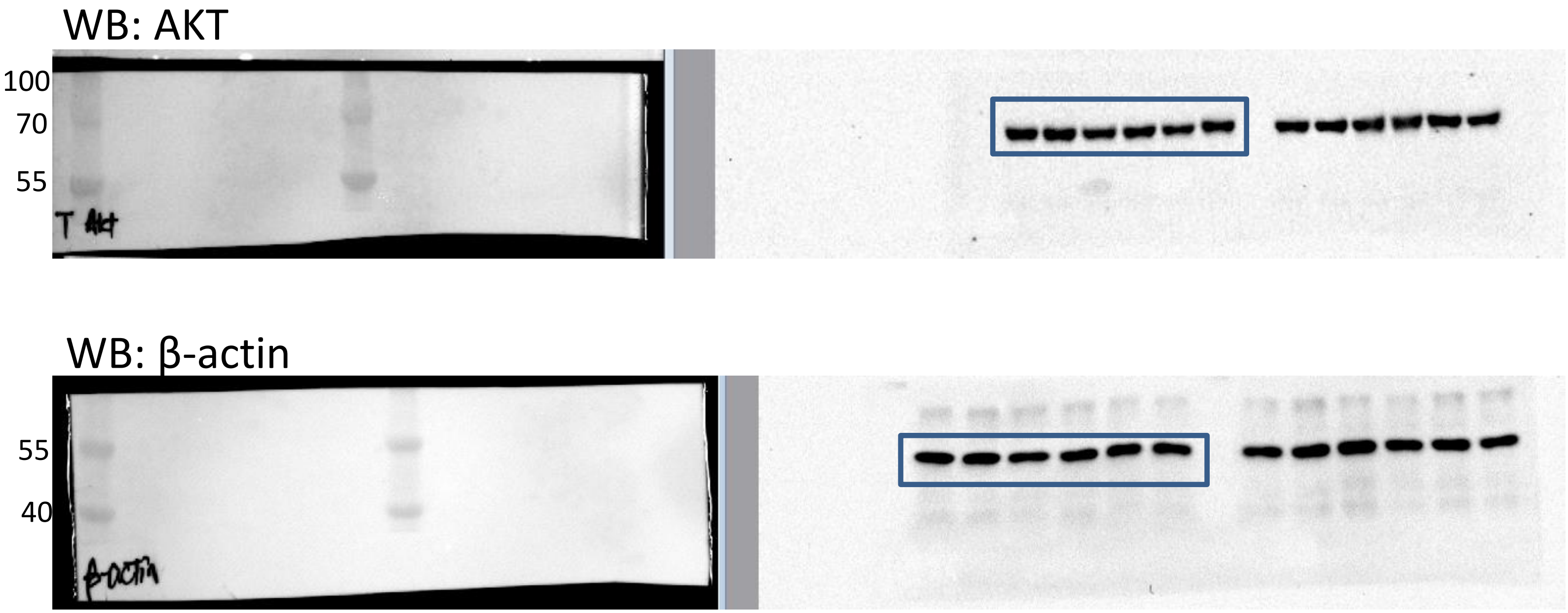

Appendix FigureS8 I1171T

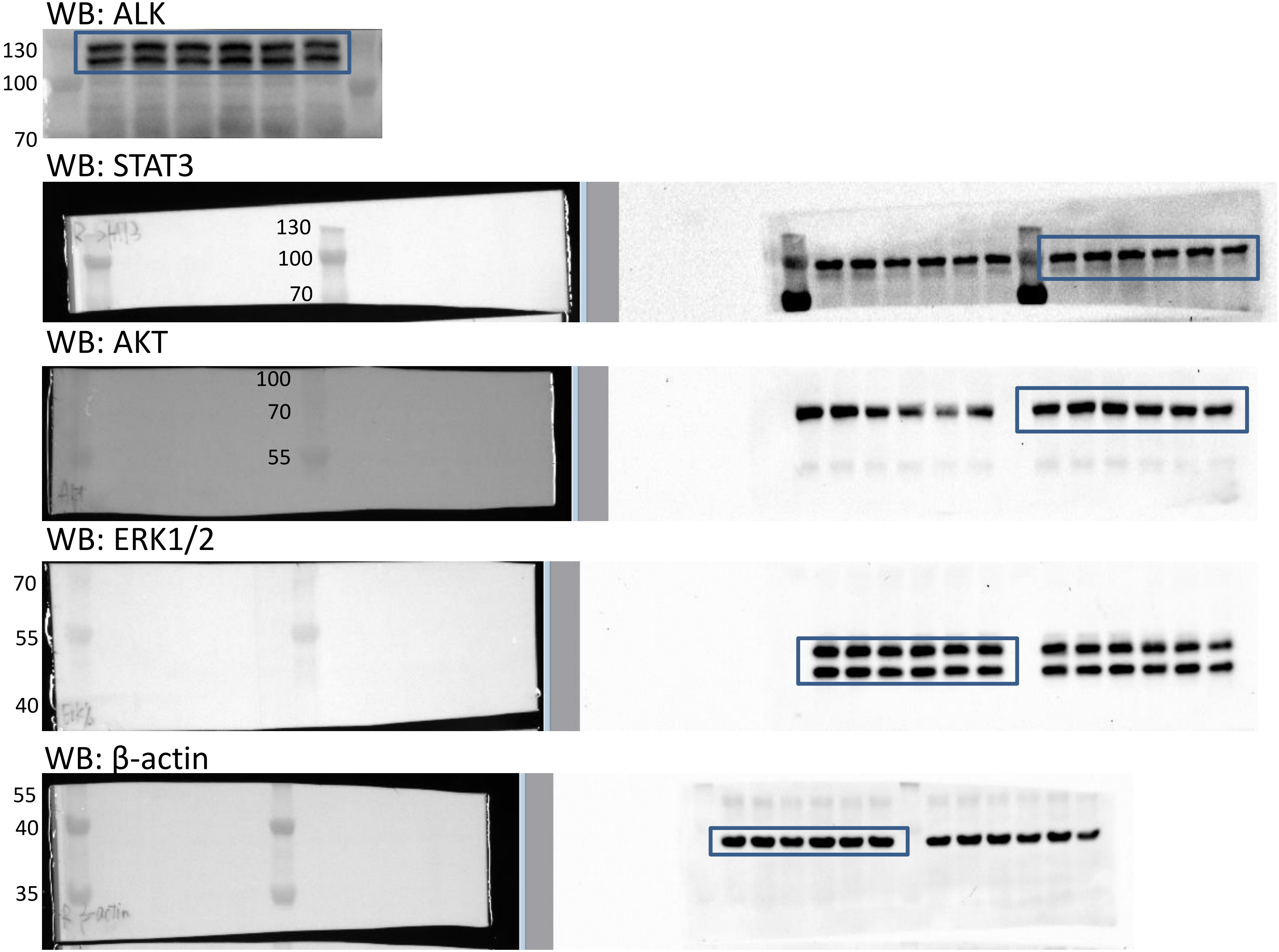

Appendix FigureS8 G1202R

All the different proteins were detected on different blots without stripping.

WB: pALK

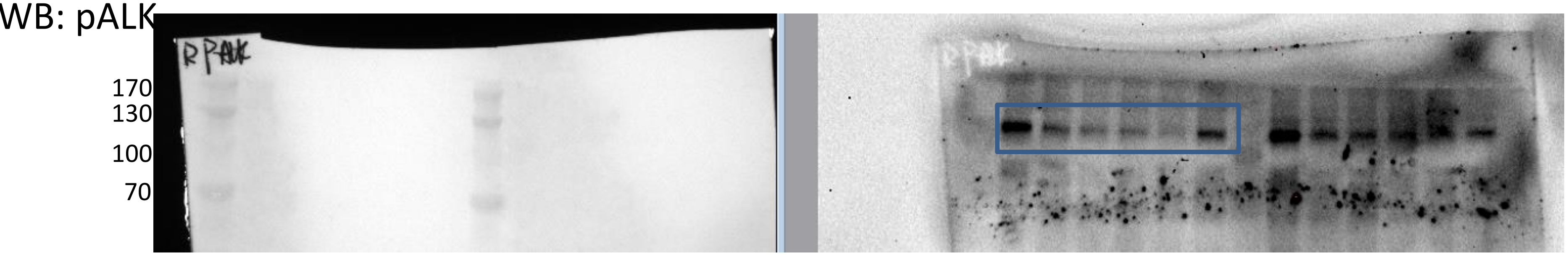

WB: pSTAT3

WB: pERK1/2

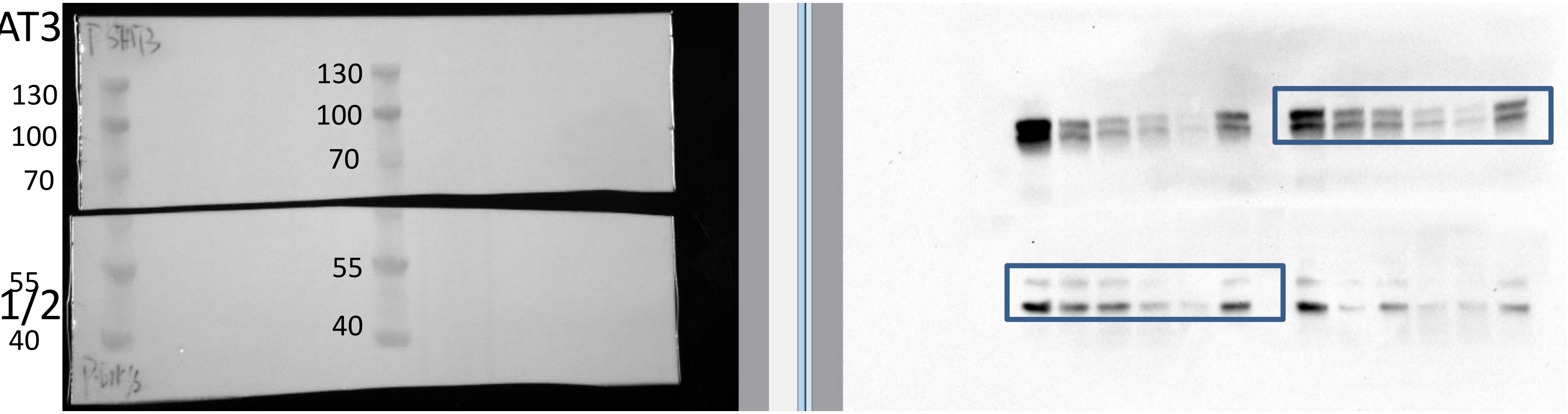

WB: pAKT

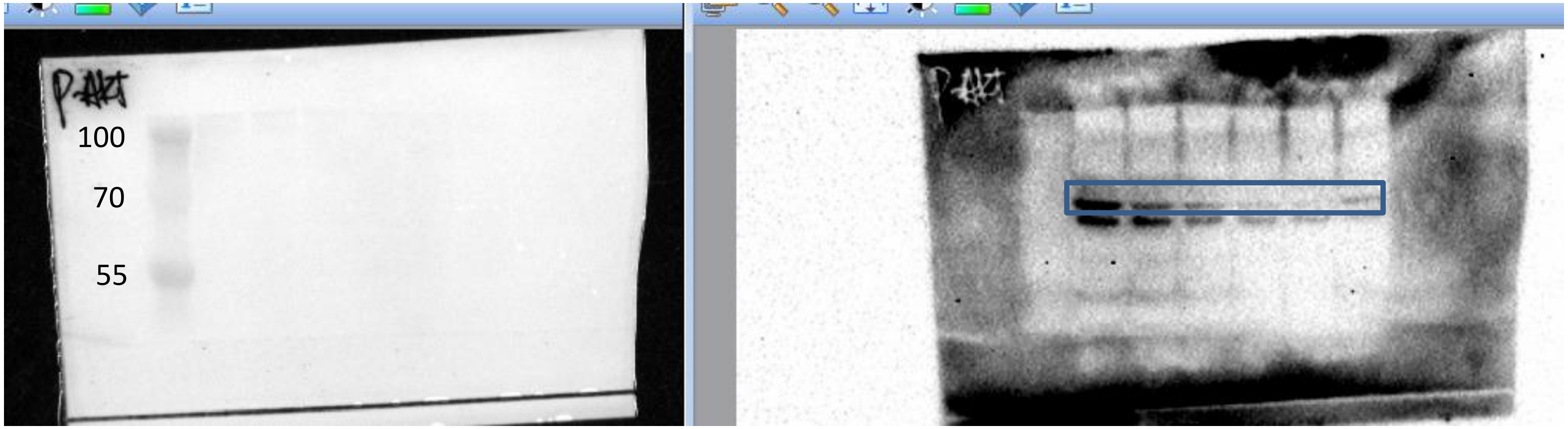

Appendix FigureS8 G1202R

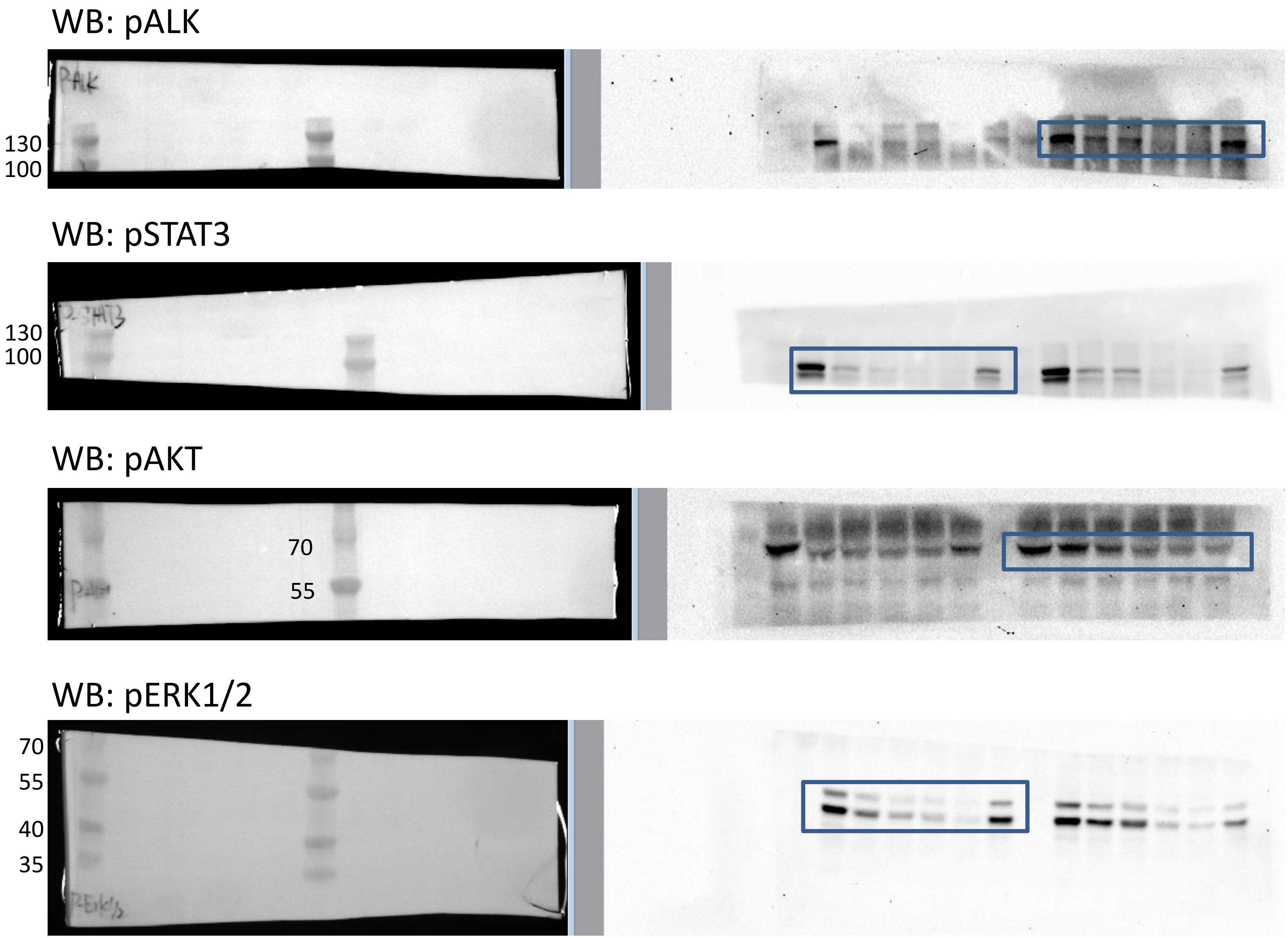

Appendix FigureS8 G1269A

All the different proteins were detected on different blots without stripping.

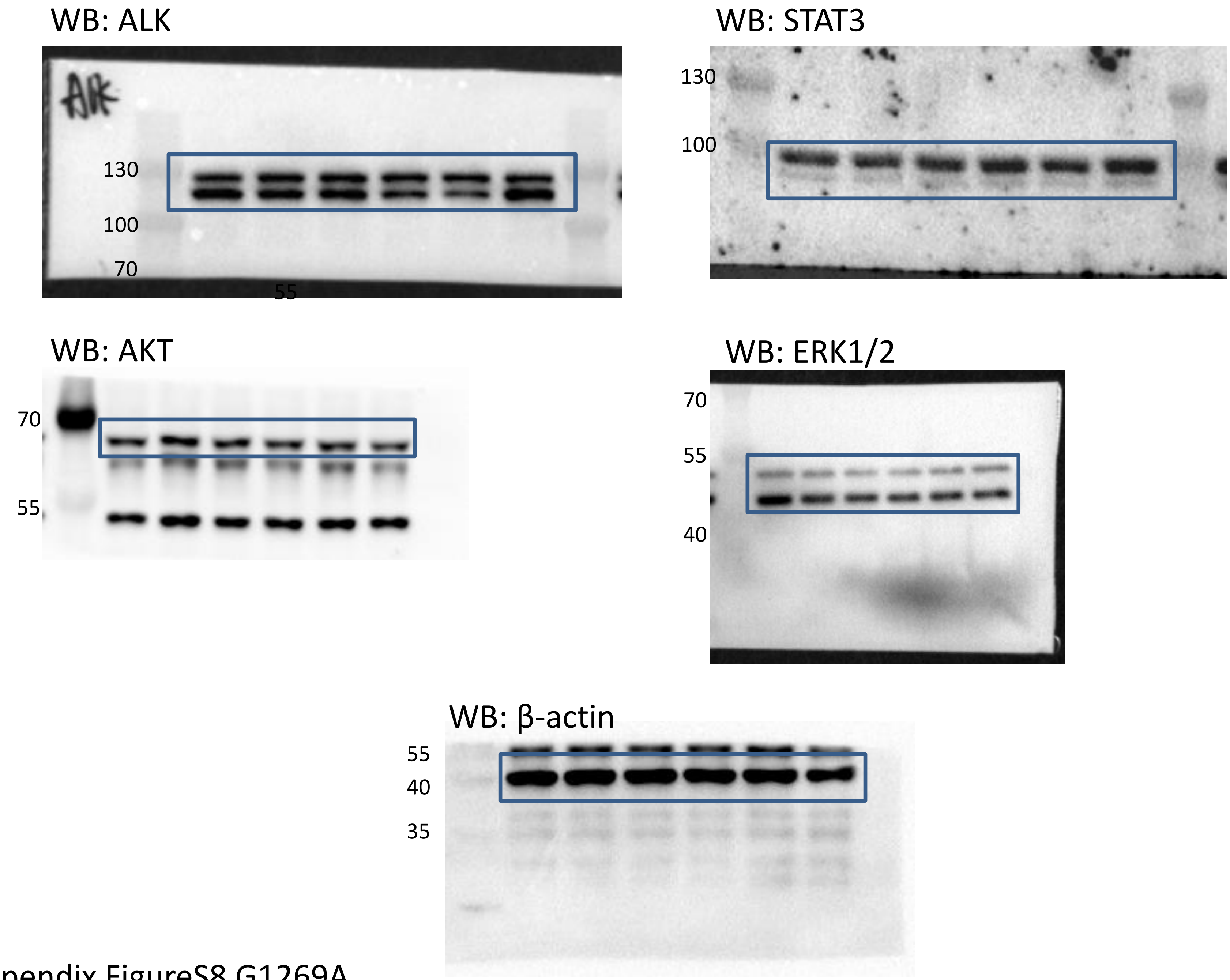

Appendix FigureS8 G1269A

WB: pALK

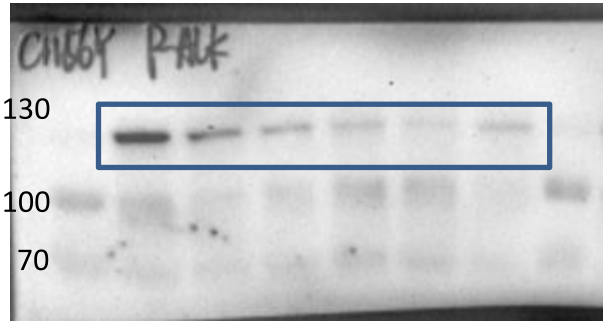

WB: ALK

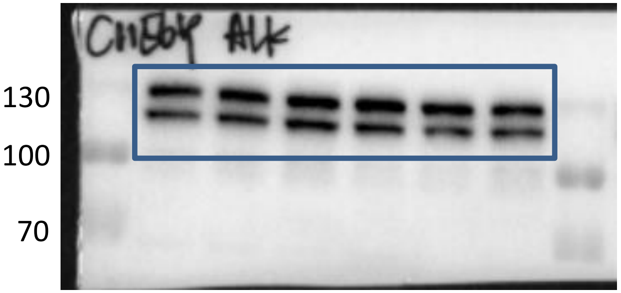

WB: pSTAT3

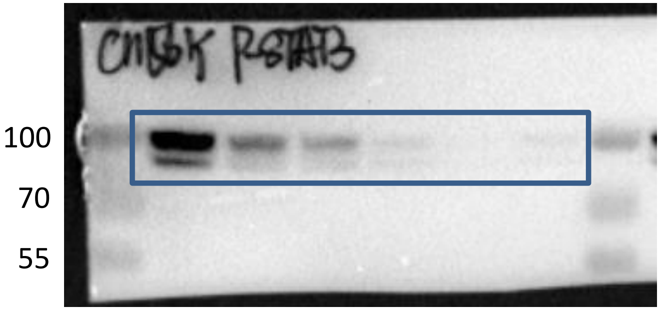

WB: STAT3

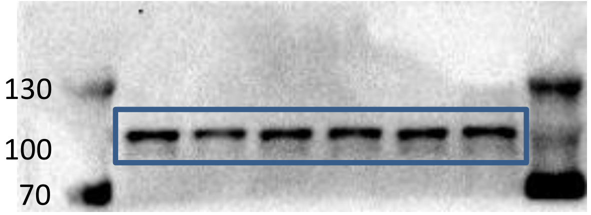

WB: pERK1/2

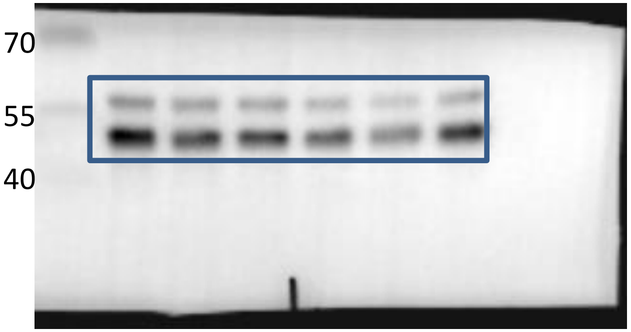

WB: ERK1/2

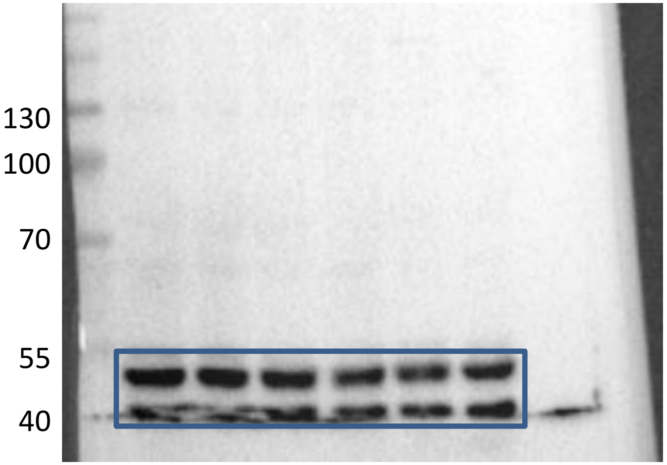

Appendix FigureS8 C1156Y

All the different proteins were detected on different blots without stripping.

WB: pAKT

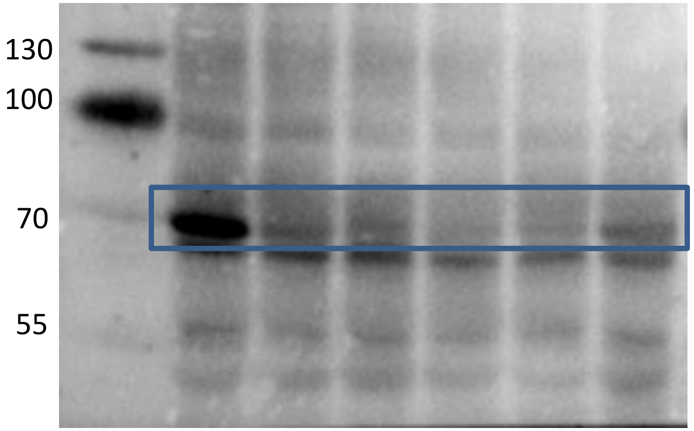

WB: AKT

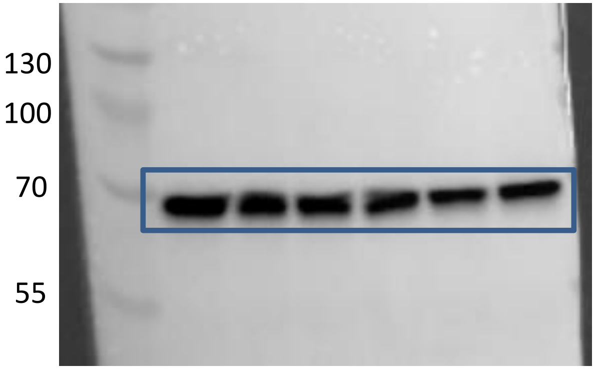

WB: β-actin

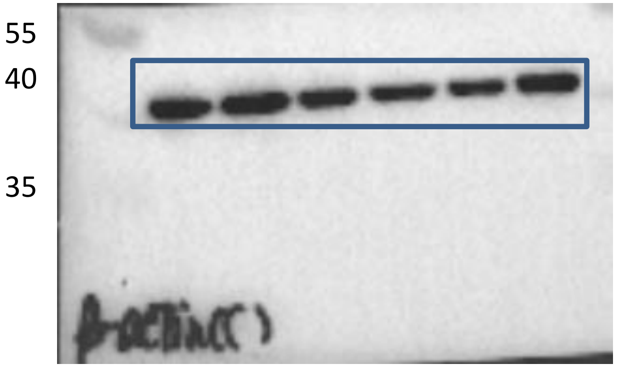

Appendix FigureS8 C1156Y

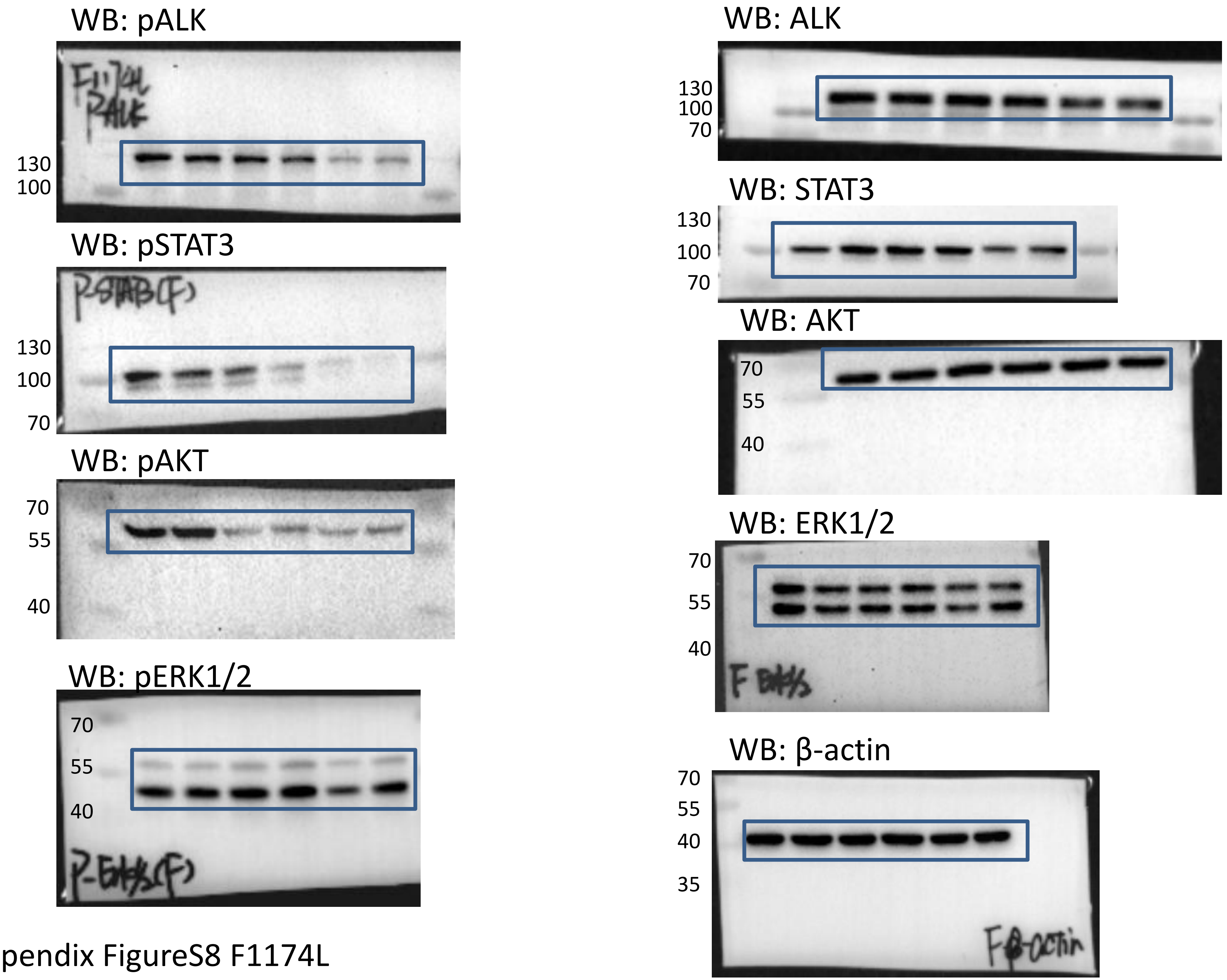

Appendix FigureS8 F1174L

All the different proteins were detected on different blots without stripping.
