# Supplementary material for: A new ALK inhibitor overcomes resistance to first‐ and second‐generation inhibitors in NSCLC
Source: EMBO Mol Med. 2021 Nov 30;14(1):e14296. doi: 10.15252/emmm.202114296 (PMC8749467; doi:10.15252/emmm.202114296)
Supplement: Supplementary file 2 — Source Data for Appendix [file EMMM-14-e14296-s005.zip › EMM-2021-14296-V3-FigureS9_Source_Data-sd.pdf]

HE: vehicle

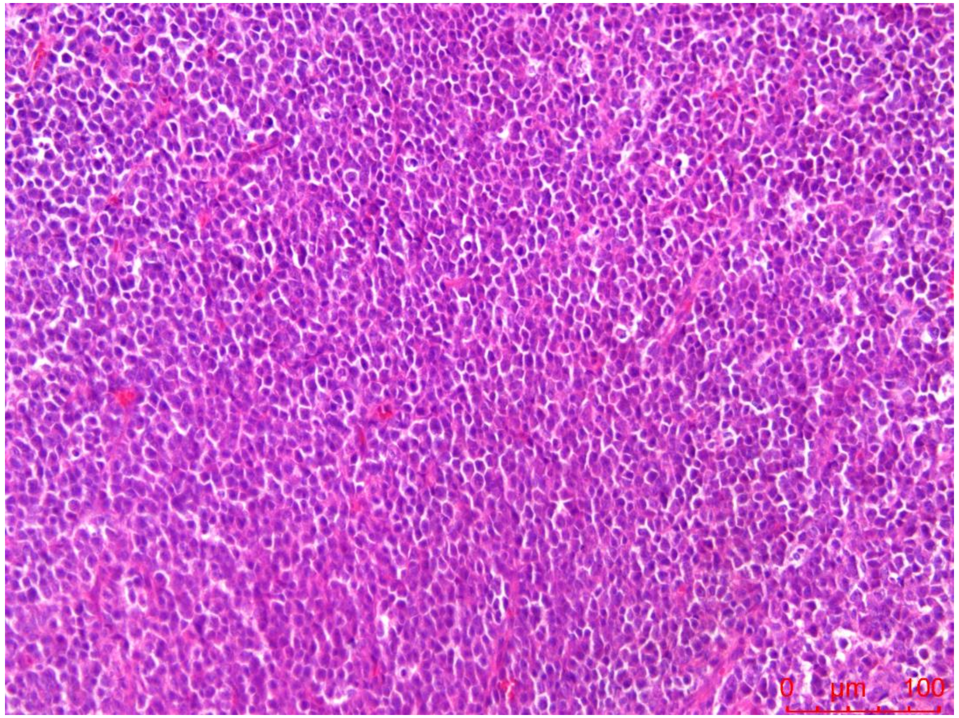

HE: 30 mg/kg

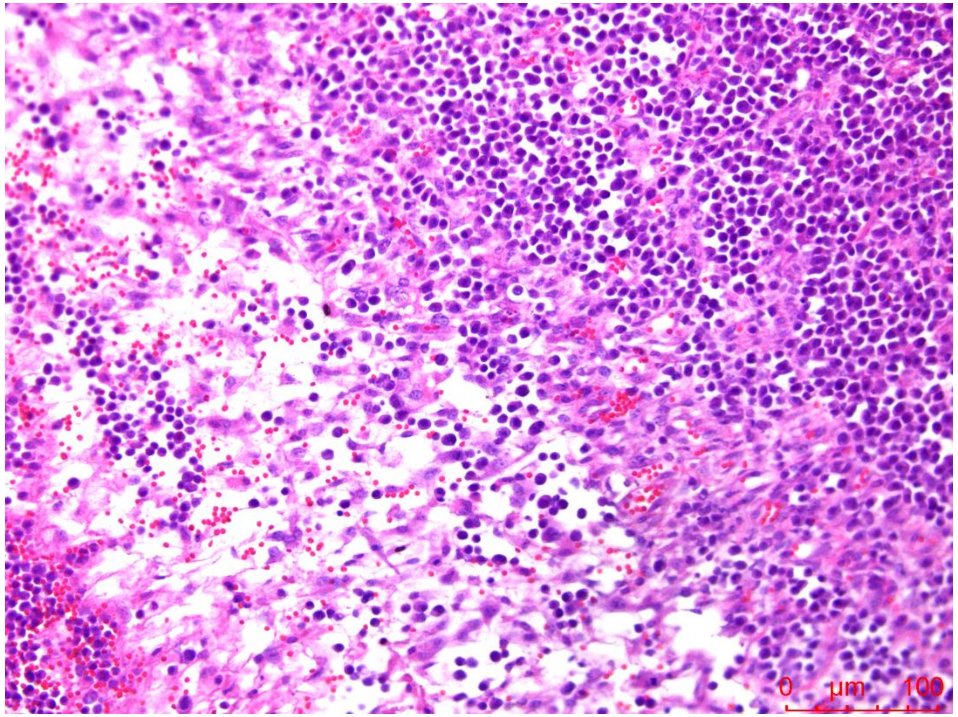

HE: 60 mg/kg

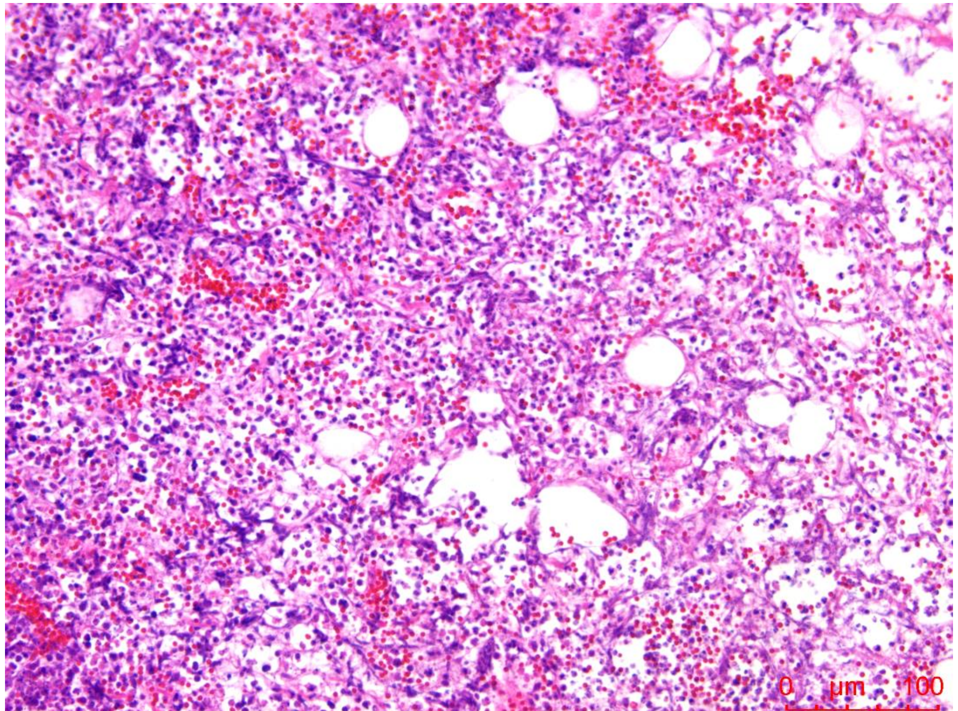

Appendix Figure S9A

pALK: vehicle

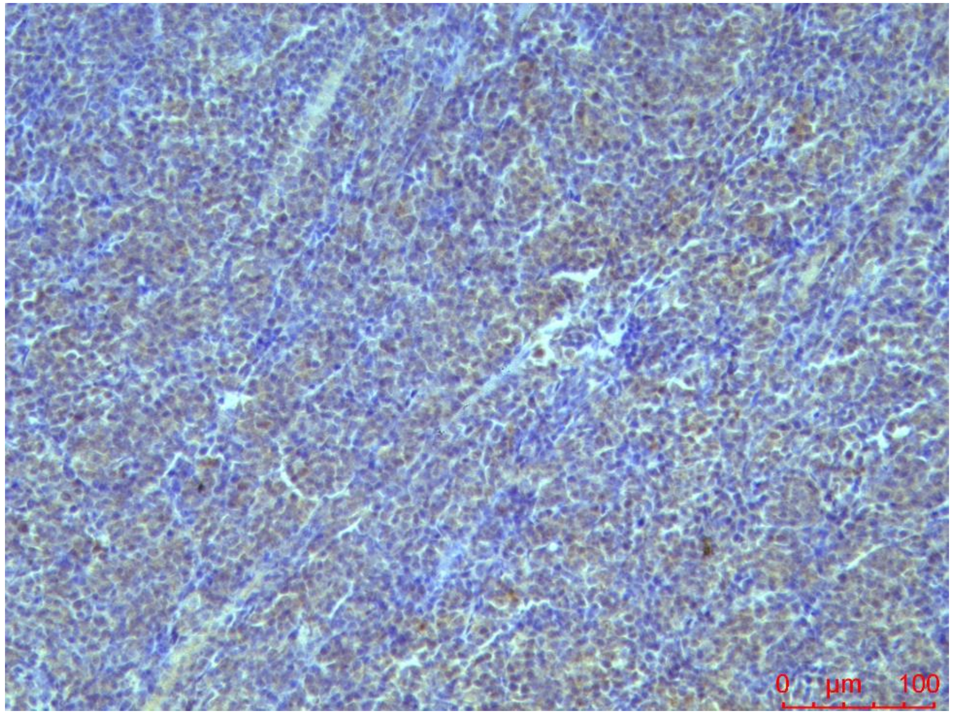

pALK: 30 mg/kg

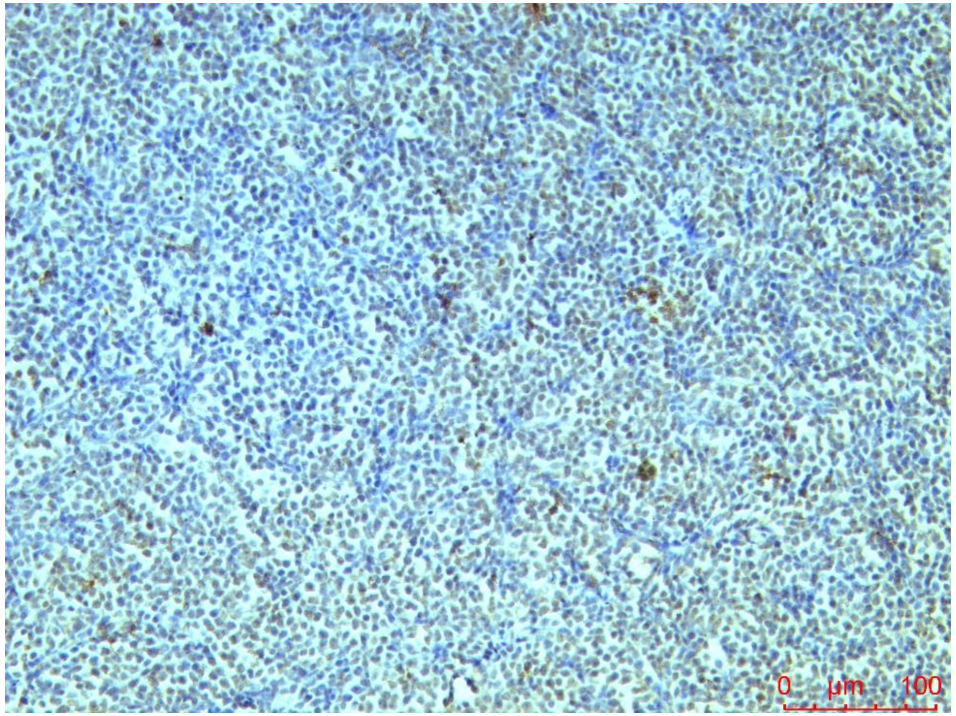

pALK: 60 mg/kg

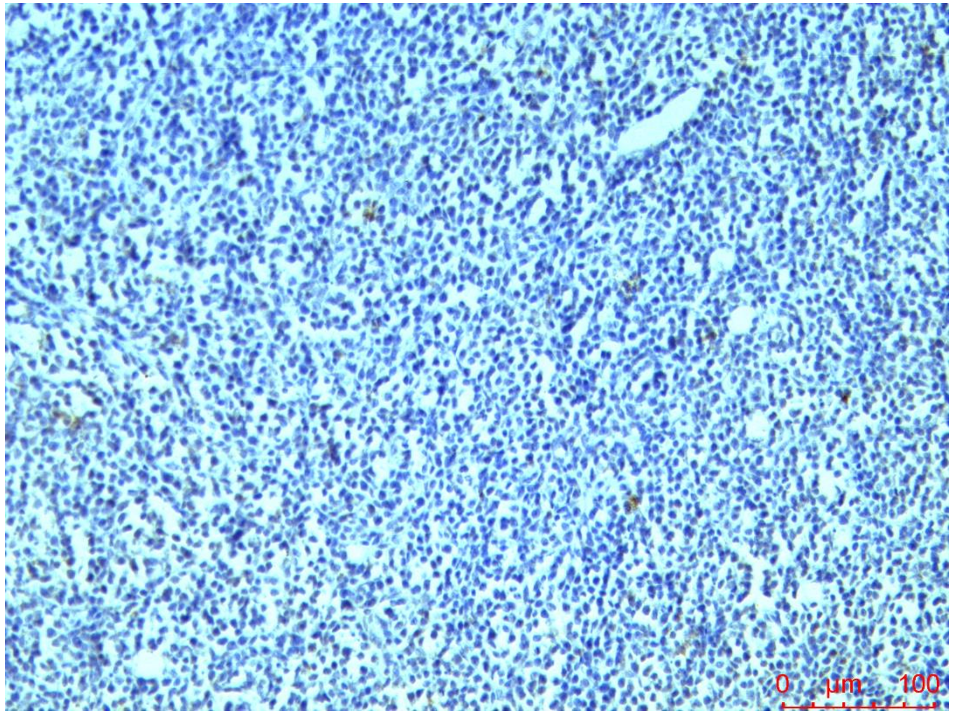

Appendix Figure S9A

cl-caspase3:
vehicle

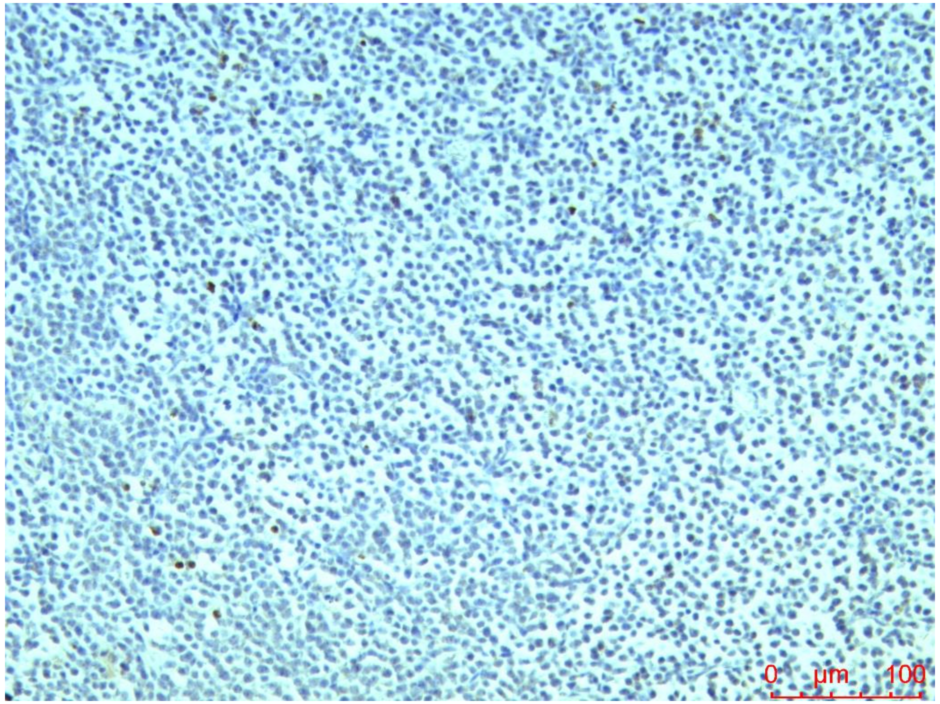

cl-caspase3:
30 mg/kg

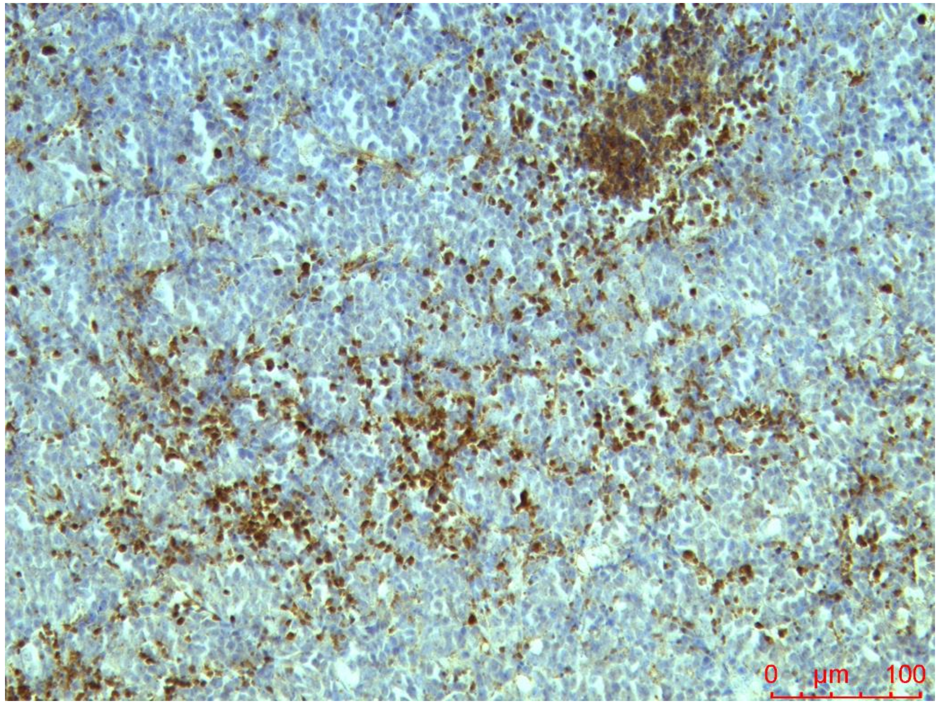

pALK:
60 mg/kg

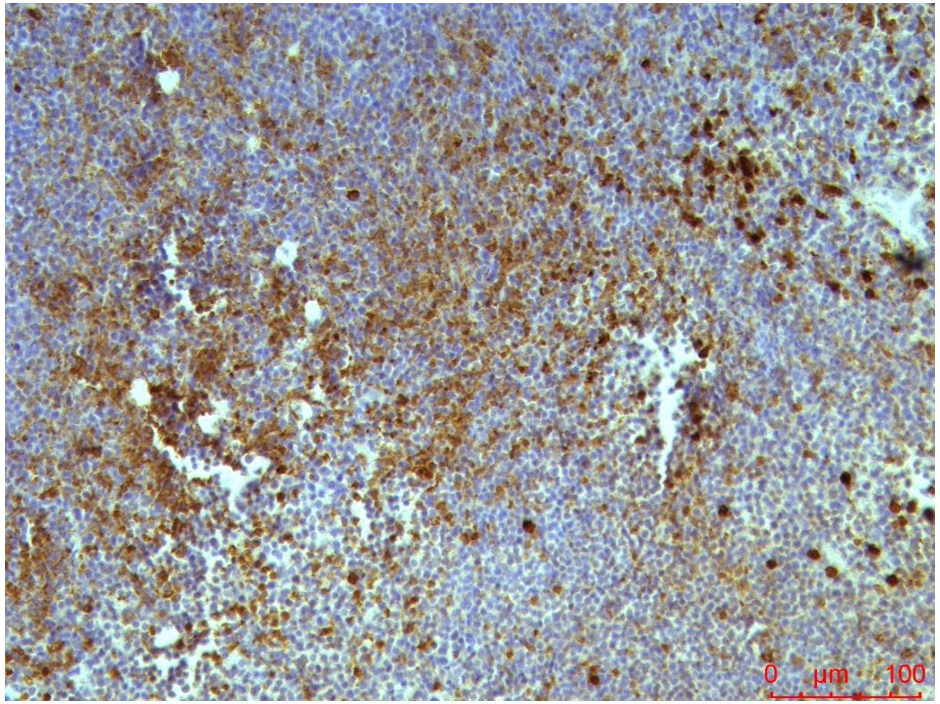

Appendix Figure S9A
