# Supplementary figures and images for: A new ALK inhibitor overcomes resistance to first‐ and second‐generation inhibitors in NSCLC
Source: EMBO Mol Med. 2021 Nov 30;14(1):e14296. doi: 10.15252/emmm.202114296 (PMC8749467; doi:10.15252/emmm.202114296)

WB: PARP

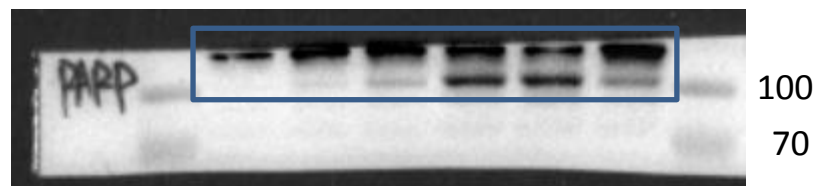

WB:  $\beta$ -actin

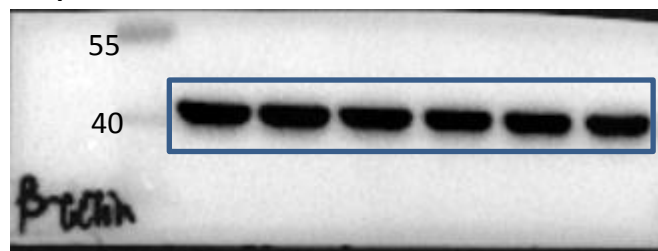

Figure Appendix S3A

Supplement: Supplementary file 2 — Source Data for Appendix [file EMMM-14-e14296-s005.zip › EMM-2021-14296-V3-FigureS3_Source_Data-sd.pdf]

WB: pALK

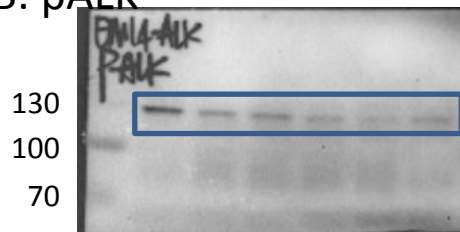

WB: ALK

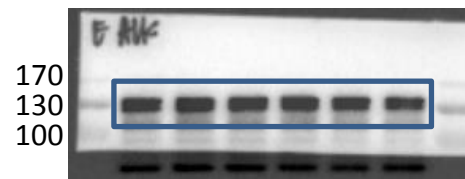

WB: pSTAT3

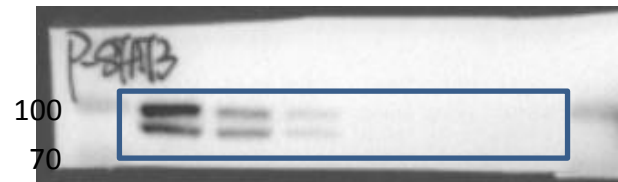

WB: STAT3

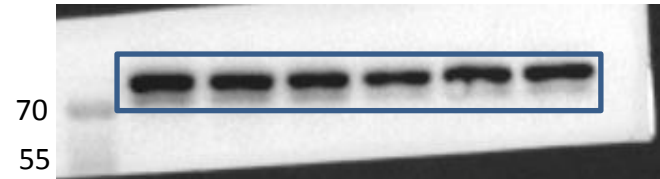

WB: pAKT

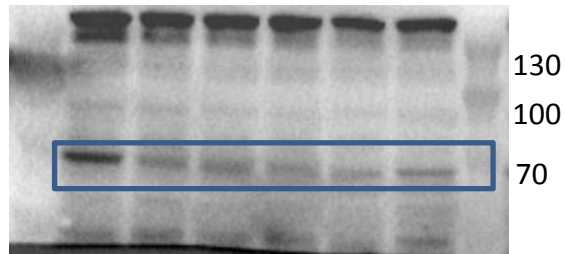

WB: AKT

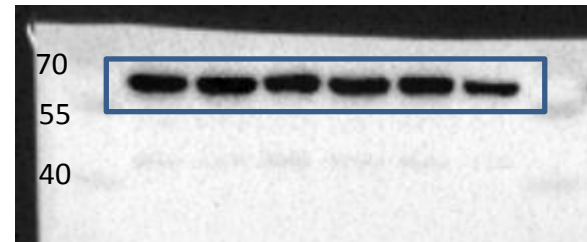

WB: pERK1/2

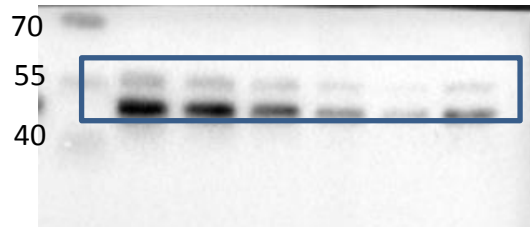

WB: ERK1/2

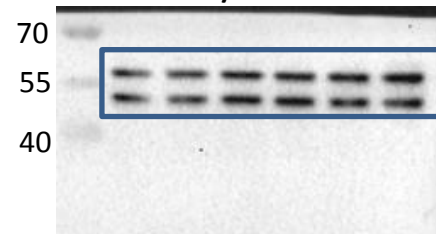

WB:  $\beta$ -actin

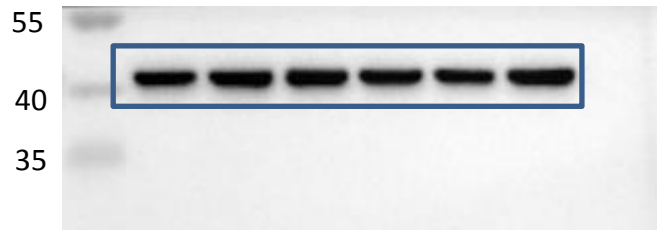

Figure1C

Supplement: Supplementary file 3 — Source Data for Figure 1 [file EMMM-14-e14296-s003.pdf]
