# Supplementary material for: A new ALK inhibitor overcomes resistance to first‐ and second‐generation inhibitors in NSCLC
Source: EMBO Mol Med. 2021 Nov 30;14(1):e14296. doi: 10.15252/emmm.202114296 (PMC8749467; doi:10.15252/emmm.202114296)
Supplement: Supplementary file 4 — Source Data for Figure 2 [file EMMM-14-e14296-s002.pdf]

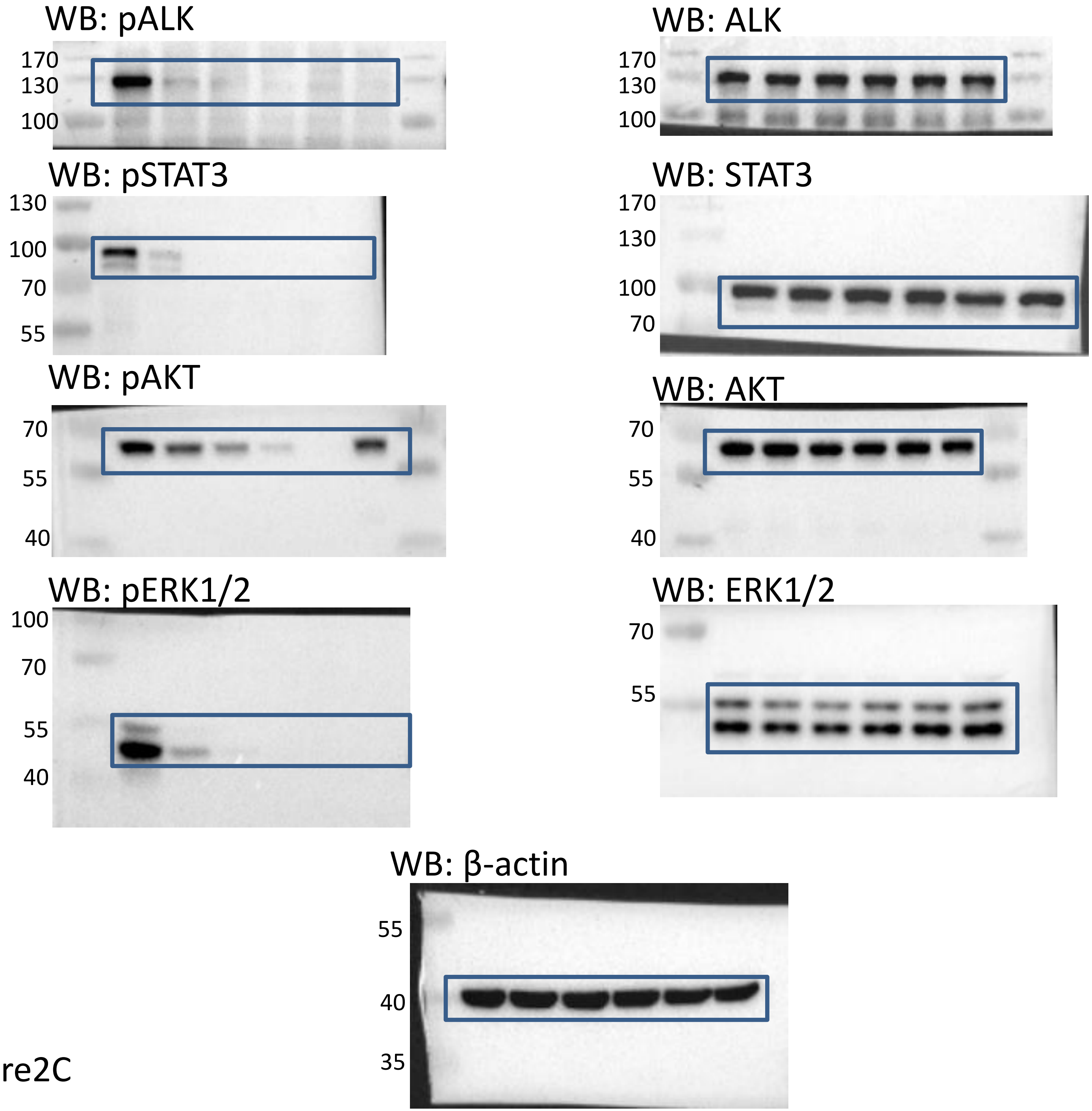
WB: pALK
170
130
100
WB: ALK
170
130
100
WB: pSTAT3
130
100
70
55
WB: STAT3
170
130
100
70
WB: pAKT
70
55
40
WB: AKT
70
55
40
WB: pERK1/2
100
70
55
40
WB: ERK1/2
70
55
WB: β-actin
55
40
35

Figure2C

HE: Vehicle

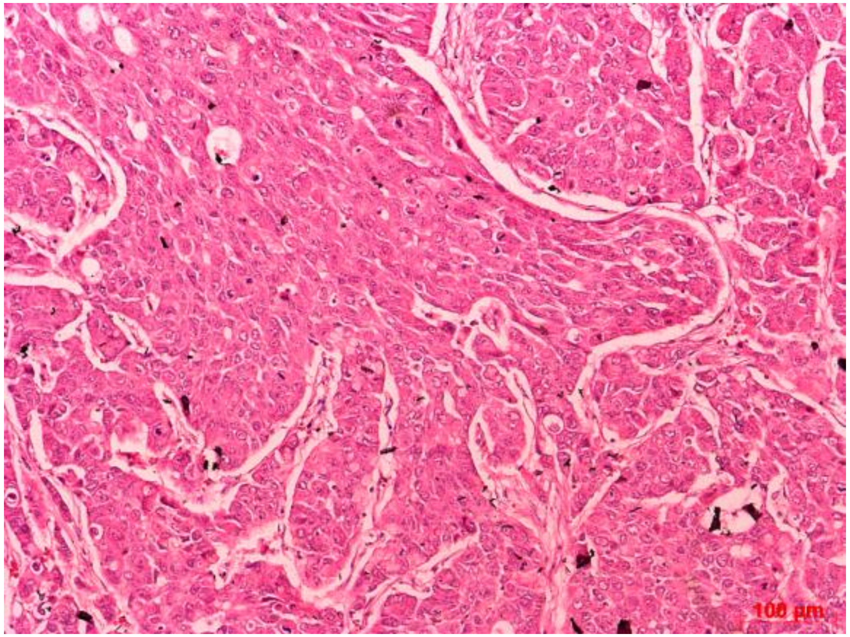

HE: 30 mg/kg

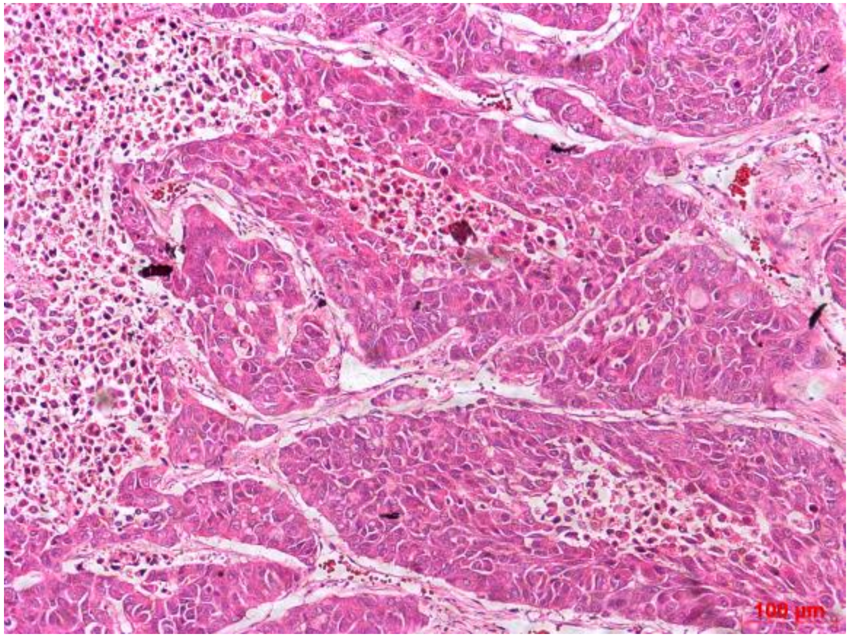

HE: 50 mg/kg

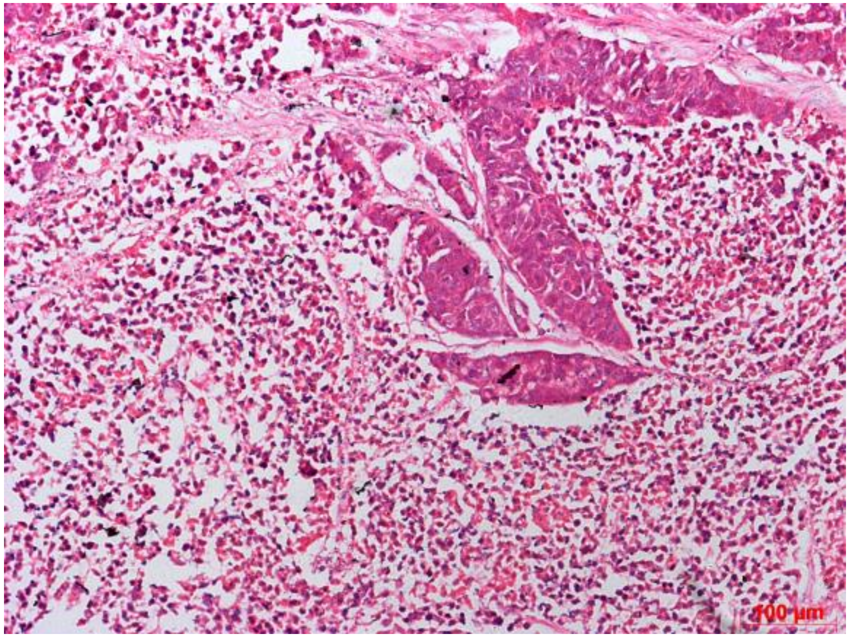

Figure2G

pALK: Vehicle

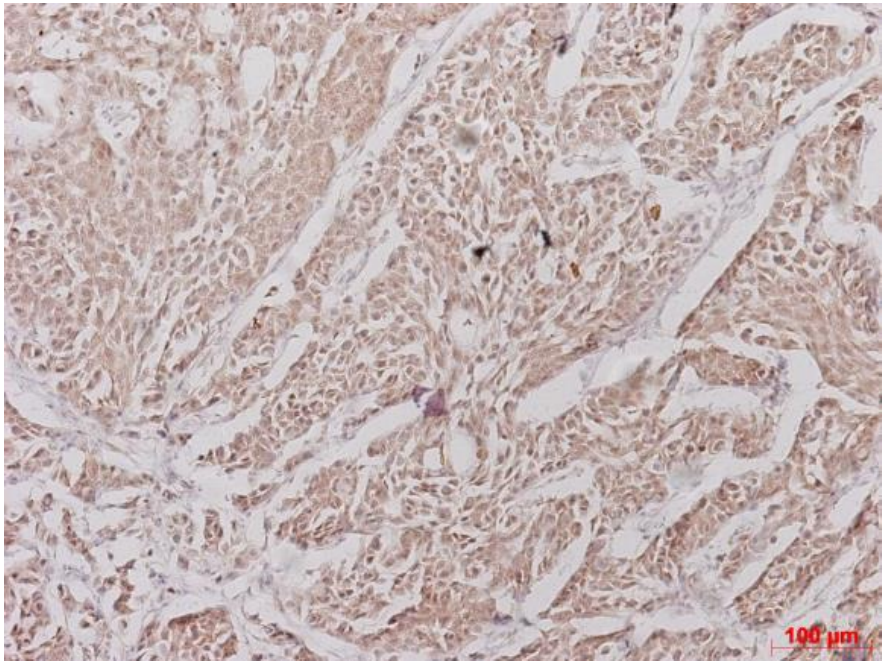

pALK: 30 mg/kg

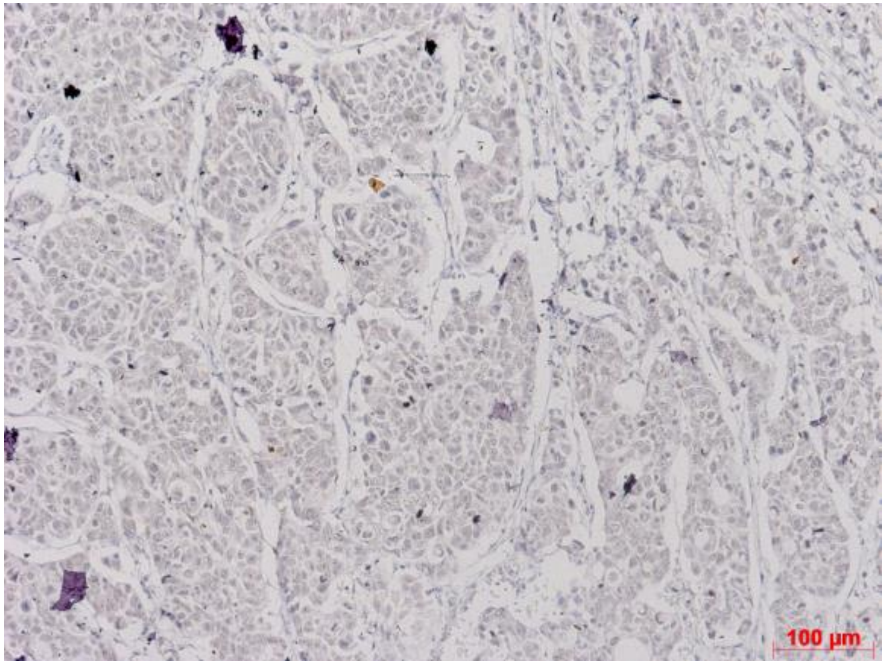

pALK: 50 mg/kg

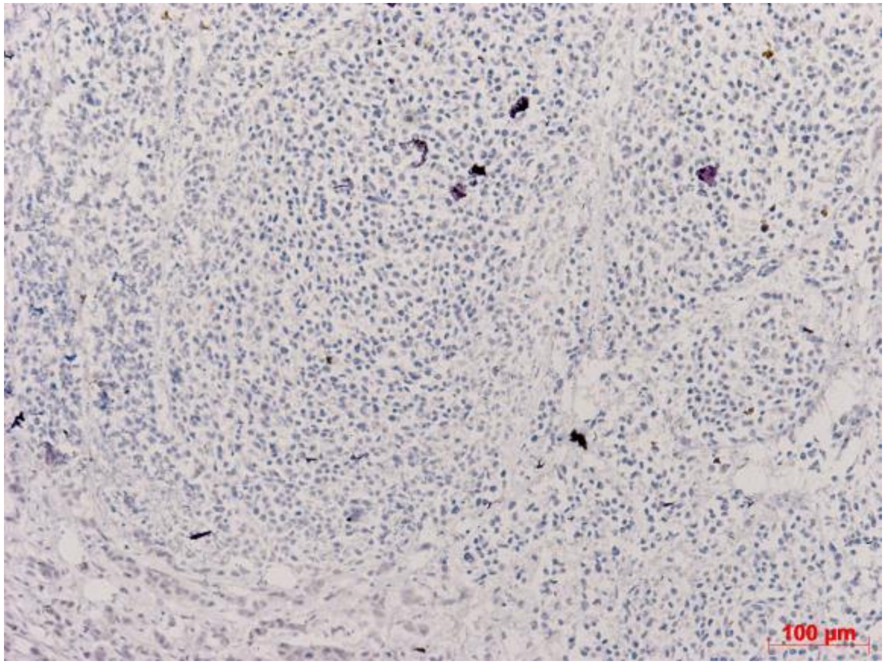

Figure2G

cl-caspase3: Vehicle

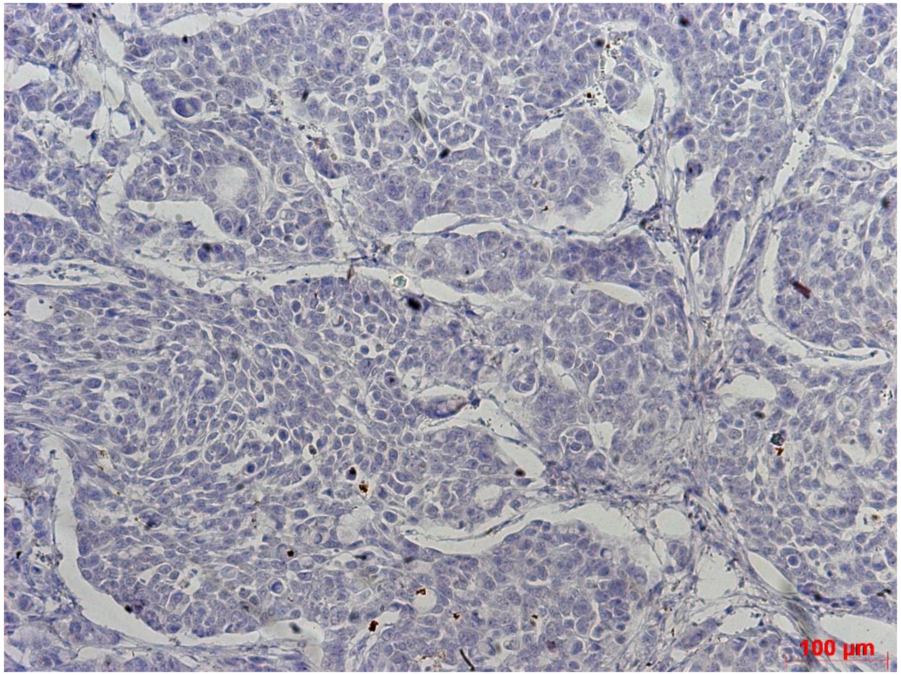

cl-caspase-3: 30 mg/kg

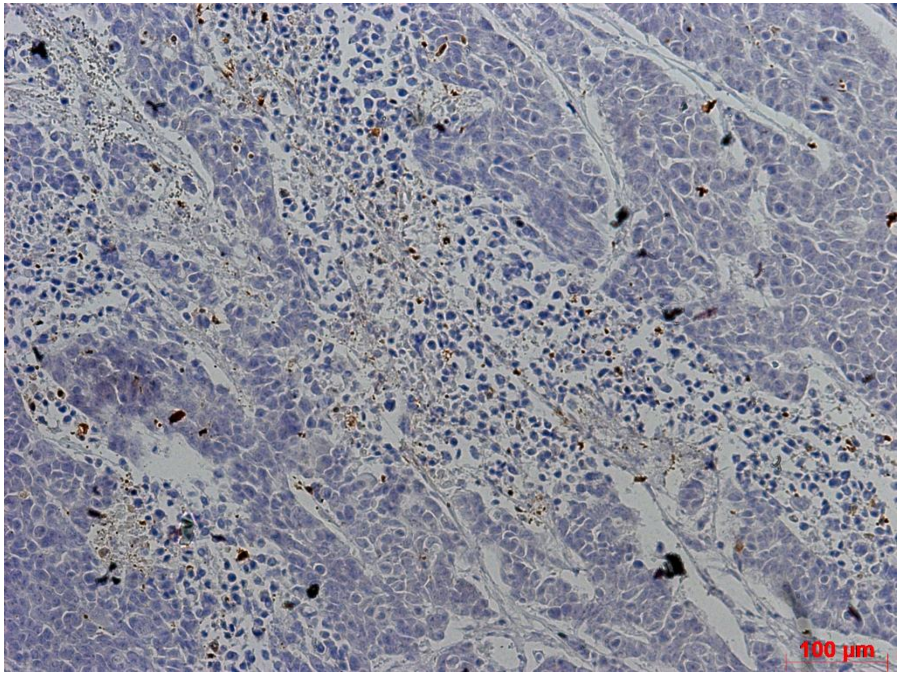

cl-caspase3: 50 mg/kg

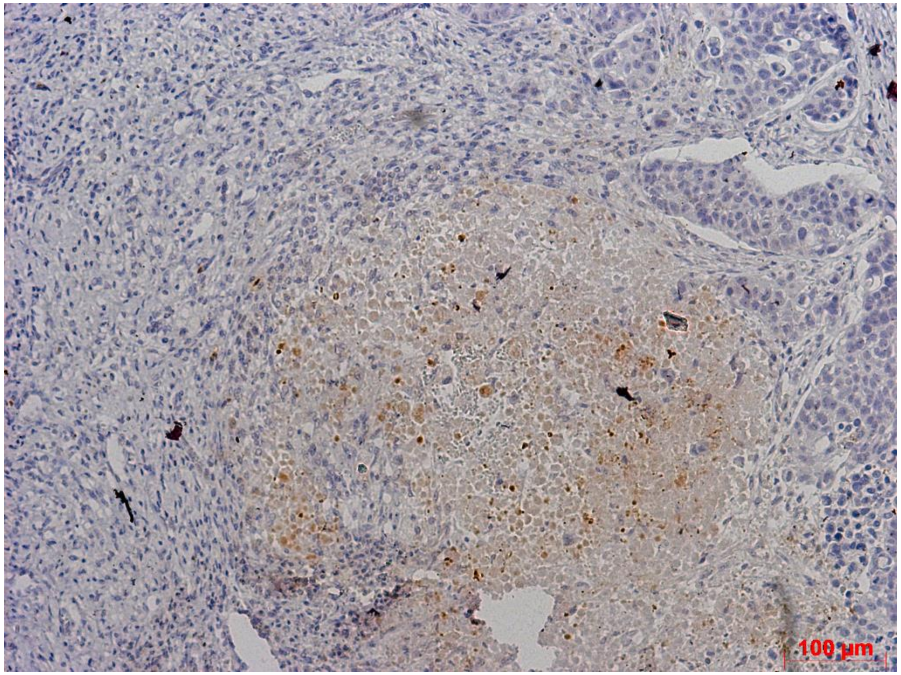

Figure2G
