# Supplementary material for: A new ALK inhibitor overcomes resistance to first‐ and second‐generation inhibitors in NSCLC
Source: EMBO Mol Med. 2021 Nov 30;14(1):e14296. doi: 10.15252/emmm.202114296 (PMC8749467; doi:10.15252/emmm.202114296)
Supplement: Supplementary file 5 — Source Data for Figure 3 [file EMMM-14-e14296-s006.pdf]

Figure5A

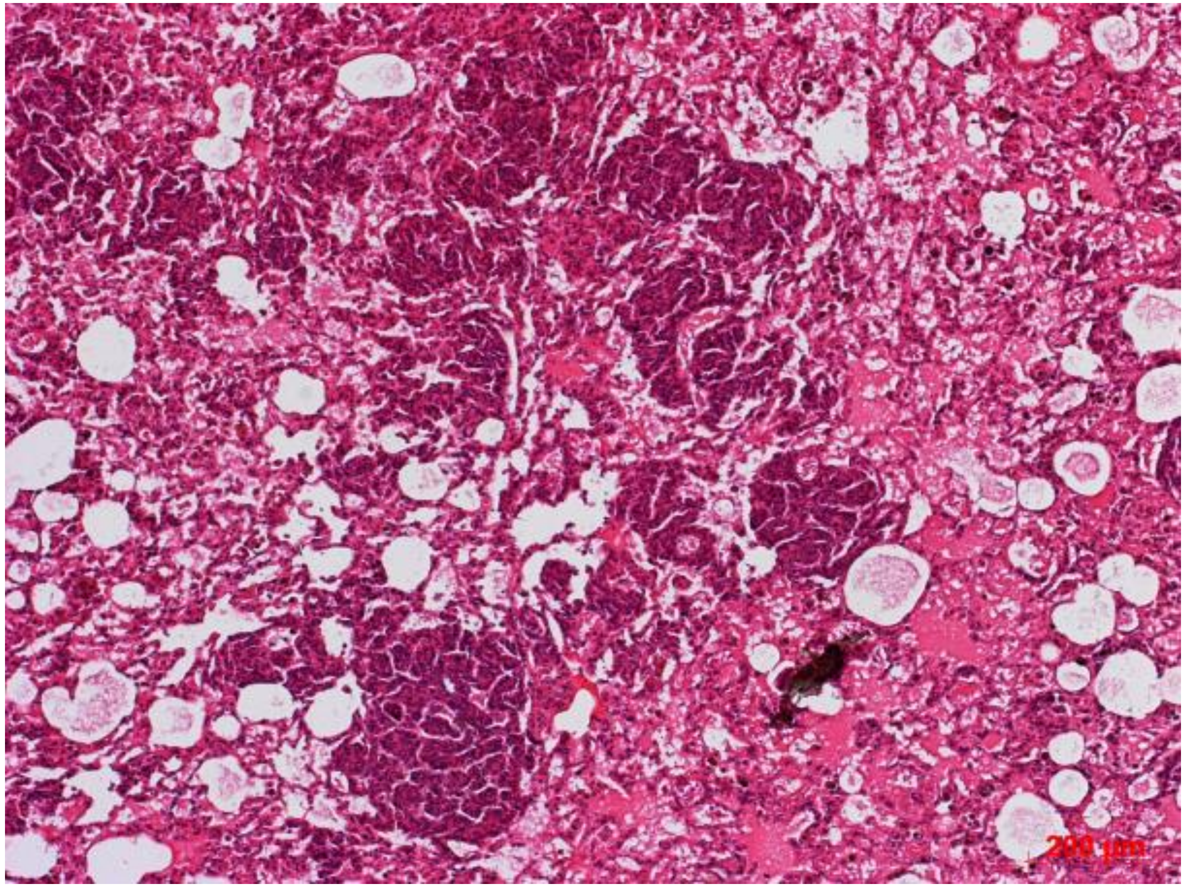

HE: Before treatment

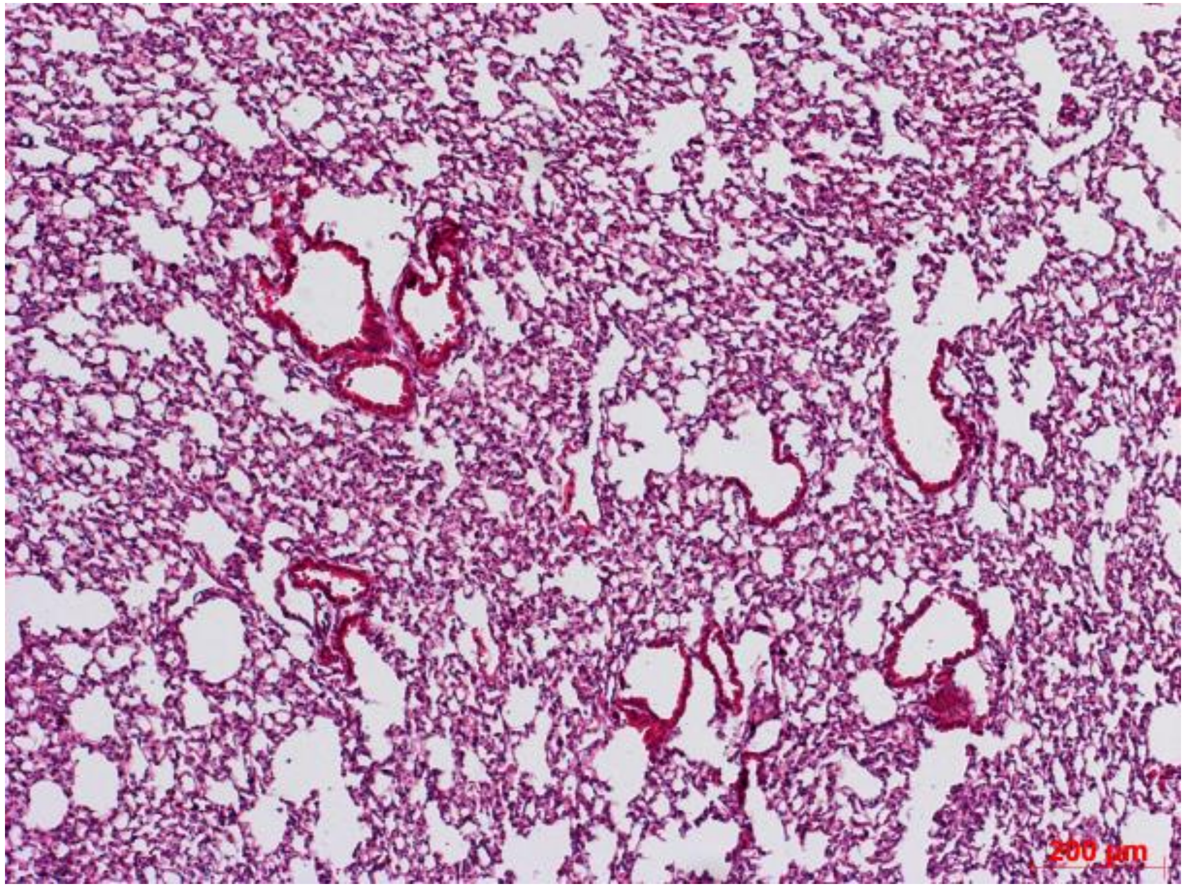

HE: 3 weeks treatment

Figure5B

cl-caspase3:
Vehicle

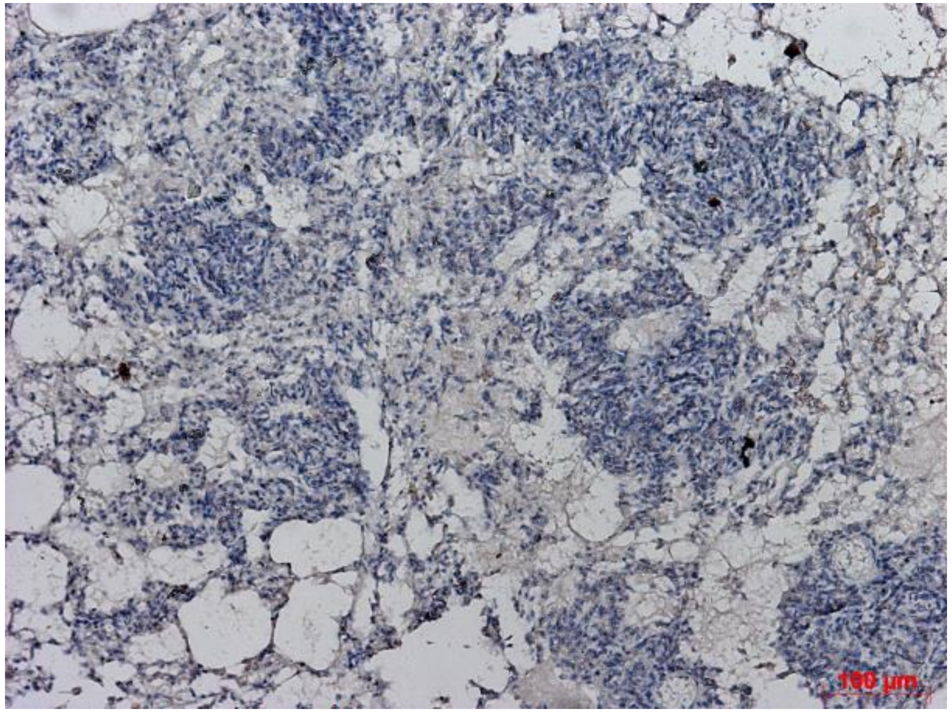

pALK:
Vehicle

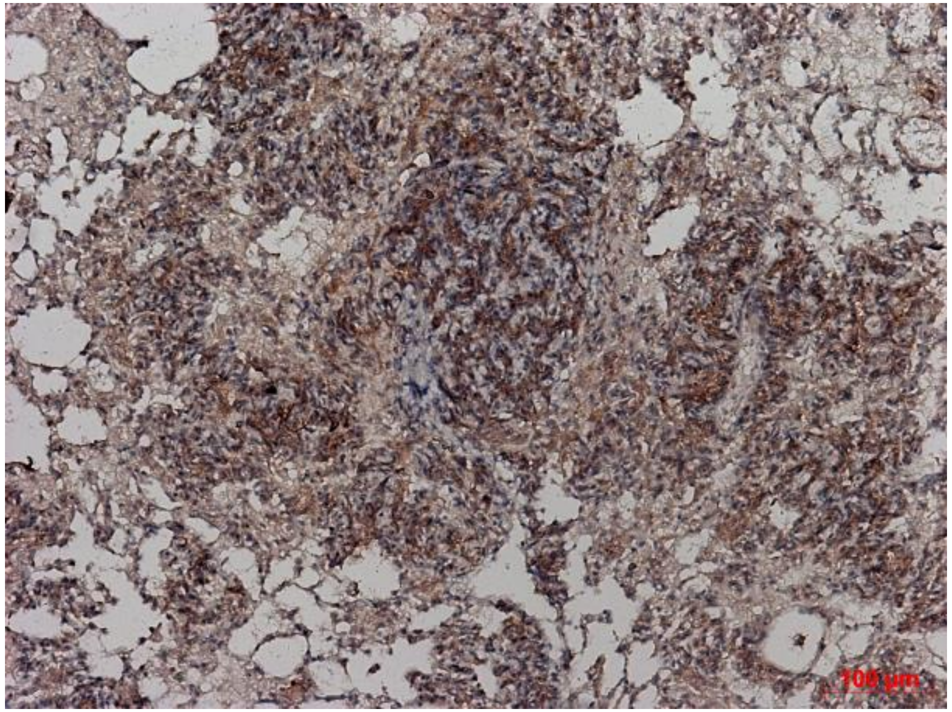

cl-caspase3:
XMU-MP-5

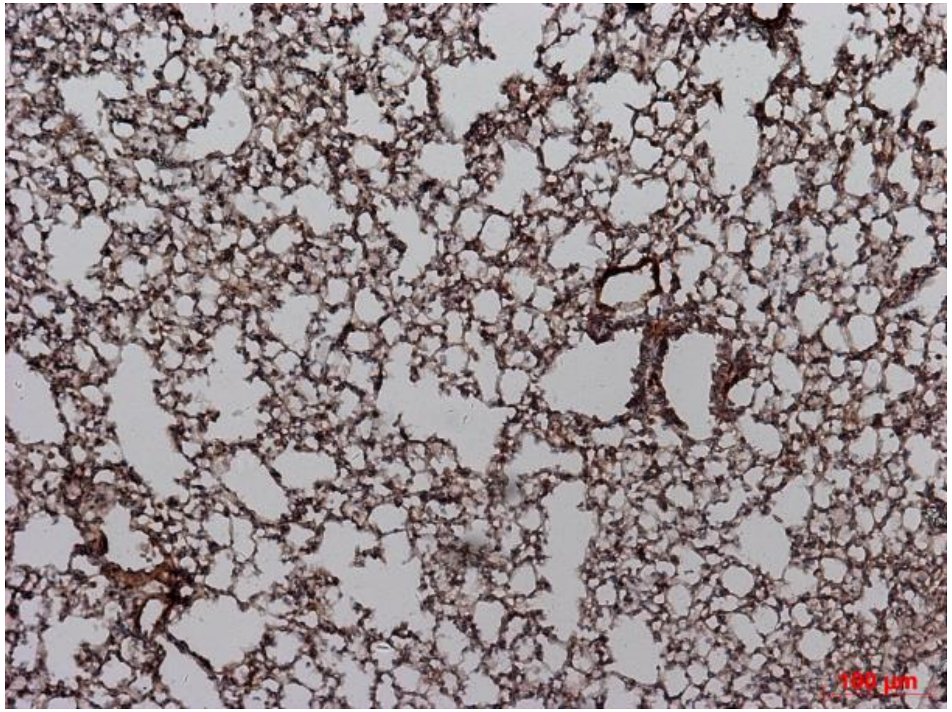

pALK:
XMU-MP-5

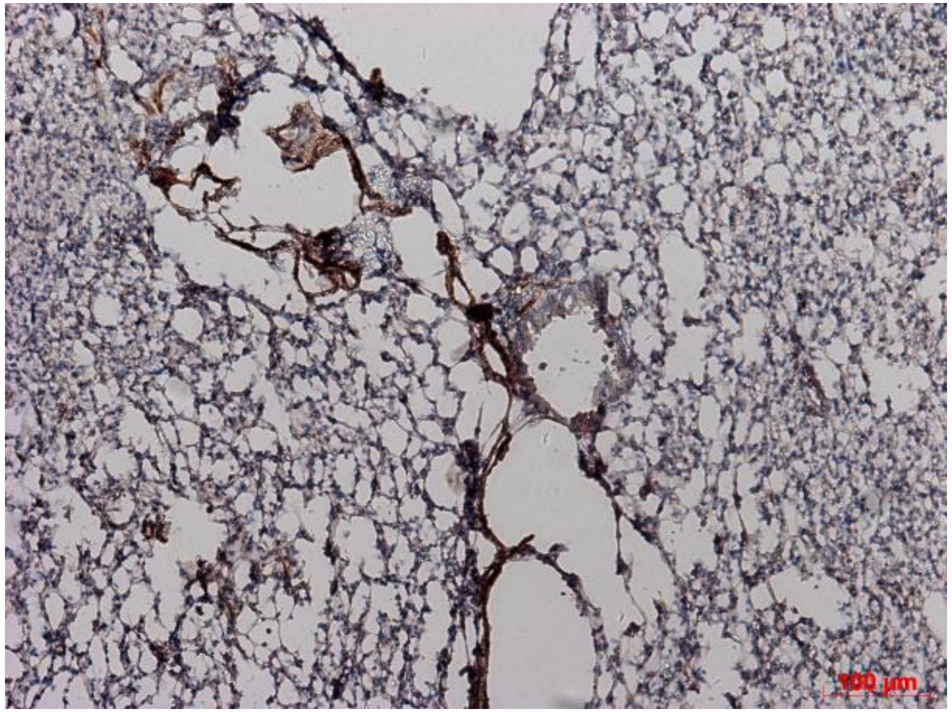

Figure5C

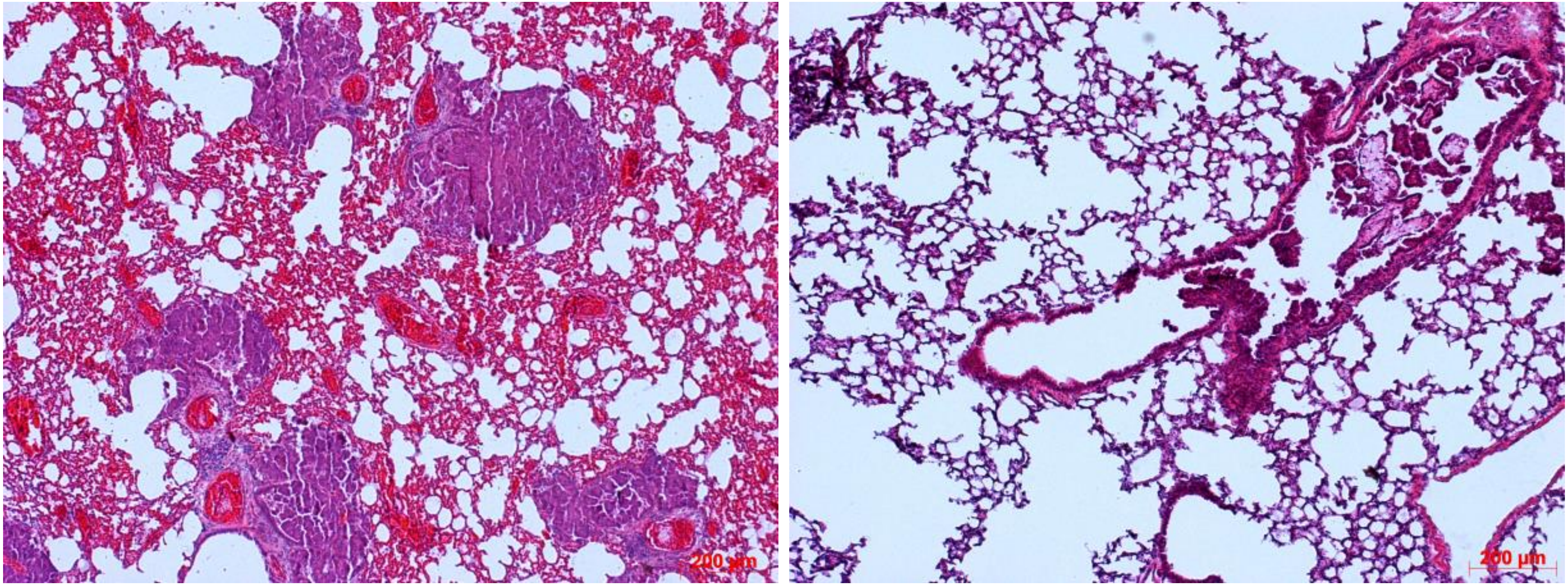

HE: Before treatment

HE: 1 week treatment

Figure5D

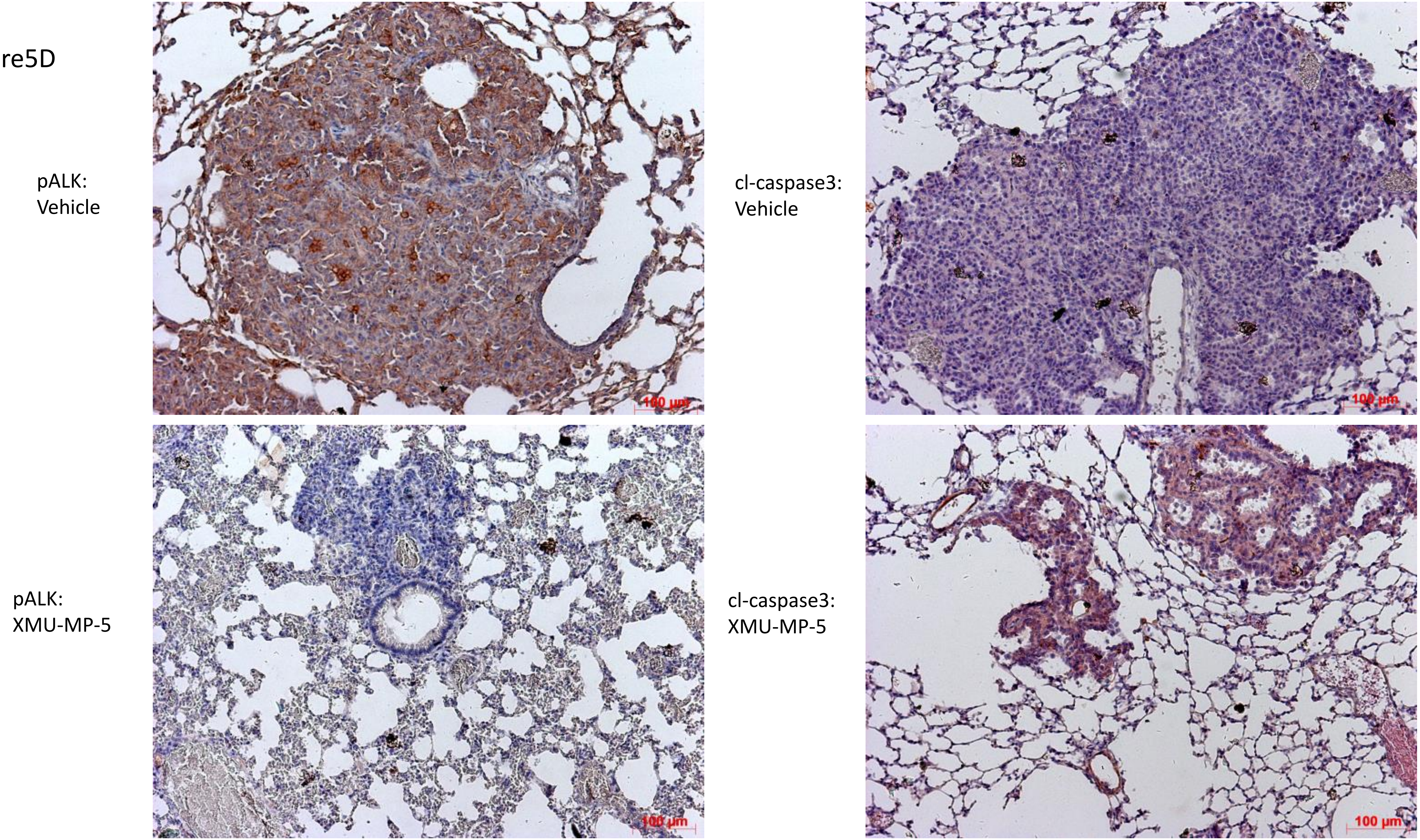

pALK:
Vehicle
cl-caspase3:
Vehicle
pALK:
XMU-MP-5
cl-caspase3:
XMU-MP-5
100 μm
100 μm
100 μm
100 μm
