# Supplementary material for: A new ALK inhibitor overcomes resistance to first‐ and second‐generation inhibitors in NSCLC
Source: EMBO Mol Med. 2021 Nov 30;14(1):e14296. doi: 10.15252/emmm.202114296 (PMC8749467; doi:10.15252/emmm.202114296)
Supplement: Supplementary file 6 [file EMMM-14-e14296-s004.pdf]

EMBO Molecular Medicine

A new ALK inhibitor overcomes resistance to first- and second-generation inhibitors in NSCLC

Yue Lu, zhenzhen fan, Su-Jie Zhu, Xiaoxing Huang, Zhongji Zhuang, Yunzhan Li, Zhou Deng, Lei Gao, Xuehui Hong, Ting Zhang, Li Li, Xihuan Sun, Wei Huang, Jingfang Zhang, Yan Liu, Baoding Zhang, Jie Jiang, Fu Gui, Zheng Wang, Qiyuan Li, Siyang Song, Xin Huang, Qiao Wu, Lanfen Chen, Dawang Zhou, Jianming Zhang, Cai-Hong Yun, Liang Chen, and Xianming Deng

DOI: 10.15252/emmm.202114296

Corresponding authors: Yue Lu (dandansmiling@126.com) , Liang Chen (chenliang@jnu.edu.cn), Jianming Zhang (jzuc@shsmu.edu.cn), Cai-Hong Yun (yunch@bjmu.edu.cn)

Review Timeline:

Submission Date:	19th Mar 21
Editorial Decision:	14th Apr 21
Revision Received:	12th Sep 21
Editorial Decision:	11th Oct 21
Revision Received:	29th Oct 21
Accepted:	3rd Nov 21

Editor: Lise Roth

Transaction Report:

(Note: With the exception of the correction of typographical or spelling errors that could be a source of ambiguity, letters and reports are not edited. Depending on transfer agreements, referee reports obtained elsewhere may or may not be included in this compilation. Referee reports are anonymous unless the Referee chooses to sign their reports.)

14th Apr 2021

Dear Prof. Deng,

Thank you for the submission of your manuscript to EMBO Molecular Medicine. We have now received feedback from the three reviewers who agreed to evaluate your manuscript. As you will see from the reports below, the referees acknowledge the interest of the study and are overall supporting publication of your work pending appropriate revisions.

Addressing the reviewers' concerns in full will be necessary for further considering the manuscript in our journal, and acceptance of the manuscript will entail a second round of review. EMBO Molecular Medicine encourages a single round of revision only and therefore, acceptance or rejection of the manuscript will depend on the completeness of your responses included in the next, final version of the manuscript. For this reason, and to save you from any frustrations in the end, I would strongly advise against returning an incomplete revision.

When submitting your revised manuscript, please carefully review the instructions that follow below. Failure to include requested items will delay the evaluation of your revision:

1) A .docx formatted version of the manuscript text (including legends for main figures, EV figures and tables). Please make sure that the changes are highlighted to be clearly visible.

2) Individual production quality figure files as .eps, .tif, .jpg (one file per figure).

3) A .docx formatted letter INCLUDING the reviewers' reports and your detailed point-by-point responses to their comments. As part of the EMBO Press transparent editorial process, the point-by-point response is part of the Review Process File (RPF), which will be published alongside your paper.

4) A complete author checklist, which you can download from our author guidelines (https://www.embopress.org/page/journal/17574684/authorguide#submissionofrevisions). Please insert information in the checklist that is also reflected in the manuscript. The completed author checklist will also be part of the RPF.

5) Please note that all corresponding authors are required to supply an ORCID ID for their name upon submission of a revised manuscript.

6) Before submitting your revision, primary datasets produced in this study need to be deposited in an appropriate public database (see https://www.embopress.org/page/journal/17574684/authorguide#dataavailability).
Please remember to provide a reviewer password if the datasets are not yet public.
The accession numbers and database should be listed in a formal "Data Availability " section (placed after Materials & Method).
Please note that the Data Availability Section is restricted to new primary data that are part of this study.

*** Note - All links should resolve to a page where the data can be accessed. ***

7) We would also encourage you to include the source data for figure panels that show essential data. Numerical data should be provided as individual .xls or .csv files (including a tab describing the data). For blots or microscopy, uncropped images should be submitted (using a zip archive if multiple images need to be supplied for one panel). Additional information on source data and instruction on how to label the files are available at
.

8) Our journal encourages inclusion of *data citations in the reference list* to directly cite datasets that were re-used and obtained from public databases. Data citations in the article text are distinct from normal bibliographical citations and should directly link to the database records from which the data can be accessed. In the main text, data citations are formatted as follows: "Data ref: Smith et al, 2001" or "Data ref: NCBI Sequence Read Archive PRJNA342805, 2017". In the Reference list, data citations must be labeled with "[DATASET]". A data reference must provide the database name, accession number/identifiers and a resolvable link to the landing page from which the data can be accessed at the end of the reference. Further instructions are available at .

9) We replaced Supplementary Information with Expanded View (EV) Figures and Tables that are collapsible/expandable online. A maximum of 5 EV Figures can be typeset. EV Figures should be cited as 'Figure EV1, Figure EV2" etc... in the text and their respective legends should be included in the main text after the legends of regular figures.

- For the figures that you do NOT wish to display as Expanded View figures, they should be bundled together with their legends in a single PDF file called *Appendix*, which should start with a short Table of Content. Appendix figures should be referred to in the main text as: "Appendix Figure S1, Appendix Figure S2" etc.

- Additional Tables/Datasets should be labeled and referred to as Table EV1, Dataset EV1, etc. Legends have to be provided in a separate tab in case of .xls files. Alternatively, the legend can be supplied as a separate text file (README) and zipped together with the Table/Dataset file.
See detailed instructions here:
.

10) For more information: There is space at the end of each article to list relevant web links for further consultation by our readers. Could you identify some relevant ones and provide such information as well? Some examples are patient associations, relevant databases, OMIM/proteins/genes links, author's websites, etc...

11) Every published paper now includes a 'Synopsis' to further enhance discoverability. Synopses are displayed on the journal webpage and are freely accessible to all readers. They include a short stand first (maximum of 300 characters, including space) as well as 2-5 one-sentences bullet points that summarizes the paper. Please write the bullet points to summarize the key NEW findings. They should be designed to be complementary to the abstract - i.e. not repeat the same text. We encourage inclusion of key acronyms and quantitative information (maximum of 30 words / bullet point). Please use the passive voice. Please attach these in a separate file or send them by email, we will incorporate them accordingly.

Please also suggest a striking image or visual abstract to illustrate your article. If you do please provide a png file 550 px-wide x 400-px high.

12) As part of the EMBO Publications transparent editorial process initiative (see our Editorial at http://embomolmed.embopress.org/content/2/9/329), EMBO Molecular Medicine will publish online a Review Process File (RPF) to accompany accepted manuscripts.
In the event of acceptance, this file will be published in conjunction with your paper and will include the anonymous referee reports, your point-by-point response and all pertinent correspondence relating to the manuscript. Let us know whether you agree with the publication of the RPF and as here, if you want to remove or not any figures from it prior to publication.
Please note that the Authors checklist will be published at the end of the RPF.

EMBO Molecular Medicine has a "scooping protection" policy, whereby similar findings that are published by others during review or revision are not a criterion for rejection. Should you decide to submit a revised version, I do ask that you get in touch after three months if you have not completed it, to update us on the status.

I look forward to receiving your revised manuscript.

Yours sincerely,

Lise Roth

Lise Roth, PhD
Editor
EMBO Molecular Medicine

To submit your manuscript, please follow this link:

Link Not Available

*Additional important information regarding Figures

Each figure should be given in a separate file and should have the following resolution:
Graphs 800-1,200 DPI
Photos 400-800 DPI
Colour (only CMYK) 300-400 DPI"

Figures are not edited by the production team. All lettering should be the same size and style; figure panels should be indicated by capital letters (A, B, C etc). Gridlines are not allowed except for log plots. Figures should be numbered in the order of their appearance in the text with Arabic numerals. Each Figure must have a separate legend and a caption is needed for each panel.

*Additional important information regarding figures and illustrations can be found at https://bit.ly/EMBOPressFigurePreparationGuideline

***** Reviewer's comments *****

Referee #1 (Comments on Novelty/Model System for Author):

The systems are adequate.

Referee #1 (Remarks for Author):

The manuscript by Lu et al., describes the discovery and development of a novel inhibitor of ALK, which can eliminate cells that are resistant to earlier generations of ALK inhibitors. The novel compound, named XMU-MP-5, could induce apoptosis only in cells expressing mutant ALK and could prevent the growth of ALK-dependent lung tumors in mouse xenograft models as well as GEMM models. Overall, the studies are novel and interesting.

A significant amount of data is presented in this manuscript; they are generally of high quality. Most of the expected experiments have been conducted; these include basic cell biology studies to measure proliferation and apoptosis, biochemical examination of the signaling pathways affected by this compound, determination of the selectivity and specificity, assessment of PK/PD values, in vivo efficacy in mouse models etc. Overall, the study is interesting and novel.

There are two aspects that need attention. First, the data in Supplementary Table 1 shows the efficacy of the identified compound against certain kinases, especially mutant EGFR. It would be interesting and relevant to assess if XMU-MP-5 has comparable efficacy against cell lines harboring standard EGFR mutations like Exon 19 deletion. Further, it appears to be effective against EGFR T790M mutation as well. It would be worthwhile to check if the compound is active against cell lines like H1975 which harbor this gatekeeper mutation.

Secondly, the data in Supplementary Figure 3 is not clear. There is a fourth panel in the Figure, perhaps a panel D, which is not labeled and not mentioned in the legend. This needs to be examined.

Given the potential to translate this drug into the clinic, it would be worthwhile to examine if XMU-MP-5 affects the viability of primary human lung epithelial cells (small-airway epithelial cells, normal human bronchial epithelial cells etc.) and if a therapeutic window is achievable. The effect of this agent on human T cells and B cells would also be interesting to establish. Overall, this is an interesting manuscript that describes a compound that can potentially be translated to the clinic.

Referee #2 (Remarks for Author):

The authors of this manuscript have synthesized and characterized a novel ALK tyrosine kinase inhibitor called XMU-MP-5. They tested this compound in vitro on ALK-positive and negative cancer cell lines and in vivo on ALK-positive xenograft and genetically engineered mouse models. They also performed crystallography to visualize XMU-MP-5 in complex with the ALK kinase domain as well as modeling to predict structural changes that occur within the kinase domain mutations that may affect earlier generation TKIs but not XMU-MP-5. While there are significant revisions that are needed, both in terms of readability, accuracy of claims, and addition of information to the manuscript, this work represents is a novel contribution to the field of targeted therapy. The agent described in this paper can overcome all the known mutations that confer resistance to 1st and 2nd generation ALK TKIs and therefore can be considered a next-generation TKI. I believe this manuscript would be a good fit for EMBO Molecular Medicine contingent on a major revision - with close attention to detail for factual error, grammar/spelling, copy-editing, and interpretation of data.

Introduction
1. The biggest flaw in this section is the complete ignorance of lorlatinib, which is also able to overcome L1196M and G1202R in vivo; this must be added to the introduction before it can be published.
Results
2. Figure 1
o May be helpful to show full chemical synthesis process in a Supplemental figure (optional)
o Confused as to how you get an IC50 value for XMU-MP-5 in Fig. 1B when the dose response curve does not extend to 50% cell viability. Recommend that this experiment be repeated with lower doses that achieve a sigmoidal curve in order to to

properly capture and illustrate the IC50.
3. Figure 4
o The emphasis on raw IC50 values between drugs is misleading. Greater potency does not necessarily mean more efficacy especially without any analysis of therapeutic index. The better way to compare drugs is to compare fold-change IC50 values of mutant to WT. The raw data in appendix table S4 is good to have alongside this.
o The sentence "XMU-MP-5 showed better activity than lorlatinib with IC50 values of 49 nM and 90 nM, respectively" is missing information about what mutants those IC50 values refer to (G1202R). This statement, while mathematically true, is not meaningful in an in vitro or in vivo sense and is frankly misleading: both XMU-MP-5 and lorlatinib exhibit >20 fold increase in G1202R IC50 values and yet both have demonstrated anti-tumor efficacy in vivo as shown in this manuscript and in the Zou et al. ALK lorlatinib manuscript from 2015.
o In panel C (Figure 4), inhibitor dosing is for 7 days only. That seems like a very short duration for demonstrating durable control over ALK L1196M mutant tumors. Indeed, at both doses tested (30 and 60 mg/kg), there is still tumor growth, albeit attenuated as compared to vehicle.
o Lorlatinib should have been used in the in vivo experiments to serve as a benchmark considering that it is also a 3rd generation TKI that 1) has already been FDA-approved and that 2) likely has a similar level of efficacy against the same mutations that the authors claim XMU-MP-5 targets.
Discussion
4. Discussion needs citations (i.e., when saying that "secondary mutations account for 20%-30% in post-crizotinib cases").
5. General grammatical errors
6. "Both of crizotinib and lorlatinib were active against TRKA, TRKB and TRKC (Drilon et al, 2018), which were the receptors of nerve growth factors under normal physiologic
7. conditions (Khotskaya et al, 2017)." This is basically incorrect. Lorlatinib and crizotinib are, relatively speaking, poor NTRK inhibitors. Certainly, lorlatinib actually has high degree of selectivity for ROS1 and ALK and has modest to no activity for NTRKs. This section needs to be fact checked.
8. Unlike claimed, ceritinib is not actually highly potent to ROS1, better to say "has been shown to have binding affinity to ROS1".
9. "XMU-MP-5 showed minimal offtargted kinases activity in a KINOMEscan assay and was much more selective than other available ALK TKIs." Apart from the misspelt word, no data or references are presented from other ALK TKIs to support this claim.
10. "XMU-MP-5 showed superior activity against EML4-ALK Ba/F3 cells compared with wild-type Ba/F3 cells with the IC50 ratio of 1553-fold, indicating that XMU-MP-5 might have a broad therapeutic window". Many TKIs exhibit the same sort of insensitivity to wild-type Ba/F3 cells so this piece of data does not reasonably lead to one to believe that XMU-MP-5 might have a broad therapeutic window.
11. Like the introduction, lorlatinib is barely referenced in the discussion even though it is already an FDA-approved inhibitor that has the same efficacy profile against certain hard-to-treat resistant mutations such as G1202R. Some mention of this TKI needs to be included as XMU-MP-5 can be thought of as another option to lorlatinib in the third generation.
Figure Legends
12. Please ensure that Figure legends capture sufficient details that may not be covered adequately in methods.

Referee #3 (Remarks for Author):

This is a manuscript describing the development of a novel ALK inhibitor for the treatment of ALK rearranged lung cancer. The provided the background, structure, in silico binding data, in vitro drug effect, in vivo affection xenografts and GEM models.

Comments:

1- Some additional detail on the medical chemistry work would be helpful
2- The preclinical pharmacology lacks dose - PK data. Is PK linear or non-linear?
3- A dose response curve in xenografts would be helpful.
4- Preclinical toxicology lacks enough detail. Although it is said mice did well with no weight change more details in terms of organ function, e.g. lab values and liver test are needed, Most of the drugs die in preclinical stage in larger animals due to liver toxicology.
5- More details is required on their GEM model. How soon do tumors develop. IS this a model that has been used before, what references?
6- In vivo activity should be compared to other drugs such as lorlatinib as readers would want to know how does this improve the clinical activity compared to loraltinib that also has activity in G1202 resistant mutations.
7- Any details on cell lines that become resistant to their agent? What mechanism of resistance is there?

Point-by-point response to reviewers

"Discovery of XMU-MP-5 Overcoming Resistance to First and Second Generation ALK Inhibitors in NSCLC"

(EMM-2021-14296)

Reply to Reviewer 1:

The manuscript by Lu et al., describes the discovery and development of a novel inhibitor of ALK, which can eliminate cells that are resistant to earlier generations of ALK inhibitors. The novel compound, named XMU-MP-5, could induce apoptosis only in cells expressing mutant ALK and could prevent the growth of ALK-dependent lung tumors in mouse xenograft models as well as GEMM models. Overall, the studies are novel and interesting.

A significant amount of data is presented in this manuscript; they are generally of high quality. Most of the expected experiments have been conducted; these include basic cell biology studies to measure proliferation and apoptosis, biochemical examination of the signaling pathways affected by this compound, determination of the selectivity and specificity, assessment of PK/PD values, in vivo efficacy in mouse models etc. Overall, the study is interesting and novel.

There are two aspects that need attention.

Comments:

1. the data in Supplementary Table 1 shows the efficacy of the identified compound against certain kinases, especially mutant EGFR. It would be interesting and relevant to assess if XMU-MP-5 has comparable efficacy against cell lines harboring standard EGFR mutations like Exon 19 deletion. Further, it appears to be effective against EGFR T790M mutation as well. It would be worthwhile to check if the compound is active against cell lines like H1975 which harbor this gatekeeper mutation.

Response: We thank the reviewer's enthusiasm and quite positive feedback on our work. We took this important suggestion and determined the IC_{50} values of XMU-MP-5 against transformed Ba/F3 cell lines with well-known EGFR mutations

including Exon 19 deletion, L858R and their double mutants with T790M. The result indicates that XMU-MP-5 doesn't exhibit anti-proliferative activity against EGFR mutant Ba/F3 cell lines (**Figure R1**). This result is consistent with our data which previously showed in **Appendix Figure S3B**, that XMU-MP-5 showed negligible anti-proliferative activity against EGFR positive cell lines such as H1299 (EGFR wild-type) and PC9 (Exon 19 deletion) with IC_{50} larger than 10 μM. Collectively, although XMU-MP-5 shows some extent of affinity with EGFR at the concentration of 1 μM *in vitro*, it seems not effective in EGFR positive cell lines.

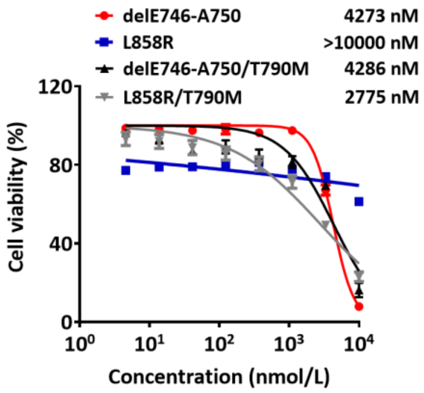

Figure R1. XMU-MP-5 showed negligible anti-proliferative activity against EGFR transformed Ba/F3 cell lines.

Ba/F3 cell lines harboring EGFR deletion, L858R and double mutations were treated with XMU-MP-5 for 48 hours. Cell viability was measured by MTS assay.

2. the data in Supplementary Figure 3 is not clear. There is a fourth panel in the Figure, perhaps a panel D, which is not labeled and not mentioned in the legend. This needs to be examined.

Response: Panel B includes the data of XMU-MP-5 (upper) and crizotinib (below). To make it clearly, we re-organized this panel and updated **Appendix Figure S3**.

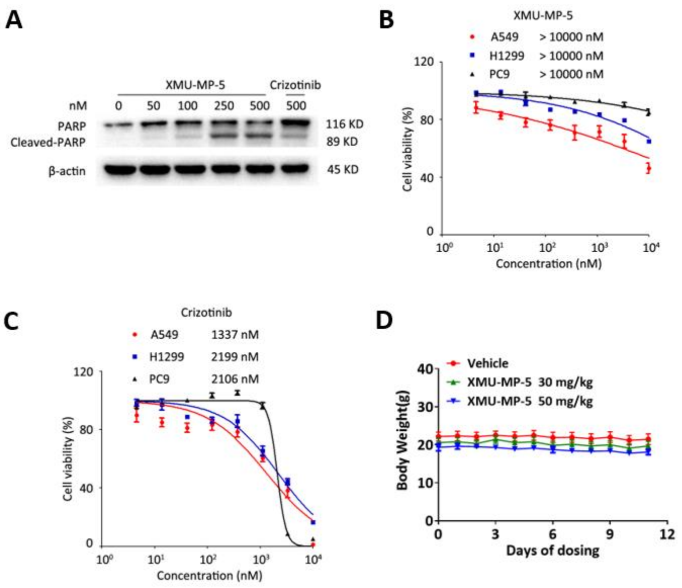

Figure R2. (Appendix Figure S3.) Antiproliferative activity of XMU-MP-5 against non-small cell lung cancer cell lines *in vitro* and *in vivo*.

A, XMU-MP-5 induced cell apoptosis was indicated by cleavage of PARP. H3122 cells were treated as indicated for 24 hours and then analyzed by immunobloting.

B and C XMU-MP-5 showed negligible antiproliferative activity against ALK negative lung cancer cell lines. Cells were treated with series concentrations of XMU-MP-5 for 48 h. Cell viability was detected by MTS assay. Crizotinib was used as reference.

D, No Body weight change was observed after XMU-MP-5 treatment for 11 days. Data were shown as mean ± SD (n = 6).

3. Given the potential to translate this drug into the clinic, it would be worthwhile to examine if XMU-MP-5 affects the viability of primary human lung epithelial cells (small-airway epithelial cells, normal human bronchial epithelial cells etc.) and if a therapeutic window is achievable. The effect of this agent on human T cells and B cells would also be interesting to establish.

Response: Thanks for your suggestion. As shown in **Figure R3**, XMU-MP-5 exhibited negligible activity on human lung fibroblast cell line MRC-5, human

peripheral blood mononuclear cells (PBMC) and human T cells that freshly isolated from healthy donors. These data were updated in **Appendix Figure S4**.

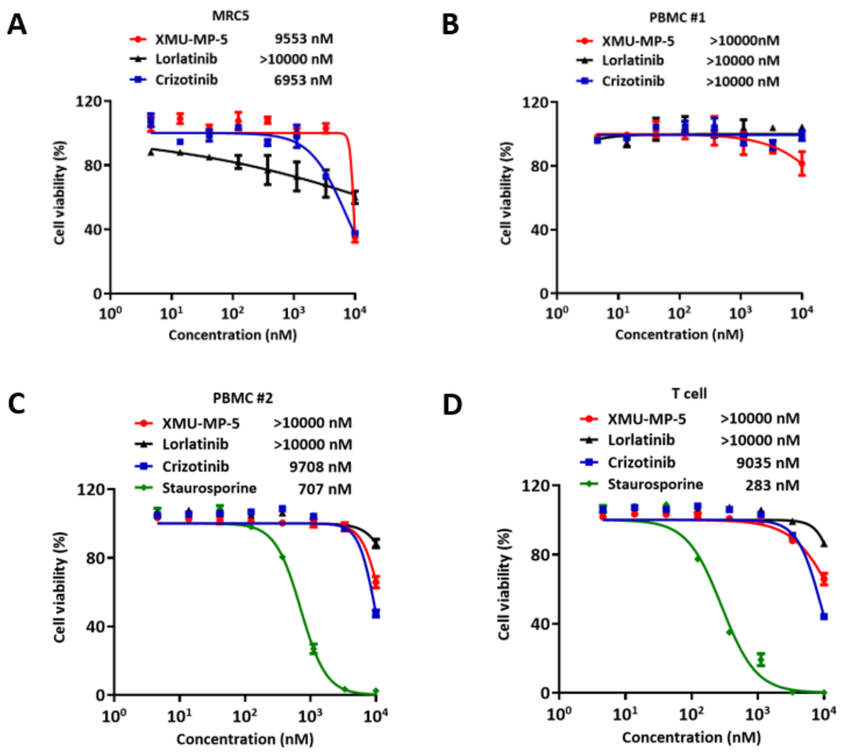

Figure R3. XMU-MP-5 has negligible antiproliferative effect on nontumorigenic cell lines.

Anti-proliferative activities of XMU-MP-5 and reference compounds against human lung fibroblast cell line MRC-5, human peripheral blood mononuclear cells (PBMC) and human T cells that freshly isolated from healthy donors were treated for 48 hours then analyzed by CellTiter-Glo.

Reply to Reviewer 2:

The authors of this manuscript have synthesized and characterized a novel ALK tyrosine kinase inhibitor called XMU-MP-5. They tested this compound in vitro on ALK-positive and negative cancer cell lines and in vivo on ALK-positive xenograft and genetically engineered mouse models. They also performed crystallography to visualize XMU-MP-5 in complex with the ALK kinase domain as well as modeling to predict structural changes that occur within the kinase domain mutations that may affect earlier generation TKIs but not XMU-MP-5. While there are significant revisions that are needed, both in terms of readability, accuracy of claims, and addition of information to the manuscript, this work represents is a novel contribution to the field of targeted therapy. The agent described in this paper can overcome all the known mutations that confer resistance to 1st and 2nd generation ALK TKIs and therefore can be considered a next-generation TKI. I believe this manuscript would be a good fit for EMBO Molecular Medicine contingent on a major revision - with close attention to detail for factual error, grammar/spelling, copy-editing, and interpretation of data.

Comments:

1. The biggest flaw in this section (introduction) is the complete ignorance of lorlatinib, which is also able to overcome L1196M and G1202R in vivo; this must be added to the introduction before it can be published.

Response: Thank you for your suggestion, we revised our manuscript and included lorlatinib in the introduction. Modifications have been highlighted in yellow for your convenience.

2. Figure 1

o May be helpful to show full chemical synthesis process in a Supplemental figure (optional)

Response: The synthetic scheme of XMU-MP-5 has been added in Appendix Figure S11, and the detailed synthetic procedure has been reported in our patent application (US 10,508,118 B2). The citation has been included in Materials and Methods

section.

o Confused as to how you get an IC50 value for XMU-MP-5 in Fig. 1B when the dose response curve does not extend to 50% cell viability. Recommend that this experiment be repeated with lower doses that achieve a sigmoidal curve in order to to properly capture and illustrate the IC50.

Response: We previously obtained the IC_{50} values from the fitting curves calculated by Graphpad Prism 5. We repeated this experiment with extended concentration doses to determine IC_{50} values. Result showed that the IC_{50} of XMU-MP-5 against EML4-ALK Ba/F3 was 4.2 nM while IC_{50} of Crizotinib was 45.9 nM (**Figure R4A**). We also repeated the experiment of Figure 2A to determine the IC_{50}s of XMU-MP-5 and Crizotinib against H3122 cells (**Figure R4B**), which turned out to be 11.9 nM and 252 nM respectively. These data were updated in our revised manuscript.

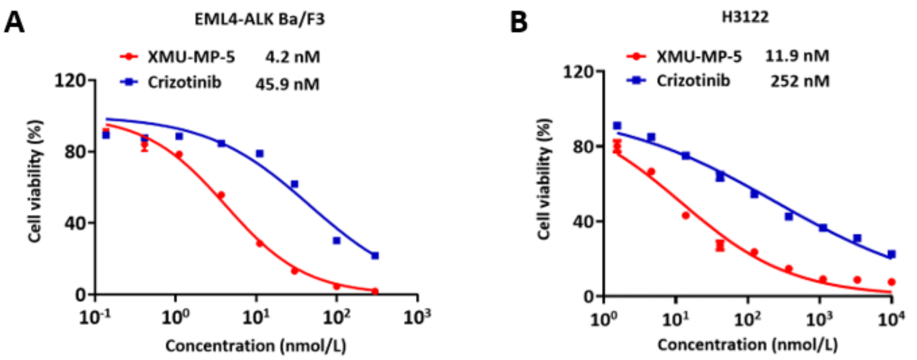

Figure R4. Anti-proliferative activity of XMU-MP-5 against EML4-ALK Ba/F3 and H3122 cell lines.

EML4-ALK Ba/F3 cells were treated for 48 hours while H3122 cells were treated for 72 hours. Cell viability was measured by MTS.

3. Figure 4

o The emphasis on raw IC50 values between drugs is misleading. Greater potency does not necessarily mean more efficacy especially without any analysis of therapeutic index. The better way to compare drugs is to compare fold-change IC50 values of mutant to WT. The raw data in appendix table S4 is good to have alongside

this.

Response: We took your important suggestion and replaced this panel with a bar plot to compare fold change IC_{50} values of ALK mutants transformed Ba/F3 cell lines to wildtype Ba/F3 cell line (**Figure R5**). Raw data were shown in **Appendix Table S4 and S5**. This result indicates that XMU-MP-5 exhibits a larger therapeutic index value than that of crizotinib in all cell lines we have tested.

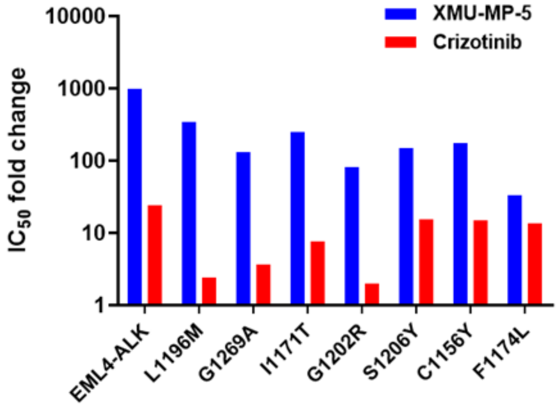

Figure R5. Comparision of fold change IC_{50} values of ALK mutants transformed Ba/F3 cell lines to parental Ba/F3 cell line between XMU-MP-5 and crizotinib.

o The sentence "XMU-MP-5 showed better activity than lorlatinib with IC50 values of 49 nM and 90 nM, respectively" is missing information about what mutants those IC50 values refer to (G1202R). This statement, while mathematically true, is not meaningful in an in vitro or in vivo sense and is frankly misleading: both XMU-MP-5 and lorlatinib exhibit >20 fold increase in G1202R IC50 values and yet both have demonstrated anti-tumor efficacy in vivo as shown in this manuscript and in the Zou et al. ALK lorlatinib manuscript from 2015.

Response: Thanks for pointing out the inaccurate description. We have changed the sentence into "Lorlatinib, the only approved ALK inhibitor that overrides G1202R mutation (Zou *et al*, 2015), had an IC_{50} of 90 nM against G1202R Ba/F3 cell line in our test. XMU-MP-5 also exhibited anti-proliferative potency against G1202R Ba/F3 cell with IC_{50} value of 49 nM (Figure 4B and Appendix Table S4).".

o In panel C (Figure 4), inhibitor dosing is for 7 days only. That seems like a very

short duration for demonstrating durable control over ALK L1196M mutant tumors. Indeed, at both doses tested (30 and 60 mg/kg), there is still tumor growth, albeit attenuated as compared to vehicle.

Response: In the animal experiments, we determined the treatment duration by limiting the maximum tumor volume less than 1500 mm^3, which would follow the guidelines recommended by Institutional Animal Care and Use Committee (IACUC) at Xiamen University. As shown in Figure 4, the xenograft tumors in vehicle group grew so fast and reach the upper limit at day 7, so we could not recommend a longer duration. Although there is still tumor growth in both doses tested in xenograft models, 40 mg/kg bid dosing of XMU-MP-5 for 7 days led to a significant tumor regression in GEM model with ALK-L1196M mutation (**Figure 5 C&D**). As GEM models can better mimic the initiation and progression of tumors in mice than xenograft models, we concluded that XMU-MP-5 has potent anti-tumor activity against L1196M mutation *in vivo*.

o Lorlatinib should have been used in the in vivo experiments to serve as a benchmark considering that it is also a 3rd generation TKI that 1) has already been FDA-approved and that 2) likely has a similar level of efficacy against the same mutations that the authors claim XMU-MP-5 targets.

Response: Thanks for your suggestion, we repeated this experiment and used lorlatinib as a positive control.

As expected, lorlatinib showed promising anti-tumor activity against G1202R mutant in mouse xenograft model at the dose of 20 mg/kg per day for a 9 days duration. It is the most important advantages compared to second generation ALK inhibitors. Administration of 30 mg/kg XMU-MP-5 also greatly attenuated tumor growth. Treatment with 30 mg/kg of XMU-MP-5 twice daily led to a comparable tumor growth inhibition as 20 mg/kg dosing of lorlatinib. It’s also worth mentioning that the molar concentration of 30 mg/kg of XMU-MP-5 (HCl salt) is nearly equal to that of 20 mg/kg of lorlatinib. No body weight loss was observed in all group tested.

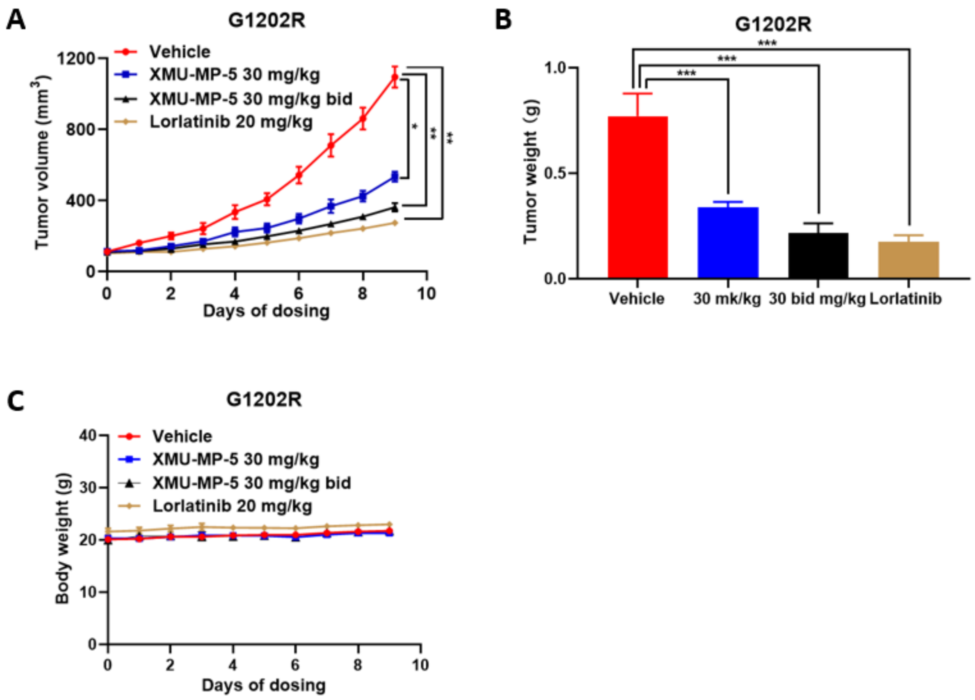

Figure R6. (updated Figure 4E&F) *In vivo* efficacy of XMU-MP-5 in G1202R Ba/F3 xenograft mouse model.

A and B, BALB/c nude mice bearing G1202R Ba/F3 cells xenograft tumors were administrated with 30 mg/kg XMU-MP-5 by tail vein injection once and twice daily respectively and 20 mg/kg lorlatinib by oral administration. Tumor volume and tumor weight were shown as mean ± SEM (n = 6).

C, Body weights of each group bearing G1202R xenograft tumors were measured every day. Data were shown as mean ± SD (n = 6).

Data information: Statistical analysis were performed by two-tailed, unpaired Student's *t* test or one-way ANOVA with Dunnett test. *P < 0.05; **P < 0.01; ***P < 0.001.

4. Discussion needs citations (i.e., when saying that "secondary mutations account for 20%-30% in post-crizotinib cases").

Response: Thank you for pointing out. We have updated the references accordingly.

5. General grammatical errors

Response: We have ordered Springer Nature Author Services and had our entire

manuscript edited for grammar, phrasing, and punctuation. Modifications have been highlighted in yellow. These efforts have greatly improved the language in our manuscript. The editing certificate is attached in Appendix I.

6. "Both of crizotinib and lorlatinib were active against TRKA, TRKB and TRKC (Drilon et al, 2018), which were the receptors of nerve growth factors under normal physiologic conditions (Khotskaya et al, 2017)." This is basically incorrect. Lorlatinib and crizotinib are, relatively speaking, poor NTRK inhibitors. Certainly, lorlatinib actually has high degree of selectivity for ROS1 and ALK and has modest to no activity for NTRKs. This section needs to be fact checked.

Response: Thanks for pointing out. We have changed the description accordingly.

7. Unlike claimed, ceritinib is not actually highly potent to ROS1, better to say "has been shown to have binding affinity to ROS1".

Response: We have revised this sentence accordingly.

8. "XMU-MP-5 showed minimal offtargted kinases activity in a KINOMEscan assay and was much more selective than other available ALK TKIs." Apart from the misspelt word, no data or references are presented from other ALK TKIs to support this claim.

Response: We made this conclusion by comparing the selectivity of XMU-MP-5 we tested to the information of other ALK inhibitors collected from references. The KINOMEscan results are reported as the percentage of the DMSO negative control signal (ctrl%) set at 100%, and a lower number represents higher-affinity binding (Karaman *et al*, 2008). At a concentration of 1 μM of XMU-MP-5, there were 10 kinases with ctrl ≤10% in total including ALK, indicating strong inhibition. A selectivity score [S(10)] of 0.02 was determined for XMU-MP-5, calculated by dividing the number of kinases with ctrl ≤10% (n = 10) by the total number of kinases tested (n = 468). The lower score represents the better selectivity. Comparing with the reported data for ceritinib (Jang *et al*, 2017), alectinib (Sakamoto *et al*, 2011), and

brigatinib (Zhang *et al*, 2016), XMU-MP-5 has the lowest score S(10). Meanwhile, XMU-MP-5 has a lower S(35) of 0.03 with just 16 kinase hits with ctrl ≤35% while S(35) of crizotinib is 0.21 with 94 kinases with ctrl ≤35% (Huber *et al*, 2014). The S scores were summarized in **Table R1** and included in **Appendix Table S2**.

Table R1 S value of XMU-MP-5 and approved ALK TKIs

TKI	S(10)	S(35)
XMU-MP-5	0.02	0.03
Crizotinib	ND	0.21
Ceritinib	0.19	ND
Alectinib	0.07	ND
Brigatinib	0.34	ND

Karaman MW, Herrgard S, Treiber DK, Gallant P, Atteridge CE, Campbell BT, Chan KW, Ciceri P, Davis MI, Edeen PT, Faraoni R, Floyd M, Hunt JP, Lockhart DJ, Milanov ZV, Morrison MJ, Pallares G, Patel HK, Pritchard S, Wodicka LM et al. (2008) A quantitative analysis of kinase inhibitor selectivity. Nature biotechnology 26: 127-32

Jang J, Son JB, To C, Bahcall M, Kim SY, Kang SY, Mushajiang M, Lee Y, Jänne PA, Choi HG, Gray NS (2017) Discovery of a Potent Dual ALK and EGFR T790M Inhibitor. *Eur J Med Chem* 136: 497–510.

Sakamoto H, Tsukaguchi T, Hiroshima S, Kodama T, Kobayashi T, Fukami TA, Oikawa N, Tsukuda T, Ishii N, Aoki Y (2011) CH5424802, a selective ALK inhibitor capable of blocking the resistant gatekeeper mutant. *Cancer Cell* 19:679-690

Zhang S, Anjum R, Squillace R, Nadworny S, Zhou TJ, Keats J, Ning YY, Wardwell SD, Miller D, Song YC, Eichinger L, Moran L, Huang WS, Liu SY, Zou D, Wang YH, Mohemmad Q, Jang HG, Ye E, Narasimhan N, Wang F, Miret J, Zhu XT, Clackson T, Dalgarno D, Shakespeare WC, Rivera VM (2016) The Potent ALK Inhibitor Brigatinib (AP26113) Overcomes Mechanisms of Resistance to First- and Second-Generation ALK Inhibitors in Preclinical Models. *Clin Cancer*

Res 10.1158/1078-0432.CCR-16-0569.

Huber KVM, Salah E, Radic B, Gridling M, Elkins JM, Stukalov A, Jemth A, Gokturk C, Sanjiv K, Strömberg K, Pham T, Berglund UW, Colinge J, Bennett KL, Loizou J, Helleday T, Knapp S, Superti-Furga G (2014) Stereospecific targeting of MTH1 by (*S*)-crizotinib as anticancer strategy. *Nature* 508(7495): 222–227.

9. "XMU-MP-5 showed superior activity against EML4-ALK Ba/F3 cells compared with wild-type Ba/F3 cells with the IC50 ratio of 1553-fold, indicating that XMU-MP-5 might have a broad therapeutic window". Many TKIs exhibit the same sort of insensitivity to wild-type Ba/F3 cells so this piece of data does not reasonably lead to one to believe that XMU-MP-5 might have a broad therapeutic window.

Response: To be prudent, we have deleted the statement accordingly.

10. Like the introduction, lorlatinib is barely referenced in the discussion even though it is already an FDA-approved inhibitor that has the same efficacy profile against certain hard-to-treat resistant mutations such as G1202R. Some mention of this TKI needs to be included as XMU-MP-5 can be thought of as another option to lorlatinib in the third generation.

Response: We have added information of lorlatinib in discussion section.

11. Please ensure that Figure legends capture sufficient details that may not be covered adequately in methods.

Response: We have checked the legends to include sufficient details.

Reply to Reviewer 3:

This is a manuscript describing the development of a novel ALK inhibitor for the treatment of ALK rearranged lung cancer. The provided the background, structure, in silico binding data, in vitro drug effect, in vivo affection xenografts and GEM models.

Comments:

1. Some additional detail on the medical chemistry work would be helpful

Response: We're preparing the medicinal chemistry paper reporting the detailed structure and activity relationship (SAR) study, which will be published in due course.

2. The preclinical pharmacology lacks dose - PK data. Is PK linear or non-linear?

Response:

Thanks for your suggestion. We have collected mouse PK data at the doses of 5 mg/kg, 30mg/kg and 60 mg/kg via intravenous (IV). As showed in **Figure R7** and **Table R2**, blood drug concentrations of XMU-MP-5 showed a linear increase at each time points. These data were updated in **Appendix Table S3**.

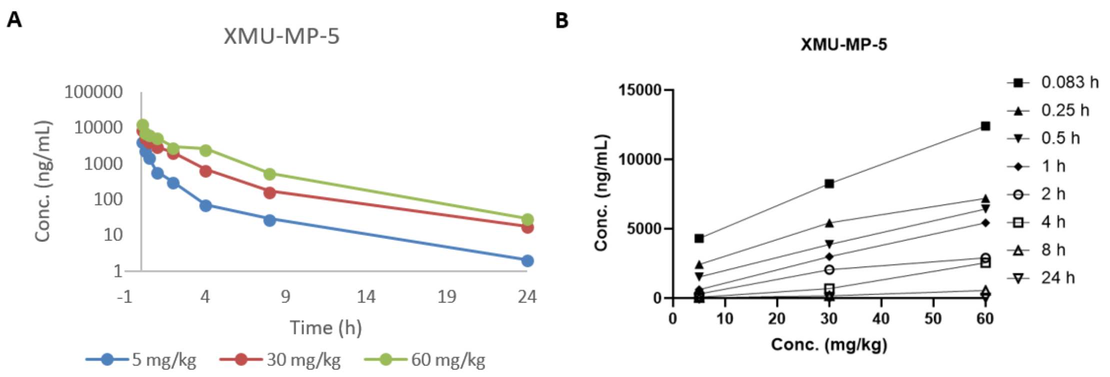

Figure R7. Dose-PK data of XMU-MP-5 by IV administration.

Blood drug concentrations at doses of 5 mg/kg, 30 mg/kg and 60 mg/kg XMU-MP-5 by IV at different time points in mice. Data were shown as mean, n = 3.

Table R2 Blood drug concentrations (ng/mL)

Time (h)	5 mg/kg	30 mg/kg	60 mg/kg
**0.083**	4303.44	8260	12400
**0.25**	2428.55	5430	7190
**0.5**	1530.83	3870	6430
**1**	612.93	2990	5430
**2**	308.29	2060	2910
**4**	72.84	688	2580
**8**	28.69	171	555
**24**	2.09	17.9	29.6

3. A dose response curve in xenografts would be helpful.

Response: Thanks for your suggestion, we repeated this experiment with two doses of XMU-MP-5 and used lorlatinib as a positive control (**Figure R6**, the same as the response to the reviewer #2).

As expected, lorlatinib showed promising anti-tumor activity against G1202R mutant in mouse xenograft model at the dose of 20 mg/kg per day for a 9 days duration. It is the most important advantages compared to the second generation ALK inhibitors. Administration of 30 mg/kg XMU-MP-5 also greatly attenuated tumor growth. Treatment with 30 mg/kg of XMU-MP-5 twice daily led to a comparable tumor growth inhibition as 20 mg/kg dosing of lorlatinib. It's also worth mentioning that the molar concentration of 30 mg/kg of XMU-MP-5 (HCl salt) is nearly equal to that of 20 mg/kg of lorlatinib. No body weight loss was observed in all group tested.

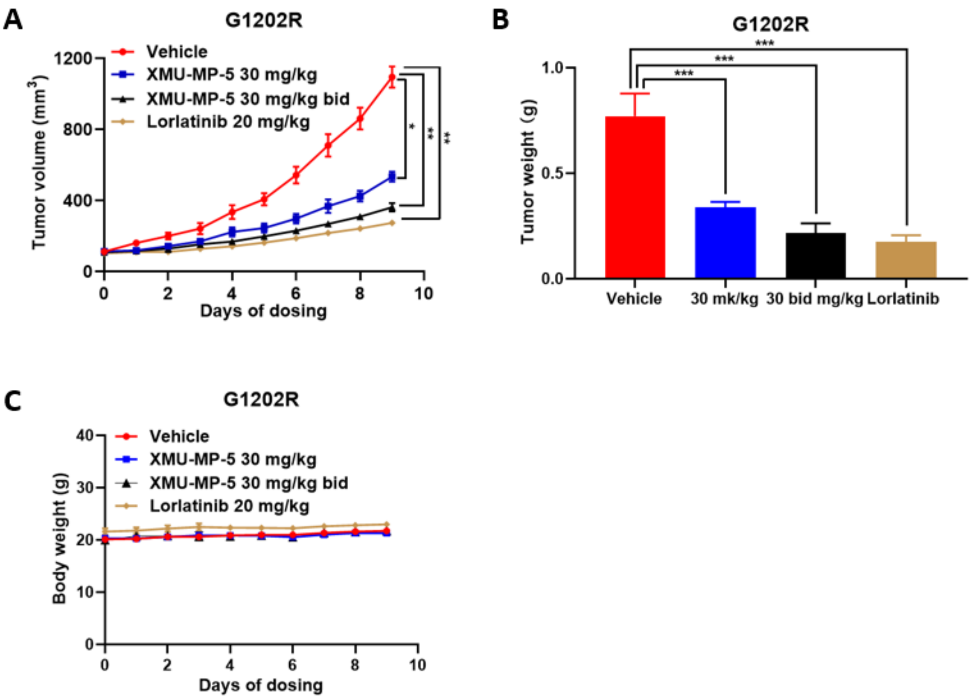

Figure R6. (updated Figure 4E&F) *In vivo* efficacy of XMU-MP-5 in G1202R Ba/F3 xenograft mouse model.

A, B BALB/c nude mice bearing G1202R Ba/F3 cells xenograft tumors were administrated with 30 mg/kg XMU-MP-5 by tail vein injection once and twice daily respectively and 20 mg/kg lorlatinib by oral administration. Tumor volume and tumor weight were shown as mean ± SEM (n = 6).

C Body weights of each group bearing G1202R xenograft tumors were measured every day. Data were shown as mean ± SD (n = 6).

Data information: Statistical analysis were performed by two-tailed, unpaired Student's *t* test or one-way ANOVA with Dunnett test. *P < 0.05; **P < 0.01; ***P < 0.001.

4. Preclinical toxicology lacks enough detail. Although it is said mice did well with no weight change more details in terms of organ function, e.g. lab values and liver test are needed, most of the drugs die in preclinical stage in larger animals due to liver toxicology.

Response: Thanks for your suggestion. We carried out a more detailed evaluation of the toxicity of XMU-MP-5 using ICR mice. The results showed that dosing of 60

mg/kg once daily by IV for 10 days didn't lead to the elevation of alanine transaminase (ALT) or aspartate transaminase (AST) values (**Figure R8A&B**). There was no body weight loss observed (**Figure R8C)**, and H&E staining of organs looked normal (**Figure R8D)**. These data indicated that 60 mg/kg of XMU-MP-5 (IV dosing) is well tolerated and may not lead to significant toxicity in our study.

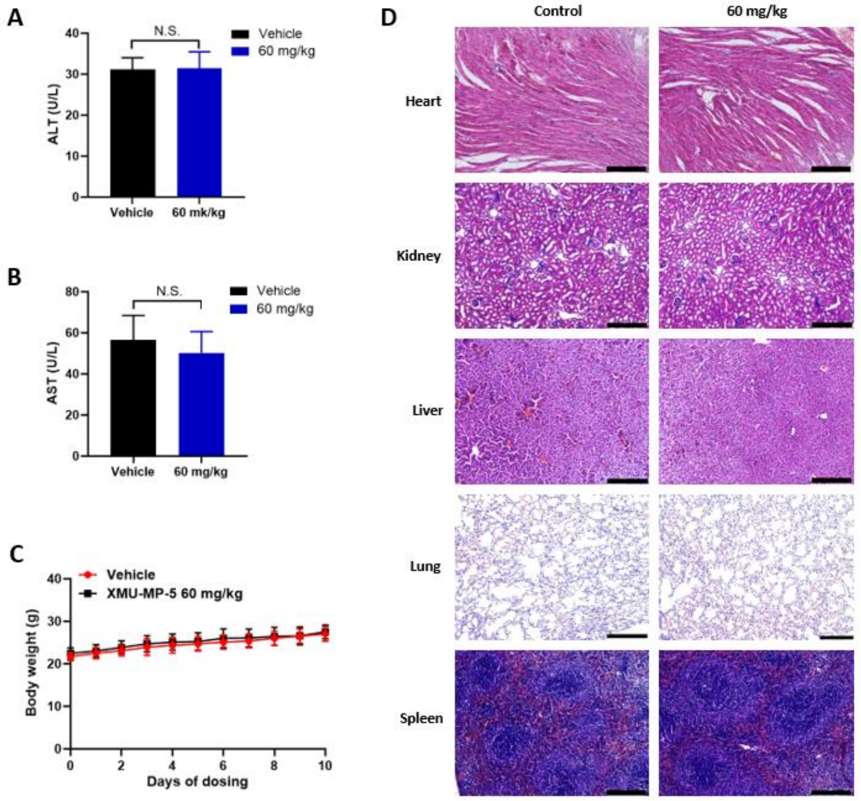

Figure R8. Toxicity evaluation of XMU-MP-5 in ICR mice.

A, B The activity of ALT and AST after 10 days' dosing were measured. Mice were administrated with vehicle or 60 mg/kg XMU-MP-5 by tail vein injection (IV) for 10 days. ALT and AST data were shown as Mean ± SD (n=7).

C Body weights of each group were measured every day. Data were shown as mean ± SD (n = 7).

D Representative image of heart, kidney, liver, lung and spleen analyzed by H&E staining. Scale bars, 250 μm.

Data information: Statistical analysis was obtained from unpaired Student's *t* test. N.S. means not significant.

5. More details is required on their GEM model. How soon do tumors develop. Is this a model that has been used before, what references?

Response: The GEM model was used to study the pathogenesis of NSCLC and provide experimental support for the treatment of this intractable cancer with ALK inhibitors (Chen et al, 2010; Chen et al, 2014 and Soda et al, 2008). Generally, the GEM models are genetically engineered mice harboring a doxycycline-inducible EML4-ALK fusion gene. These mice were imaged using CT scan to document tumor burden after 3 weeks of doxycycline exposure. After another two weeks, XMU-MP-5 was administrated by IV for 3 weeks. Lung tumors were imaged by CT scan. The references were all cited in our manuscript and listed below for your convenience.

Soda M, Takada S, Takeuchi K, Choi YL, Enomoto M, Ueno T, Haruta H, Hamada T, Yamashita Y, Ishikawa Y, Sugiyama Y, Mano H (2008) A mouse model for EML4-ALK-positive lung cancer. *Proc Natl Acad Sci U S A* 105:19893-19897

Chen Z, Akbay E, Mikse O, Tupper T, Cheng K, Wang Y, Tan X, Altabef A, Woo SA, Chen L, Reibel JA, Janne PA, Sharpless NE, Engelman JA, Shapiro GI, Kung AL, Wong KK (2014) Co-clinical trials demonstrate superiority of crizotinib to chemotherapy in ALK-rearranged non-small cell lung cancer and predict strategies to overcome resistance. *Clin Cancer Res* 20:1204-1211

Chen Z, Sasaki T, Tan X, Carretero J, Shimamura T, Li D, Xu C, Wang Y, Adelmant GO, Capelletti M, Lee HJ, Rodig SJ, Borgman C, Park SL, Kim HR, Padera R, Marto JA, Gray NS, Kung AL, Shapiro GI, Jänne PA, Wong KK (2010) Inhibition of ALK, PI3K/MEK, and HSP90 in murine lung adenocarcinoma induced by EML4-ALK fusion oncogene. *Cancer Res* 70:9827-9836

6. In vivo activity should be compared to other drugs such as lorlatinib as readers would want to know how does this improve the clinical activity compared to loraltinib that also has activity in G1202 resistant mutations.

Response: We repeated this experiment and used lorlatinib as a positive control. The results and responses were shown in **Figure R6 (updated Figure 4E&F)**.

7. Any details on cell lines that become resistant to their agent? What mechanism of resistance is there?

Response:

XMU-MP-5 is a highly selective ALK inhibitor that showed promising activity on ALK positive NSCLC cells but negligible effect on ALK negative cell lines. In our transformed Ba/F3 system, we found that F1174L, compared to other ALK mutants, may exhibit resistance to XMU-MP-5. Through structural analysis, we observed that D1270 in F1174L structure has been approaching to the binding site of XMU-MP-5 by overlapping our crystal structure of XMU-MP-5 and ALK with a published F1174L structure (PDB: 2YJR). The hydrophilic oxygen of D1270 may mutually exclusive with the hydrophobic isopropyl of XMU-MP-5 (**Figure R9**). Furthermore, F1174L mutation may also stabilize an active conformation that increase the ATP-binding affinity of ALK (Gainor *et al*, 2016). Together, we assume that both factors contribute to the low efficacy of XMU-MP-5 to F1174L.

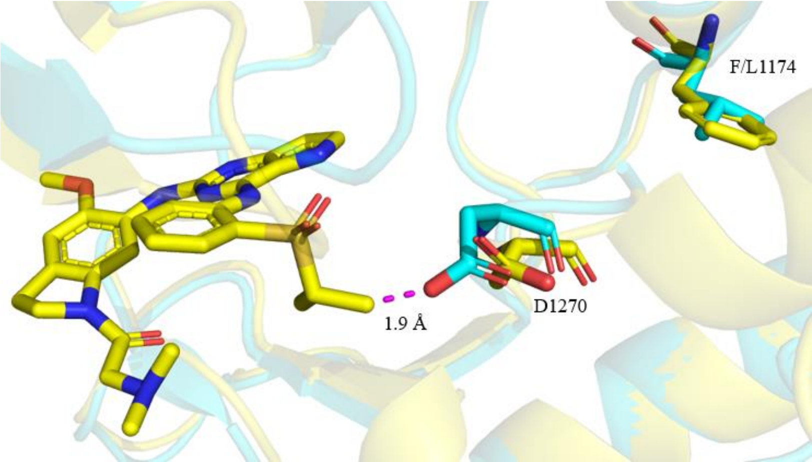

Figure R9. Structure basis of the low activity of XMU-MP-5 against F1174L mutation.

Structural analysis by comparing the binding mode of XMU-MP-5 with wild-type ALK (yellow) and F1174L (cyan) (PDB ID: 2YJR).

Gainor JF, Dardaei L, Yoda S, Friboulet L, Leshchiner I, Katayama R, Dagogo-Jack I, Gadgeel S, Schultz K, Singh M, Chin E, Parks M, Lee D, DiCecca RH, Lockerman E, Huynh T, Logan J, Ritterhouse LL, Le LP, Muniappan A, Digumarthy S, Channick C, Keyes C, Getz G, Dias-Santagata D, Heist RS, Lennerz J, Sequist LV, Benes CH, Iafrate AJ, Mino-Kenudson M, Engelman JA, Shaw AT (2016) Molecular Mechanisms of Resistance to First- and Second-Generation ALK Inhibitors in ALK-Rearranged Lung Cancer. *Cancer Discov.* 6(10):1118-1133

11th Oct 2021

Dear Prof. Deng,

Thank you for the submission of your revised manuscript to EMBO Molecular Medicine. We have received the enclosed reports from referees #2 and #3 who re-reviewed your manuscript, and as you will see, they are now supportive of publication. I am therefore pleased to inform you that we will be able to accept your manuscript once the following minor editorial points will be addressed:

1/ Main manuscript text:
- Thank you for addressing the queries from our data editors. Please clearly indicate biological vs. technical replicates (Figures 1B, 2A, 4A).
- Would you agree to replace the current title with the following: "A new ALK inhibitor overcomes resistance to first- and second-generation inhibitors in NSCLC"?
- Please remove the yellow highlights in the text, and only keep in track changes mode any new modification.
- Material and methods:
o Cells: please indicate the origin of the cells, and whether cells were authenticated (when applicable) and tested for mycoplasma contamination.
o Colony formation assay: please indicate the concentration of paraformaldehyde and crystal violet staining procedure.
o Western Blot: please indicate how cell lysates were prepared for immunoblotting. Please provide the antibody dilutions.
o Mice: please indicate the source and gender of the mice. Please also indicate the housing and husbandry conditions.
o Immunochemistry: please indicate the antibody dilutions.
- Authors' contributions: We note that you currently have together with you a total of 4 co-corresponding authors. Is that correct? Do you confirm equal contribution of these 4 people, able to take full responsibility for the paper and its content? While there is not limit per se to the number of co-corresponding authors, 3 is rare, 4 even more so, and may not reflect as intended to the community. Moreover, please note that all corresponding authors are required to supply an ORCID ID for their name upon submission of a revised manuscript. An ORCID identifier is currently missing for Cai-Hong Yun.

2/ Figures:
- Please indicate in the main and appendix figures or in their legends the exact n and p= values (including for non-significant p values, ns), not a range, along with the statistical test used.
- Please add a reference to Figure 4G in the main manuscript text.
- Appendix: Fig. S1, S8: please add a legend. Fig. S4: please define the error bars, e.g. SD. Please define the number and the nature, i.e. biological or technical, of the replicates. Appendix Table S2: please define ND.

3/ Source Data:
Thank you for providing source data. Please upload them to have 1 file per figure.
Regarding Appendix Figure S8: It is unclear whether the proteins and phosphor-proteins were detected from the same blots (after stripping), and whether the different proteins were detected on different blots, please clarify.

4/ Please provide a synopsis text and figure. Synopses are displayed on the journal webpage and are freely accessible to all readers. They include a short stand first (maximum of 300 characters, including space) as well as 2-5 one-sentences bullet points that summarizes the paper. Please write the bullet points to summarize the key NEW findings. They should be designed to be complementary to the abstract - i.e. not repeat the same text. We encourage inclusion of key acronyms and quantitative information (maximum of 30 words / bullet point). Please use the passive voice. Please attach these in a separate file or send them by email, we will incorporate them accordingly.
Please also suggest a striking image or visual abstract to illustrate your article as a PNG file 550 px wide x 300-600 px high.

5/ As part of the EMBO Publications transparent editorial process initiative (see our Editorial at http://embomolmed.embopress.org/content/2/9/329), EMBO Molecular Medicine will publish online a Review Process File (RPF) to accompany accepted manuscripts.
In the event of acceptance, this file will be published in conjunction with your paper and will include the anonymous referee reports, your point-by-point response and all pertinent correspondence relating to the manuscript. Let us know whether you agree with the publication of the RPF and as here, IF YOU WANT TO REMOVE OR NOT ANY FIGURES from it prior to publication.
Please note that the Authors checklist will be published at the end of the RPF.

I look forward to receiving your revised manuscript.

Yours sincerely,

Lise Roth

Lise Roth, PhD
Editor
EMBO Molecular Medicine

To submit your manuscript, please follow this link:

Link Not Available

*Additional important information regarding Figures

Each figure should be given in a separate file and should have the following resolution:
Graphs 800-1,200 DPI
Photos 400-800 DPI
Colour (only CMYK) 300-400 DPI"

Figures are not edited by the production team. All lettering should be the same size and style; figure panels should be indicated by capital letters (A, B, C etc). Gridlines are not allowed except for log plots. Figures should be numbered in the order of their appearance in the text with Arabic numerals. Each Figure must have a separate legend and a caption is needed for each panel.

*Additional important information regarding figures and illustrations can be found at https://bit.ly/EMBOPressFigurePreparationGuideline. See also figure legend preparation guidelines: https://www.embopress.org/page/journal/17574684/authorguide#figureformat

The system will prompt you to fill in your funding and payment information. This will allow Wiley to send you a quote for the article processing charge (APC) in case of acceptance. This quote takes into account any reduction or fee waivers that you may be eligible for. Authors do not need to pay any fees before their manuscript is accepted and transferred to our publisher.

***** Reviewer's comments *****

Referee #2 (Comments on Novelty/Model System for Author):

The authors report the discovery and validation of a novel ALK inhibitor with potential to mitigate recurrent resistant mutations that pose clinical challenges for first generation ALK inhibitors such as crizotinib. It is uncertain if this inhibitor will compete in the market place with lorlatinib, as lorlatinib already has FDA approval to some extent. However, the purpose of this paper is to unveil new medicinal chemistry and biology, and as such as appropriate for publication.

Referee #2 (Remarks for Author):

I appreciate that the authors took efforts to thoroughly address the majority of my comments, with either additional experiments, presentation of data in alternative ways. In addition, the authors have improved the readability by employing the services of Springer Nature Author Services.
I am satisfied with the effort put in by the authors to address concerns and this manuscript is now suitable for publication.

Referee #3 (Comments on Novelty/Model System for Author):

Adequate systems

Referee #3 (Remarks for Author):

From my standpoint all queries were adequately answered.

The authors performed the requested editorial changes.

3rd Nov 2021

Dear Prof. Deng,

Thank you for submitting your revised files. We are pleased to inform you that your manuscript is accepted for publication and is now being sent to our publisher to be included in the next available issue of EMBO Molecular Medicine.

Please read below for additional IMPORTANT information regarding your article, its publication and the production process.

Congratulations on your interesting work!

Yours sincerely,

Lise Roth

Lise Roth, Ph.D
Scientific Editor
EMBO Molecular Medicine

Follow us on Twitter @EmboMolMed
Sign up for eTOCs at embopress.org/alertsfeeds

EMBO PRESS

YOU MUST COMPLETE ALL CELLS WITH A PINK BACKGROUND ➔

PLEASE NOTE THAT THIS CHECKLIST WILL BE PUBLISHED ALONGSIDE YOUR PAPER

Corresponding Author Name: Xianming Deng
Journal Submitted to: Embo Molecular Medicine
Manuscript Number: EMM-2021-14296

USEFUL LINKS FOR COMPLETING THIS FORM

http://www.antibodypedia.com
http://1degreebio.org
http://www.equator-network.org/reporting-guidelines/improving-bioscience-research-repor
http://grants.nih.gov/grants/olaw/olaw.htm
http://www.mrc.ac.uk/Ourresearch/Ethicsresearchguidance/Useofanimals/index.htm
http://ClinicalTrials.gov
http://www.consort-statement.org
http://www.consort-statement.org/checklists/view/32-consort/66-title
http://www.equator-network.org/reporting-guidelines/reporting-recommendations-for-tumo
http://datadryad.org
http://figshare.com
http://www.ncbi.nlm.nih.gov/gap
http://www.ebi.ac.uk/ega
http://biomodels.net/
http://biomodels.net/miriam/
http://jjj.biochem.sun.ac.za
https://osp.od.nih.gov/biosafety-biosecurity-and-emerging-biotechnology/
http://www.selectagents.gov/

Reporting Checklist For Life Sciences Articles (Rev. June 2017)

This checklist is used to ensure good reporting standards and to improve the reproducibility of published results. These guidelines are consistent with the Principles and Guidelines for Reporting Preclinical Research issued by the NIH in 2014. Please follow the journal's authorship guidelines in preparing your manuscript.

A- Figures
1. Data
The data shown in figures should satisfy the following conditions:

- the data were obtained and processed according to the field's best practice and are presented to reflect the results of the experiments in an accurate and unbiased manner.
- figure panels include only data points, measurements or observations that can be compared to each other in a scientifically meaningful way.
- graphs include clearly labeled error bars for independent experiments and sample sizes. Unless justified, error bars should not be shown for technical replicates.
- if n< 5, the individual data points from each experiment should be plotted and any statistical test employed should be justified
- Source Data should be included to report the data underlying graphs. Please follow the guidelines set out in the author ship guidelines on Data Presentation.

2. Captions

Each figure caption should contain the following information, for each panel where they are relevant:

- a specification of the experimental system investigated (eg cell line, species name).
- the assay(s) and method(s) used to carry out the reported observations and measurements
- an explicit mention of the biological and chemical entity(ies) that are being measured.
- an explicit mention of the biological and chemical entity(ies) that are altered/varied/perturbed in a controlled manner.
- the exact sample size (n) for each experimental group/condition, given as a number, not a range;
- a description of the sample collection allowing the reader to understand whether the samples represent technical or biological replicates (including how many animals, litters, cultures, etc.).
- a statement of how many times the experiment shown was independently replicated in the laboratory.
- definitions of statistical methods and measures:
  - common tests, such as t-test (please specify whether paired vs. unpaired), simple $\chi 2$ tests, Wilcoxon and Mann-Whitney tests, can be unambiguously identified by name only, but more complex techniques should be described in the methods section;
  - are tests one-sided or two-sided?
  - are there adjustments for multiple comparisons?
  - exact statistical test results, e.g., P values = x but not P values < x;
  - definition of 'center values' as median or average;
  - definition of error bars as s.d. or s.e.m.

Any descriptions too long for the figure legend should be included in the methods section and/or with the source data.

In the pink boxes below, please ensure that the answers to the following questions are reported in the manuscript itself. Every question should be answered. If the question is not relevant to your research, please write NA (non applicable). We encourage you to include a specific subsection in the methods section for statistics, reagents, animal models and human subjects.

B- Statistics and general methods

Please fill out these boxes ➔ (Do not worry if you cannot see all your text once you press return)

1.a. How was the sample size chosen to ensure adequate power to detect a pre-specified effect size?	Sample size was selected based on preliminary experiments, which allowed to estimate the ability to distinguish a true effect (statistical power) over the risk of false positives (significance threshold). Important criteria we considered are data variability and the size of the effect desired.
1.b. For animal studies, include a statement about sample size estimate even if no statistical methods were used.	We have included a statement about the sample size in each group.
2. Describe inclusion/exclusion criteria if samples or animals were excluded from the analysis. Were the criteria pre-established?	No exclusion.
3. Were any steps taken to minimize the effects of subjective bias when allocating animals/samples to treatment (e.g. randomization procedure)? If yes, please describe.	The tumor-bearing animals were randomly assigned to the treatment or control group.
For animal studies, include a statement about randomization even if no randomization was used.	We have included a statement noting that the tumor-bearing mice were randomely assigned to the control and treatment groups.
4.a. Were any steps taken to minimize the effects of subjective bias during group allocation or/and when assessing results (e.g. blinding of the investigator)? If yes please describe.	To minimize subjective bias when assessing results, we have used objective tumor pictures and tumor weight.
4.b. For animal studies, include a statement about blinding even if no blinding was done	We have added a statement that the animal studies were not conducted blindly.
5. For every figure, are statistical tests justified as appropriate?	Yes. Statistical comparison between only two groups were done with the unpaired Student's t test. Multiple group comparisons were completed using one-way analysis of variance (ANOVA).These information is included in a Statistical Analysis section.
Do the data meet the assumptions of the tests (e.g., normal distribution)? Describe any methods used to assess it.	Normal distribution was assumed. The data presented in this publication did not specifically test for normal distribution.

Is there an estimate of variation within each group of data?	Yes.The variance between the groups that are being statistically compared are similar and have been calculated.
Is the variance similar between the groups that are being statistically compared?	Yes.

C- Reagents

6. To show that antibodies were profiled for use in the system under study (assay and species), provide a citation, catalog number and/or clone number, supplementary information or reference to an antibody validation profile. e.g., Antibodypedia (see link list at top right), 1DegreeBio (see link list at top right).	We provide the details of each antibody used in Materials and Methods.
7. Identify the source of cell lines and report if they were recently authenticated (e.g., by STR profiling) and tested for mycoplasma contamination.	We identify the source of cell lines. All cell lines were tested mycoplasma-negative.

* for all hyperlinks, please see the table at the top right of the document

D- Animal Models

8. Report species, strain, gender, age of animals and genetic modification status where applicable. Please detail housing and husbandry conditions and the source of animals.	We provide all required information for the animals in Materials and Methods..
9. For experiments involving live vertebrates, include a statement of compliance with ethical regulations and identify the committee(s) approving the experiments.	All animal care and experimental procedures complied with the guidelines from the Institutional Animal Care and Use Committee at Experimental Animal Centre at Xiamen University. For the GEMM models, all experimental protocols were approved by the Institutional Committee for Animal Care and Use at Jinan University. All animal work was performed in strict accordance with the approved protocol.
10. We recommend consulting the ARRIVE guidelines (see link list at top right) (PLoS Biol. 8(6), e1000412, 2010) to ensure that other relevant aspects of animal studies are adequately reported. See author guidelines, under 'Reporting Guidelines'. See also: NIH (see link list at top right) and MRC (see link list at top right) recommendations. Please confirm compliance.	All animal studies complied with ARRIVE guidelines.

E- Human Subjects

11. Identify the committee(s) approving the study protocol.	N/A
12. Include a statement confirming that informed consent was obtained from all subjects and that the experiments conformed to the principles set out in the WMA Declaration of Helsinki and the Department of Health and Human Services Belmont Report.	N/A
13. For publication of patient photos, include a statement confirming that consent to publish was obtained.	N/A
14. Report any restrictions on the availability (and/or on the use) of human data or samples.	N/A
15. Report the clinical trial registration number (at ClinicalTrials.gov or equivalent), where applicable.	N/A
16. For phase II and III randomized controlled trials, please refer to the CONSORT flow diagram (see link list at top right) and submit the CONSORT checklist (see link list at top right) with your submission. See author guidelines, under 'Reporting Guidelines'. Please confirm you have submitted this list.	N/A
17. For tumor marker prognostic studies, we recommend that you follow the REMARK reporting guidelines (see link list at top right). See author guidelines, under 'Reporting Guidelines'. Please confirm you have followed these guidelines.	N/A

F- Data Accessibility

18: Provide a "Data Availability" section at the end of the Materials & Methods, listing the accession codes for data generated in this study and deposited in a public database (e.g. RNA-Seq data: Gene Expression Omnibus GSE39462, Proteomics data: PRIDE PXD000208 etc.) Please refer to our author guidelines for 'Data Deposition'.<br><br>Data deposition in a public repository is mandatory for:<br>a. Protein, DNA and RNA sequences<br>b. Macromolecular structures<br>c. Crystallographic data for small molecules<br>d. Functional genomics data<br>e. Proteomics and molecular interactions	Data Availability section is provided. The crystal structure has been deposited in Protein Data Bank (PDB) with the accession ID 7BTT.
19. Deposition is strongly recommended for any datasets that are central and integral to the study; please consider the journal's data policy. If no structured public repository exists for a given data type, we encourage the provision of datasets in the manuscript as a Supplementary Document (see author guidelines under 'Expanded View' or in unstructured repositories such as Dryad (see link list at top right) or Figshare (see link list at top right).	N/A
20. Access to human clinical and genomic datasets should be provided with as few restrictions as possible while respecting ethical obligations to the patients and relevant medical and legal issues. If practically possible and compatible with the individual consent agreement used in the study, such data should be deposited in one of the major public access-controlled repositories such as dbGAP (see link list at top right) or EGA (see link list at top right).	N/A
21. Computational models that are central and integral to a study should be shared without restrictions and provided in a machine-readable form. The relevant accession numbers or links should be provided. When possible, standardized format (SBML, CellML) should be used instead of scripts (e.g. MATLAB). Authors are strongly encouraged to follow the MIRIAM guidelines (see link list at top right) and deposit their model in a public database such as Biomodels (see link list at top right) or JWS Online (see link list at top right). If computer source code is provided with the paper, it should be deposited in a public repository or included in supplementary information.	N/A

G- Dual use research of concern

22. Could your study fall under dual use research restrictions? Please check biosecurity documents (see link list at top right) and list of select agents and toxins (APHIS/CDC) (see link list at top right). According to our biosecurity guidelines, provide a statement only if it could.	No.
